# Supplementary material for: Nitric oxide controls proliferation of Leishmania major by inhibiting the recruitment of permissive host cells
Source: Immunity. 2021 Dec 14;54(12):2724–2739.e10. doi: 10.1016/j.immuni.2021.09.021 (PMC8691385; doi:10.1016/j.immuni.2021.09.021)
Supplement: Document S1. Figures S1–S7 and Tables S1–S6 [file mmc1.pdf]

Immunity, Volume *54*

Supplemental information

Nitric oxide controls proliferation of *Leishmania major* by inhibiting the recruitment of permissive host cells

Pauline Formaglio, Mohamad Alabdullah, Anastasios Siokis, Juliane Handschuh, Ina Sauerland, Yan Fu, Anna Krone, Patricia Gintschel, Juliane Stettin, Sandrina Heyde, Juliane Mohr, Lars Philipsen, Anja Schröder, Philippe A. Robert, Gang Zhao, Sahamoddin Khailaie, Anne Dudeck, Jessica Bertrand, Gerald F. Späth, Sascha Kahlfuß, Philippe Bousso, Burkhart Schraven, Jochen Huehn, Sebastian Binder, Michael Meyer-Hermann, and Andreas J. Müller

Supplemental Materials

Supplemental table 1. Spearman correlations for high dose infection models. Related to figure 1.

Supplemental table 2. Spearman correlations for low dose infection models. Related to figure 1.

Supplemental table 3. Model selection for high dose infection models. Related to figure 3.

Supplemental table 4. Model selection for low dose infection models. Related to figure 3.

Supplemental table 5. Estimated parameters for the high dose infection models. Related to figure 3.

Supplemental table 6. Estimated parameters for the low dose infection models. Related to figure 3.

Supplemental figure S1. ODE models for *L. major* infection. Related to figure 1.

Supplemental figure S2. *In vitro* characterization of cell death reporter parasite *L. major*^{necR}. Related to figure 2.

Supplemental figure S3. Analysis of parasite death in the ongoing infection. Related to figure 3.

Supplemental figure S4. Analysis of parasite proliferation in the ongoing infection. Related to figure 4.

Supplemental figure S5. Inhibition of cell recruitment to the site of infection. Related to figure 5.

Supplemental figure S6. Pathogen burden development under L-NIL with and without blocking of immune cell recruitment. Related to figure 6.

Supplemental figure S7. Ectopic phagocyte entry in late *L. major* infection. Related to figure 7.

Supplemental table 1. Spearman correlations for high dose infection models. Related to figure 1.

	k_{Mrec}	α	S_M	k_i	k_a	k_{ai}	k_{ia}	k_P	k_r	k_D	d_M	d_{Mi}	d_{Mai}	d_{Ma}	d_P	c_P	c_{im}	$M(0)$	$P(0)$	$D(0)$	Model
k_{Mrec}	1.000	-0.132	-0.003	-0.319	0.288	-0.053	0.007	-0.151	-0.239	0.122	-0.014	-0.076	0.135	0.636	0.095	0.069	0.086	-0.105	-0.002	0.172	1
α	-0.132	1.000	0.023	0.032	0.126	0.050	-0.106	-0.163	-0.015	-0.103	-0.060	-0.024	0.092	0.133	0.119	-0.021	-0.081	-0.033	-0.028	0.068	1
S_M	-0.003	0.023	1.000	0.052	-0.082	0.040	-0.010	-0.037	0.071	0.100	-0.121	-0.073	0.071	-0.099	-0.073	0.120	0.123	0.089	0.025	0.004	1
k_i	-0.319	0.032	0.052	1.000	-0.100	0.135	0.014	-0.164	0.051	0.133	0.060	-0.075	-0.085	-0.162	0.064	-0.022	-0.030	0.015	-0.198	0.169	1
k_a	0.288	0.126	-0.082	-0.100	1.000	-0.053	0.051	-0.227	0.000	-0.012	0.028	-0.012	0.073	-0.011	0.029	-0.005	0.098	-0.040	-0.206	0.012	1
k_{ai}	-0.053	0.050	0.040	0.135	-0.053	1.000	0.011	0.305	0.057	-0.057	0.006	0.002	0.012	-0.102	0.045	0.091	-0.016	0.010	0.175	-0.096	1
k_{ia}	0.007	-0.106	-0.010	0.014	0.051	0.011	1.000	0.101	-0.003	-0.022	-0.010	0.117	-0.154	-0.086	0.141	0.017	0.063	0.006	-0.013	-0.024	1
k_P	-0.151	-0.163	-0.037	-0.164	-0.227	0.305	0.101	1.000	0.429	0.036	-0.040	0.048	-0.322	-0.051	0.024	-0.023	-0.041	0.087	-0.195	0.154	1
k_r	-0.239	-0.015	0.071	0.051	0.000	0.057	-0.003	0.429	1.000	-0.172	-0.082	-0.075	0.139	-0.212	0.033	-0.050	-0.054	0.123	-0.258	-0.111	1
k_D	0.122	-0.103	0.100	0.133	-0.012	-0.057	-0.022	0.036	-0.172	1.000	-0.039	-0.091	-0.086	0.057	0.061	0.131	0.055	-0.133	-0.370	-0.137	1
d_M	-0.014	-0.060	-0.121	0.060	0.028	0.006	-0.010	-0.040	-0.082	-0.039	1.000	0.059	-0.033	0.060	-0.059	-0.072	-0.008	-0.002	-0.053	-0.055	1
d_{Mi}	-0.076	-0.024	-0.073	-0.075	-0.012	0.002	0.117	0.048	-0.075	-0.091	0.059	1.000	-0.027	-0.041	0.052	-0.020	0.027	0.052	0.083	-0.018	1
d_{Mai}	0.135	0.092	0.071	-0.085	0.073	0.012	-0.154	-0.322	0.139	-0.086	-0.033	-0.027	1.000	0.192	0.005	0.054	-0.020	-0.050	-0.048	-0.072	1
d_{Ma}	0.636	0.133	-0.099	-0.162	-0.011	-0.102	-0.086	-0.051	-0.212	0.057	0.060	-0.041	0.192	1.000	0.068	-0.013	-0.087	-0.052	0.087	0.068	1
d_P	0.095	0.119	-0.073	0.064	0.029	0.045	0.141	0.024	0.033	0.061	-0.059	0.052	0.005	0.068	1.000	-0.019	0.168	0.093	-0.009	0.038	1
c_P	0.069	-0.021	0.120	-0.022	-0.005	0.091	0.017	-0.023	-0.050	0.131	-0.072	-0.020	0.054	-0.013	-0.019	1.000	-0.037	-0.001	0.141	-0.047	1
c_{im}	0.086	-0.081	0.123	-0.030	0.098	-0.016	0.063	-0.041	-0.054	0.055	-0.008	0.027	-0.020	-0.087	0.168	-0.037	1.000	0.098	-0.090	-0.135	1
$M(0)$	-0.105	-0.033	0.089	0.015	-0.040	0.010	0.006	0.087	0.123	-0.133	-0.002	0.052	-0.050	-0.052	0.093	-0.001	0.098	1.000	0.036	-0.016	1
$P(0)$	-0.002	-0.028	0.025	-0.198	-0.206	0.175	-0.013	-0.195	-0.258	-0.370	-0.053	0.083	-0.048	0.087	-0.009	0.141	-0.090	0.036	1.000	0.113	1
$D(0)$	0.172	0.068	0.004	0.169	0.012	-0.096	-0.024	0.154	-0.111	-0.137	-0.055	-0.018	-0.072	0.068	0.038	-0.047	-0.135	-0.016	0.113	1.000	1
k_{Mrec}	1.000	0.020	0.019	0.007	0.016	0.041	-0.094	0.038	0.010	-0.029	-0.007	-0.021	-0.010	0.189	-0.028	0.014	-0.016	-0.017	-0.099	-0.075	4
α	0.020	1.000	0.274	0.109	0.311	0.168	0.162	-0.005	0.058	0.242	0.000	0.167	0.418	0.132	-0.088	0.252	-0.119	-0.108	-0.323	-0.129	4
S_M	0.019	0.274	1.000	0.112	0.008	-0.057	0.023	-0.023	-0.034	0.048	0.027	-0.005	0.117	0.012	-0.131	-0.110	0.046	-0.075	0.194	0.003	4
k_i	0.007	0.109	0.112	1.000	-0.079	0.047	0.076	-0.253	0.076	0.266	0.076	0.044	0.026	-0.103	0.034	-0.143	-0.014	0.119	-0.201	0.134	4
k_a	0.016	0.311	0.008	-0.079	1.000	-0.099	-0.048	0.077	-0.370	0.107	-0.091	0.143	0.052	-0.123	-0.025	0.056	0.031	-0.075	0.127	0.115	4
k_{ai}	0.041	0.168	-0.057	0.047	-0.099	1.000	0.004	-0.129	-0.104	-0.038	0.092	0.093	-0.013	0.125	-0.020	0.302	0.269	-0.007	-0.442	-0.279	4
k_{ia}	-0.094	0.162	0.023	0.076	-0.048	0.004	1.000	-0.170	0.113	0.006	-0.112	0.107	0.030	0.022	0.069	-0.127	-0.026	0.098	-0.056	0.017	4
k_P	0.038	-0.005	-0.023	-0.253	0.077	-0.129	-0.170	1.000	0.409	0.087	-0.013	-0.073	-0.047	0.013	0.022	-0.337	-0.043	-0.078	-0.053	0.161	4
k_r	0.010	0.058	-0.034	0.076	-0.370	-0.104	0.113	0.409	1.000	-0.024	0.081	-0.032	0.023	-0.020	0.019	0.112	0.075	-0.010	-0.326	-0.116	4
k_D	-0.029	0.242	0.048	0.266	0.107	-0.038	0.006	0.087	-0.024	1.000	0.131	0.087	0.004	-0.113	0.005	-0.081	0.099	0.109	-0.165	-0.175	4
d_M	-0.007	0.000	0.027	0.076	-0.091	0.092	-0.112	-0.013	0.081	0.131	1.000	-0.053	0.066	0.018	-0.028	0.031	0.093	-0.085	-0.157	-0.196	4
d_{Mi}	-0.021	0.167	-0.005	0.044	0.143	0.093	0.107	-0.073	-0.032	0.087	-0.053	1.000	0.076	-0.011	0.040	0.031	0.113	-0.021	-0.177	-0.078	4
d_{Mai}	-0.010	0.418	0.117	0.026	0.052	-0.013	0.030	-0.047	0.023	0.004	0.066	0.076	1.000	0.087	-0.118	0.147	-0.035	-0.141	-0.190	-0.088	4
d_{Ma}	0.189	0.132	0.012	-0.103	-0.123	0.125	0.022	0.013	-0.020	-0.113	0.018	-0.011	0.087	1.000	-0.027	-0.037	0.042	-0.072	-0.043	-0.005	4
d_P	-0.028	-0.088	-0.131	0.034	-0.025	-0.020	0.069	0.022	0.019	0.005	-0.028	0.040	-0.118	-0.027	1.000	-0.034	0.017	-0.028	0.067	0.069	4
c_P	0.014	0.252	-0.110	-0.143	0.056	0.302	-0.127	-0.337	0.112	-0.081	0.031	0.031	0.147	-0.037	-0.034	1.000	-0.100	-0.015	-0.157	-0.159	4
c_{im}	-0.016	-0.119	0.046	-0.014	0.031	0.269	-0.026	-0.043	0.075	0.099	0.093	0.113	-0.035	0.042	0.017	-0.100	1.000	0.007	0.063	-0.055	4
$M(0)$	-0.017	-0.108	-0.075	0.119	-0.075	-0.007	0.098	-0.078	-0.010	0.109	-0.085	-0.021	-0.141	-0.072	-0.028	-0.015	0.007	1.000	-0.028	0.004	4
$P(0)$	-0.099	-0.323	0.194	-0.201	0.127	-0.442	-0.056	-0.053	-0.326	-0.165	-0.157	-0.177	-0.190	-0.043	0.067	-0.157	0.063	-0.028	1.000	0.609	4
$D(0)$	-0.075	-0.129	0.003	0.134	0.115	-0.279	0.017	0.161	-0.116	-0.175	-0.196	-0.078	-0.088	-0.005	0.069	-0.159	-0.055	0.004	0.609	1.000	4

Correlations between the parameter vectors generated from bootstrapping in high dose models 1 and 4 analyzed by Spearman's rank correlation. Color coding range: -0.372 (white) to 1 (blue) for model 1, -0.448 (white) to 1 (blue) for model 4.

Supplemental table 2. Spearman correlations for low dose infection models. Related to figure 1.

	k_{Mrec}	α	S_M	k_i	k_a	k_{ai}	k_{ia}	k_P	k_r	k_D	d_M	d_{Mi}	d_{Mai}	d_{Ma}	d_P	c_P	c_{im}	$M(0)$	$P(0)$	$D(0)$	Model
k_{Mrec}	1.000	-0.069	-0.107	0.006	-0.031	0.125	-0.040	-0.011	-0.032	-0.065	0.024	-0.005	0.051	-0.020	-0.013	-0.062	-0.008	0.099	0.013	0.040	1
α	-0.069	1.000	0.340	0.056	0.173	-0.040	-0.003	-0.079	-0.064	-0.042	0.050	-0.144	0.019	0.496	-0.134	-0.119	-0.147	0.079	-0.365	-0.007	1
S_M	-0.107	0.340	1.000	-0.023	0.026	0.057	-0.010	0.054	0.054	-0.023	0.047	-0.143	0.005	0.087	-0.115	0.008	-0.008	0.126	0.057	0.056	1
k_i	0.006	0.056	-0.023	1.000	0.051	-0.077	-0.021	-0.324	-0.224	0.076	0.053	0.049	0.177	0.084	-0.010	-0.313	0.071	-0.036	-0.395	0.110	1
k_a	-0.031	0.173	0.026	0.051	1.000	0.011	0.002	-0.201	0.015	-0.163	0.003	-0.079	0.010	0.215	-0.157	-0.147	0.282	-0.036	-0.151	0.113	1
k_{ai}	0.125	-0.040	0.057	-0.077	0.011	1.000	-0.088	0.206	-0.305	0.050	-0.021	0.025	-0.043	-0.156	0.117	0.060	0.037	-0.041	-0.031	0.014	1
k_{ia}	-0.040	-0.003	-0.010	-0.021	0.002	-0.088	1.000	0.112	-0.174	0.147	0.055	0.008	-0.035	0.001	-0.070	-0.002	0.069	0.004	0.031	0.085	1
k_P	-0.011	-0.079	0.054	-0.324	-0.201	0.206	0.112	1.000	0.207	0.056	-0.087	-0.011	-0.297	-0.114	0.134	-0.041	-0.243	-0.156	-0.278	0.142	1
k_r	-0.032	-0.064	0.054	-0.224	0.015	-0.305	-0.174	0.207	1.000	-0.150	-0.151	-0.091	0.277	-0.074	-0.011	0.096	0.176	-0.039	-0.056	-0.059	1
k_D	-0.065	-0.042	-0.023	0.076	-0.163	0.050	0.147	0.056	-0.150	1.000	-0.074	0.019	-0.073	0.086	-0.026	-0.303	0.152	0.055	-0.234	-0.128	1
d_M	0.024	0.050	0.047	0.053	0.003	-0.021	0.055	-0.087	-0.151	-0.074	1.000	0.017	0.020	0.022	-0.034	-0.009	-0.089	-0.023	0.067	-0.031	1
d_{Mi}	-0.005	-0.144	-0.143	0.049	-0.079	0.025	0.008	-0.011	-0.091	0.019	0.017	1.000	0.079	-0.018	0.010	0.050	0.010	-0.024	0.058	-0.032	1
d_{Mai}	0.051	0.019	0.005	0.177	0.010	-0.043	-0.035	-0.297	0.277	-0.073	0.020	0.079	1.000	0.105	-0.014	-0.123	-0.054	-0.033	0.082	-0.111	1
d_{Ma}	-0.020	0.496	0.087	0.084	0.215	-0.156	0.001	-0.114	-0.074	0.086	0.022	-0.018	0.105	1.000	-0.158	-0.213	-0.047	-0.020	-0.138	0.044	1
d_P	-0.013	-0.134	-0.115	-0.010	-0.157	0.117	-0.070	0.134	-0.011	-0.026	-0.034	0.010	-0.014	-0.158	1.000	0.103	-0.078	-0.075	0.009	-0.007	1
c_P	-0.062	-0.119	0.008	-0.313	-0.147	0.060	-0.002	-0.041	0.096	-0.303	-0.009	0.050	-0.123	-0.213	0.103	1.000	0.008	0.013	0.226	-0.101	1
c_{im}	-0.008	-0.147	-0.008	0.071	0.282	0.037	0.069	-0.243	0.176	0.152	-0.089	0.010	-0.054	-0.047	-0.078	0.008	1.000	0.114	0.219	-0.023	1
$M(0)$	0.099	0.079	0.126	-0.036	-0.036	-0.041	0.004	-0.156	-0.039	0.055	-0.023	-0.024	-0.033	-0.020	-0.075	0.013	0.114	1.000	0.096	0.001	1
$P(0)$	0.013	-0.365	0.057	-0.395	-0.151	-0.031	0.031	-0.278	-0.056	-0.234	0.067	0.058	0.082	-0.138	0.009	0.226	0.219	0.096	1.000	-0.123	1
$D(0)$	0.040	-0.007	0.056	0.110	0.113	0.014	0.085	0.142	-0.059	-0.128	-0.031	-0.032	-0.111	0.044	-0.007	-0.101	-0.023	0.001	-0.123	1.000	1
k_{Mrec}	1.000	-0.286	0.117	-0.062	0.047	-0.090	0.026	-0.070	0.009	-0.006	-0.079	-0.040	0.004	0.081	0.028	0.011	0.013	-0.011	0.092	0.045	4
α	-0.286	1.000	-0.003	0.021	0.316	0.050	0.053	-0.127	-0.102	0.024	0.172	-0.020	0.054	0.545	0.031	-0.051	-0.111	-0.190	-0.026	0.008	4
S_M	0.117	-0.003	1.000	-0.058	-0.077	-0.049	0.020	-0.002	-0.009	0.151	-0.061	-0.013	-0.053	0.010	0.111	-0.106	-0.076	-0.009	0.070	0.066	4
k_i	-0.062	0.021	-0.058	1.000	0.143	-0.160	0.012	-0.436	0.013	-0.060	0.020	0.082	0.205	-0.023	-0.039	-0.294	0.064	-0.038	-0.268	0.025	4
k_a	0.047	0.316	-0.077	0.143	1.000	-0.104	-0.055	-0.201	-0.248	-0.239	-0.022	0.005	0.143	0.294	0.012	-0.201	0.335	-0.061	-0.178	0.072	4
k_{ai}	-0.090	0.050	-0.049	-0.160	-0.104	1.000	0.092	0.144	-0.276	0.033	0.037	-0.011	-0.237	-0.133	0.115	0.249	0.035	0.053	0.026	-0.033	4
k_{ia}	0.026	0.053	0.020	0.012	-0.055	0.092	1.000	-0.064	0.119	-0.007	-0.005	-0.077	0.007	0.051	0.057	0.132	-0.042	0.024	0.026	0.063	4
k_P	-0.070	-0.127	-0.002	-0.436	-0.201	0.144	-0.064	1.000	0.026	0.049	0.000	0.000	-0.185	-0.055	0.115	-0.082	-0.151	0.048	-0.149	0.138	4
k_r	0.009	-0.102	-0.009	0.013	-0.248	-0.276	0.119	0.026	1.000	0.099	-0.040	-0.025	0.035	0.012	-0.063	0.354	0.203	0.007	-0.175	0.108	4
k_D	-0.006	0.024	0.151	-0.060	-0.239	0.033	-0.007	0.049	0.099	1.000	0.059	-0.035	-0.221	0.157	-0.001	-0.137	0.038	-0.009	-0.246	-0.067	4
d_M	-0.079	0.172	-0.061	0.020	-0.022	0.037	-0.005	0.000	-0.040	0.059	1.000	0.011	-0.022	-0.014	0.067	0.021	-0.006	-0.080	-0.086	-0.085	4
d_{Mi}	-0.040	-0.020	-0.013	0.082	0.005	-0.011	-0.077	0.000	-0.025	-0.035	0.011	1.000	0.055	-0.072	-0.080	-0.053	0.089	-0.007	0.000	-0.011	4
d_{Mai}	0.004	0.054	-0.053	0.205	0.143	-0.237	0.007	-0.185	0.035	-0.221	-0.022	0.055	1.000	0.111	0.019	-0.004	0.101	-0.059	-0.083	-0.054	4
d_{Ma}	0.081	0.545	0.010	-0.023	0.294	-0.133	0.051	-0.055	0.012	0.157	-0.014	-0.072	0.111	1.000	0.065	-0.060	-0.015	-0.040	0.049	0.058	4
d_P	0.028	0.031	0.111	-0.039	0.012	0.115	0.057	0.115	-0.063	-0.001	0.067	-0.080	0.019	0.065	1.000	-0.025	0.000	-0.154	0.056	-0.020	4
c_P	0.011	-0.051	-0.106	-0.294	-0.201	0.249	0.132	-0.082	0.354	-0.137	0.021	-0.053	-0.004	-0.060	-0.025	1.000	-0.090	-0.041	0.122	-0.052	4
c_{im}	0.013	-0.111	-0.076	0.064	0.335	0.035	-0.042	-0.151	0.203	0.038	-0.006	0.089	0.101	-0.015	0.000	-0.090	1.000	0.090	-0.060	-0.011	4
$M(0)$	-0.011	-0.190	-0.009	-0.038	-0.061	0.053	0.024	0.048	0.007	-0.009	-0.080	-0.007	-0.059	-0.040	-0.154	-0.041	0.090	1.000	-0.112	0.023	4
$P(0)$	0.092	-0.026	0.070	-0.268	-0.178	0.026	0.026	-0.149	-0.175	-0.246	-0.086	0.000	-0.083	0.049	0.056	0.122	-0.060	-0.112	1.000	-0.054	4
$D(0)$	0.045	0.008	0.066	0.025	0.072	-0.033	0.063	0.138	0.108	-0.067	-0.085	-0.011	-0.054	0.058	-0.020	-0.052	-0.011	0.023	-0.054	1.000	4

Correlations between the parameter vectors generated from bootstrapping in low dose models 1 and 4 analyzed by Spearman's rank correlation. Color coding range: -0.395 (white) to 1 (blue) for model 1, -0.435 (white) to 1 (blue) for model 4.

Supplemental table 3. Model selection for high dose infection models. Related to figure 3.

	P (# params.)	RSS	AICc	Δ(AICc) = M4(best)-MX	$e^{2*\Delta(\text{AICc})}$	Status
M1	20	1.38284	159.18	1.66	27.5279	Cannot be rejected
M2	20	2.76479	164.92	7.39	2.62E+06	Rejected due to $e^{2*\Delta(\text{AICc})}$
M3	21	0.970483	182.38	24.85	3.87E+21	Rejected due to $e^{2*\Delta(\text{AICc})}$
M4	20	0.504625	157.53	0.00	1.0000	Best
M5	20	2.06314	161.53	4.00	2.99E+03	Rejected due to $e^{2*\Delta(\text{AICc})}$
M6	21	1.73764	184.46	26.93	2.47E+23	Rejected due to $e^{2*\Delta(\text{AICc})}$

Supplemental table 4. Model selection for low dose infection models. Related to figure 3.

	P (# params.)	RSS	AICc	Δ(AICc) = M4(best)-M_X	$e^{2*\Delta(\mathrm{AICc})}$	Status
M1	24	0.774539	209.0638719	0.108564339	1.242503975	Cannot be rejected
M2	24	1.46628	210.6139383	1.658630715	27.58470437	Rejected due to bad fit of death reporter
M3	25	0.638547	240.0383702	31.08306261	9.96334E+26	Rejected due to $e^{2*\Delta(\mathrm{AICc})}$
M4	24	0.700961	208.9553075	0	1	Best
M5	24	1.24545	210.0151069	1.059799379	8.327795356	Rejected due to bad fit of death reporter
M6	25	0.893855	240.4296046	31.4742971	2.17885E+27	Rejected due to $e^{2*\Delta(\mathrm{AICc})}$

Supplemental table 5. Estimated parameters for the high dose infection models. Related to figure 3.

	M1	M2	M3	M4	M5	M6	Lower bound	Upper bound	
k_{Mrec}	6720.98	22.3587	131.481	150.353	1586.96	1.51174	0.75127	13778.4	Estimated
α	3325.33	10272.2	8490.96	10766.3	4892.54	8204.17	1484.1	16500.8	Estimated
S_M	2861.09	13750.2	24467.8	36132.5	12592.9	38974.8	1341.78	69952.9	Estimated
k_i	2.32E-07	4.23E-08	8.80E-08	6.89E-08	4.67E-08	6.15E-08	1.55E-08	3.60E-07	Estimated
k_a	2562.36	4623.33	2441.12	5369.19	2260.13	3408.12	989.025	6346.5	Estimated
k_{ai}	15658.9	9606.75	9473.94	7109.04	5509.81	7178.53	2807.55	32612	Estimated
k_{ia}	4.82E-07	2.42E-06	4.28E-07	1.48E-06	4.49E-07	8.40E-07	1.49E-07	4.34E-06	Estimated
k_P	1.89E-04	7.19E-07	1.57E-04	1.00E-04	4.23E-02	2.93E-03	4.35E-07	6.59E-02	Estimated
k_r	5.04E-04	-	3.89E-04	7.88E-05	-	1.30E-05	4.69E-06	7.87E-04	Estimated
k_D	1.54E-06	1.32E-06	2.44E-07	1.66E-06	9.95E-07	9.62E-07	1.64E-07	2.70E-06	Estimated
d_M	1.26E-07	7.80E-05	2.39E-07	7.28E-04	1.36E-06	1.02E-05	8.69E-08	9.37E-04	Estimated
d_{Mi}	3.11E-07	3.82E-07	6.66E-08	3.07E-07	2.08E-07	1.87E-06	3.83E-08	3.88E-06	Estimated
d_{Mai}	1.13637	0.243529	1.28927	3.09039	9.56808	8.76902	0.137962	23.9283	Estimated
d_{Ma}	1.02593	0.537217	0.534353	0.203856	0.395066	0.238159	0.084326	2.24136	Estimated
d_P	9.27E-03	3.04E-02	1.64E-02	8.86E-04	1.24E-01	0.0016496	7.06E-04	0.128902	Estimated
c_P	3.25E+06	1.09E+07	2.11E+06	1.88E+06	1.12E+06	1.62E+06	406256	11138500	Estimated
c_{im}	6.43374	5133.66	3445.86	8470.43	81.2739	8865.47	3.17876	20645.7	Estimated
β	-	5.55E-05	0.148554	-	-	-	1.00E-07	1.5	Estimated
c_{iP}	-	-	-	-	0.0147527	0.0207067	7.55E-03	59272.1	Estimated
$M(0)$	3224.42	3.35713	5.82213	17.0632	170.318	8690.27	1	11714.7	Estimated
$M_i(0)$	0.0	0.0	0.0	0.0	0.0	0.0	0	0	Estimated
$M_{ai}(0)$	0.0	0.0	0.0	0.0	0.0	0.0	0	0	Estimated
$M_a(0)$	0.0	0.0	0.0	0.0	0.0	0.0	0	0	Estimated
$P(0)$	9822.03	73737.5	30814.1	54215.1	22869.1	12282.2	1000	100000	Estimated
$D(0)$	0.0932255	0.108552	0.0471096	7.07E-03	2.60E-05	1.62E-04	1.00E-05	1.1	Estimated

Supplemental table 6. Estimated parameters for the low dose infection models. Related to figure 3.

	M1	M2	M3	M4	M5	M6	Lower bound	Upper bound	
k_{Mrec}	1.35924	1.75111	6.15908	938.91	356.249	172.026	0.559785	1072.71	Estimated
α	3157.12	3016.22	3423.29	2484.91	3975.56	4337.72	1097.9	6787.62	Estimated
S_{M}	12108.7	8756.37	11180.4	3991.47	13670.1	10446.7	1878.32	40498.9	Estimated
k_{i}	5.60E-08	9.41E-08	8.97E-08	7.45E-08	6.62E-08	5.40E-08	1.98E-08	1.12E-07	Estimated
k_{a}	1252.25	1351.19	1735.94	317.774	1603.01	1568.01	142.701	4434.23	Estimated
k_{ai}	8772.05	14014.8	18304.6	1647.12	12820.3	9002.05	1342.38	22539.3	Estimated
k_{ia}	8.40E-07	4.34E-07	1.24E-06	1.15E-07	2.90E-06	4.49E-07	4.10E-08	3.02E-06	Estimated
k_{P}	5.25E-05	5.12E-05	7.67E-05	1.18E-04	5.44E-03	9.55E-03	4.47E-05	2.28E-02	Estimated
k_{r}	1.26E-04	-	1.43E-04	2.77E-05	-	6.51E-06	4.01E-06	3.76E-04	Estimated
k_{D}	1.34E-06	2.50E-06	1.02E-06	1.43E-06	3.07E-06	2.36E-06	5.70E-07	4.64E-06	Estimated
d_{M}	7.78E-04	2.49E-03	7.03E-04	3.81E-03	3.74E-03	4.01E-03	4.97E-04	9.52E-03	Estimated
d_{Mi}	4.75E-06	1.18E-05	1.79E-05	6.82E-05	6.96E-06	5.85E-06	2.27E-06	6.87E-05	Estimated
d_{Mai}	1.50006	1.75289	2.27229	1.31671	2.64091	1.40927	0.476245	2.96067	Estimated
d_{Ma}	1.46654	1.35232	1.47093	1.94352	1.33362	1.9146	0.660975	2.78754	Estimated
d_{P}	2.76E-03	2.76E-03	2.80E-03	7.70E-05	3.00E-02	8.08E-03	3.24E-05	3.74E-02	Estimated
c_{P}	183269	93422.8	170755	146714	813192	506376	41526.3	837261	Estimated
c_{im}	15741.3	13581.7	23632.1	1447.27	19100.7	12264.5	1443.48	36022.9	Estimated
β	-	3.60E-02	9.42E-06	-	-	-	3.37E-06	1.5	Estimated
c_{iP}	-	-	-	-	0.013912	1.02309	1.38E-02	59272.1	Estimated
$M(0)$	0.781359	5567.73	3299.72	18493.5	2984.04	1025.89	0.52049	28128.5	Estimated
$M_{\mathrm{i}}(0)$	0.0	0.0	0.0	0.0	0.0	0.0	0	0	Estimated
$M_{\mathrm{ai}}(0)$	0.0	0.0	0.0	0.0	0.0	0.0	0	0	Estimated
$M_{\mathrm{a}}(0)$	0.0	0.0	0.0	0.0	0.0	0.0	0	0	Estimated
$P(0)$	30260.5	18901.7	19445.7	11183.2	3818.89	5069.03	1655.51	38545.2	Estimated
$D(0)$	1.40E-02	3.85E-03	5.02E-02	4.54E-02	1.95E-03	3.73E-03	6.76E-04	5.07E-02	Estimated
τ (dying)	1.06159	16.0485	0.994122	0.98037	1.46791	2.4495	0.001	1000	Estimated
τ (proliferating)	4.51897	5.36006	3.34886	2.61633	1.68258	2.36849	0.001	50	Estimated

Supplemental figure S1. ODE models for *L. major* infection. Related to figure 1.

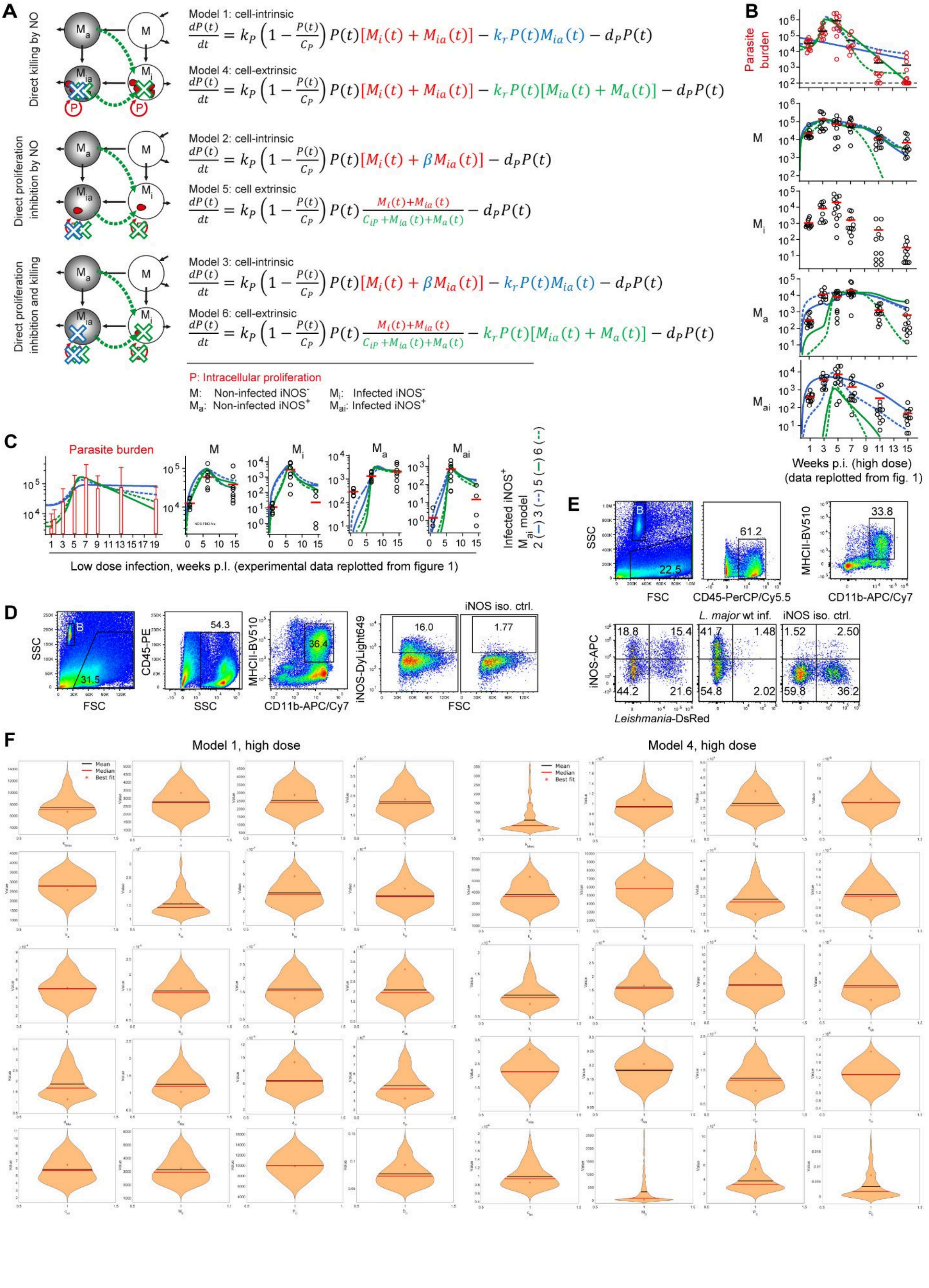

A. Representation of parasite proliferation dependency of recruited cell number (red), cell intrinsic killing and direct growth inhibition (blue) and cell extrinsic killing and growth inhibition (green) in the differential equations for parasite burden over time in the six different models. **B-C.** Overlays of modeled parasite burden and evolution of the different monocyte-derived cell populations over time in model 3 through 6 for high dose (B) and low dose (C) infections. Experimental data (circles and open bars) are replotted from figure 1. **D-E.** Gating examples for determining induction of the immune response (D) in and determination of the M, Ma, Mi, and Mai populations (E). Examples from 5 weeks post infection (wpi) acquired on a LSRFortessa (D) and Attune NxT flow (E) cytometer are shown. B, Gate for counting beads. **F.** 250 different artificial datasets derived from experimental data by sampling all data points from a normal distribution with mean and standard deviation from the original data were used to repeatedly fit model 1 and 4. The fitted parameters are shown as violin plots, including the mean (black line) and median (red line) of each distribution as well as the parameter value of the best fit (asterisk) of the respective model.

Supplemental figure S2. ***In vitro*** **characterization of cell death reporter parasite** ***L. major*****[necR]. Related to figure 2.**

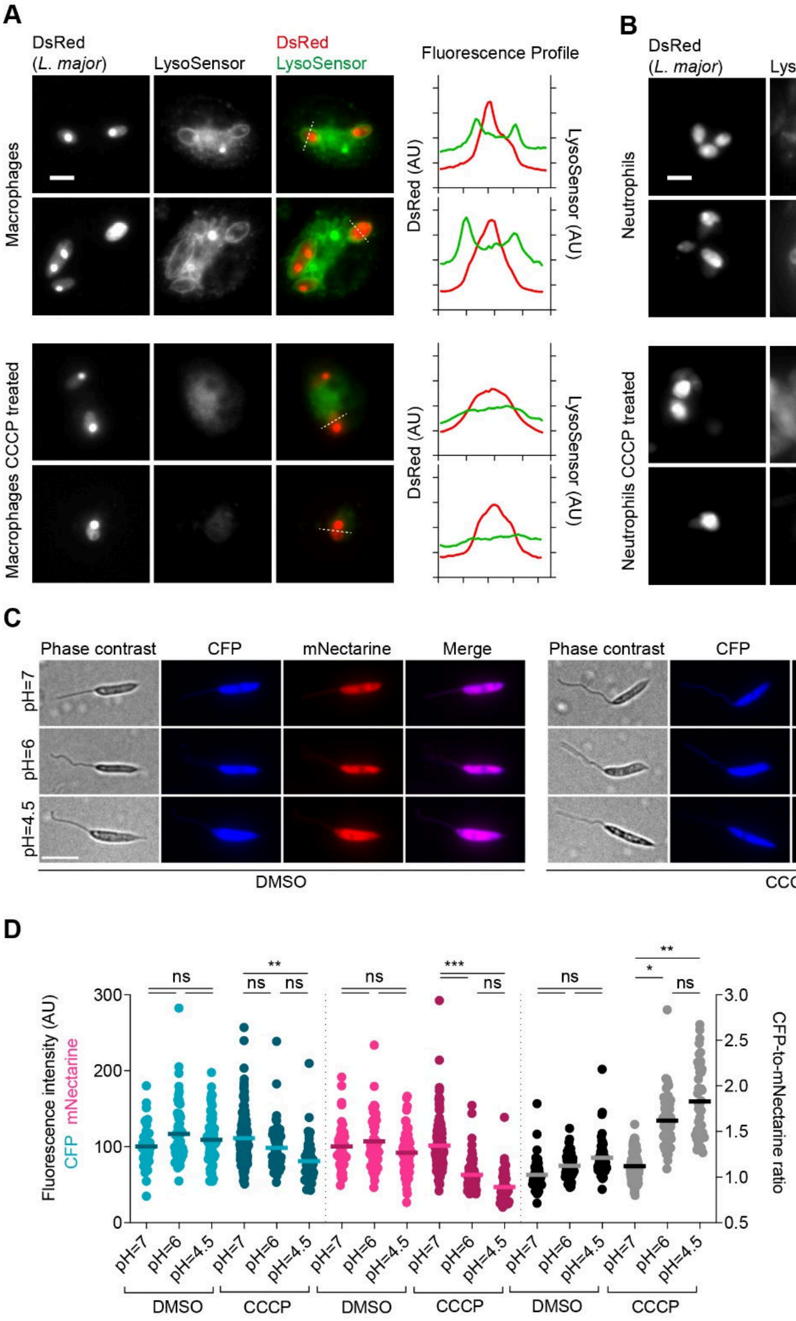

A-B. C57BL/6 peritoneal macrophages (A) and neutrophils purified from mouse bone marrow (B) infected with DsRed-expressing *L. major* (red) stained with LysoSensor Green to highlight acidic compartments within the cells. Upper panels: Two examples of infected phagocytes with a LysoSensor positive compartment surrounding the parasites. Lower panels: Controls treated with the protonophore CCCP which abolishes cellular pH gradients. Fluorescence profiles show parasite (red) and LysoSensor (green) fluorescence intensity along the dotted lines. Scale bar, 5 µm. **C.** Example images of *L. major*^{necR} promastigotes incubated with 0.01% DMSO (control) or 10 µM of the protonophore CCCP in PBS buffered at the indicated pH. Scale bar, 10 µm. **D.** Quantification of CFP and mNectarine fluorescence intensity of individual parasites (left axis) and corresponding CFP-to-Nectarine ratio (right axis) after treatment with CCCP or DMSO at different pH. Horizontal bars denote the mean. ***, p<0.001; **, p>0.01; *, p>0.05; ns, not significant according to one-way ANOVA with Dunn's post-test.

Supplemental figure S3. Analysis of parasite death the ongoing infection. Related to figure 3.

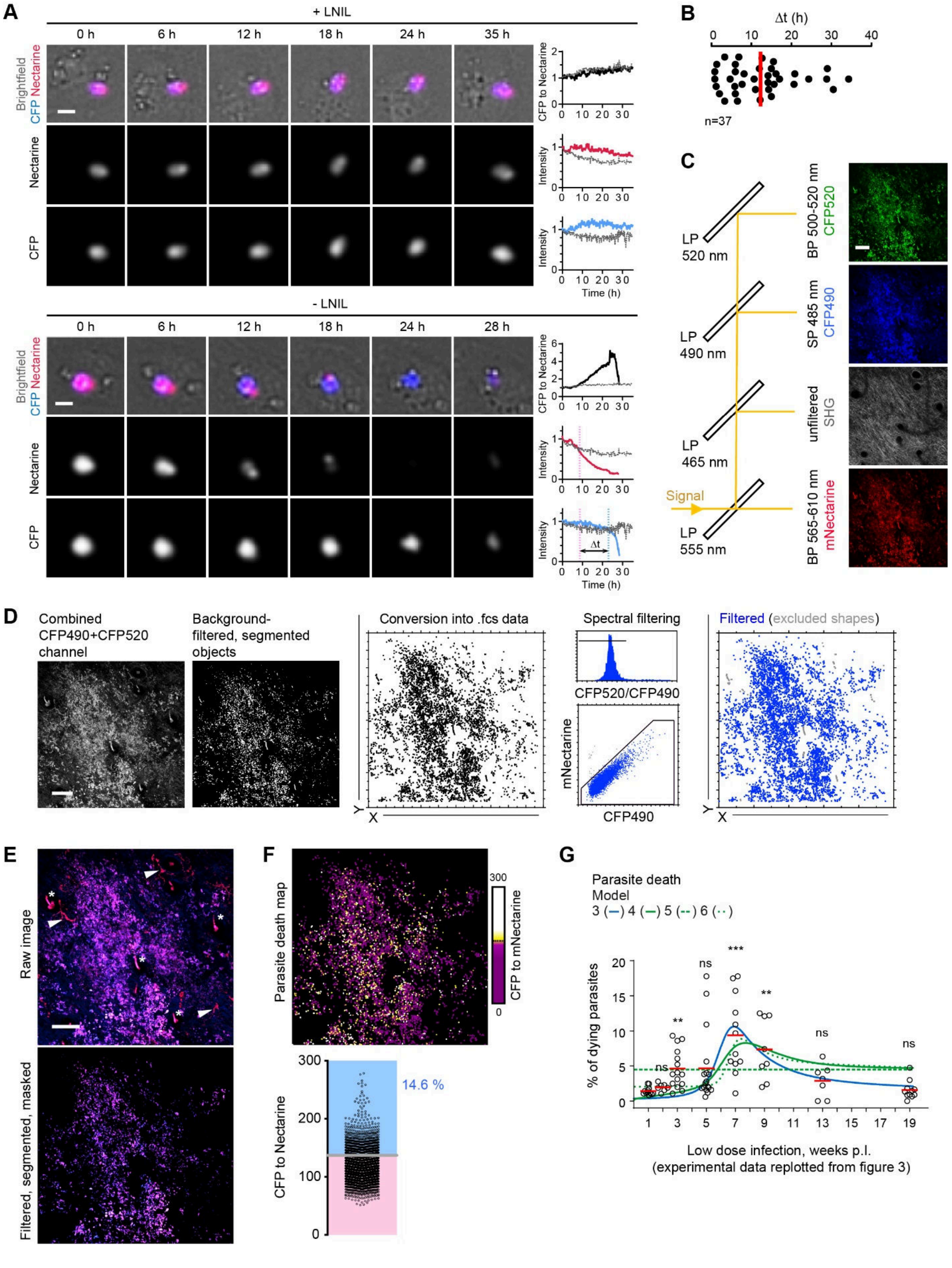

A. Time-lapse microscopy images of IFNγ/LPS activated peritoneal macrophages infected with *L. major*^{necR} with (upper panels) and without (lower panels) inhibition of iNOS-dependent parasite killing by L-NIL treatment. Curves on the right show the quantification over time of Nectarine (red) and CFP (blue) fluorescence intensity as well as corresponding CFP/Nectarine ratio (black). Parasites from LNIL-treated cultures (n=11) were used as controls to determine the 95% confidence interval within which CFP and Nectarine fluorescence values of viable parasites are expected to lie. Grey curves show the lower limit of the confidence interval calculated for Nectarine and CFP fluorescence and the upper limit of the confidence interval determined for CFP/Nectarine ratio. Scale bar, 5 µm. **B.** Time calculated between the significant loss of Nectarine fluorescence, indicating loss of membrane integrity, and loss of CFP fluorescence, indicating parasite disruption. Each dot represents one tracked parasite disappearance event as shown in (A). The vertical bar denotes the mean. **C.** Configuration of the dichroic mirrors and filters used to image ear tissue infected with the *L. major*^{necR} reporter strain. Scale bar, 100 µm. **D.** Representative examples of the combined channel calculated to achieve gradient-based segmentation of the intravital images, XY map representation of the segmented objects after data conversion into flow cytometry data, and spectral filtering strategy used in FlowJo to eliminate autofluorescent objects (hair or cells) associated with high CFP520/CFP490 and mNectarine/CFP490 ratio. Selected objects appear blue, rejected objects are shown in grey. Scale bar, 100 µm. **E.** Representative examples of merged CFP490 and mNectarine channels before (upper panel) or after segmentation and spectral filtering (lower panel). Scale bar, 100 µm. **F.** Upper panel: Representative example of a heat map generated after quantification of parasite viability and corresponding graph. Viable parasites are displayed in purple, dying parasites are shown in yellow or white. Lower panel: representation of the CFP/mNectarine ratio of individual parasites detected within the analyzed site of infection. The grey bar denotes the viability threshold, each symbol represents one parasite. **G.** Overlay of ODE-modelled fractions of dying parasites using models 3 through 6 (see figure 1, supplemental figure S1). Experimental data (circles) are replotted from figure 3).

Supplemental figure S4. Modelling of parasite proliferation in the ongoing infection. Related to figure 4.

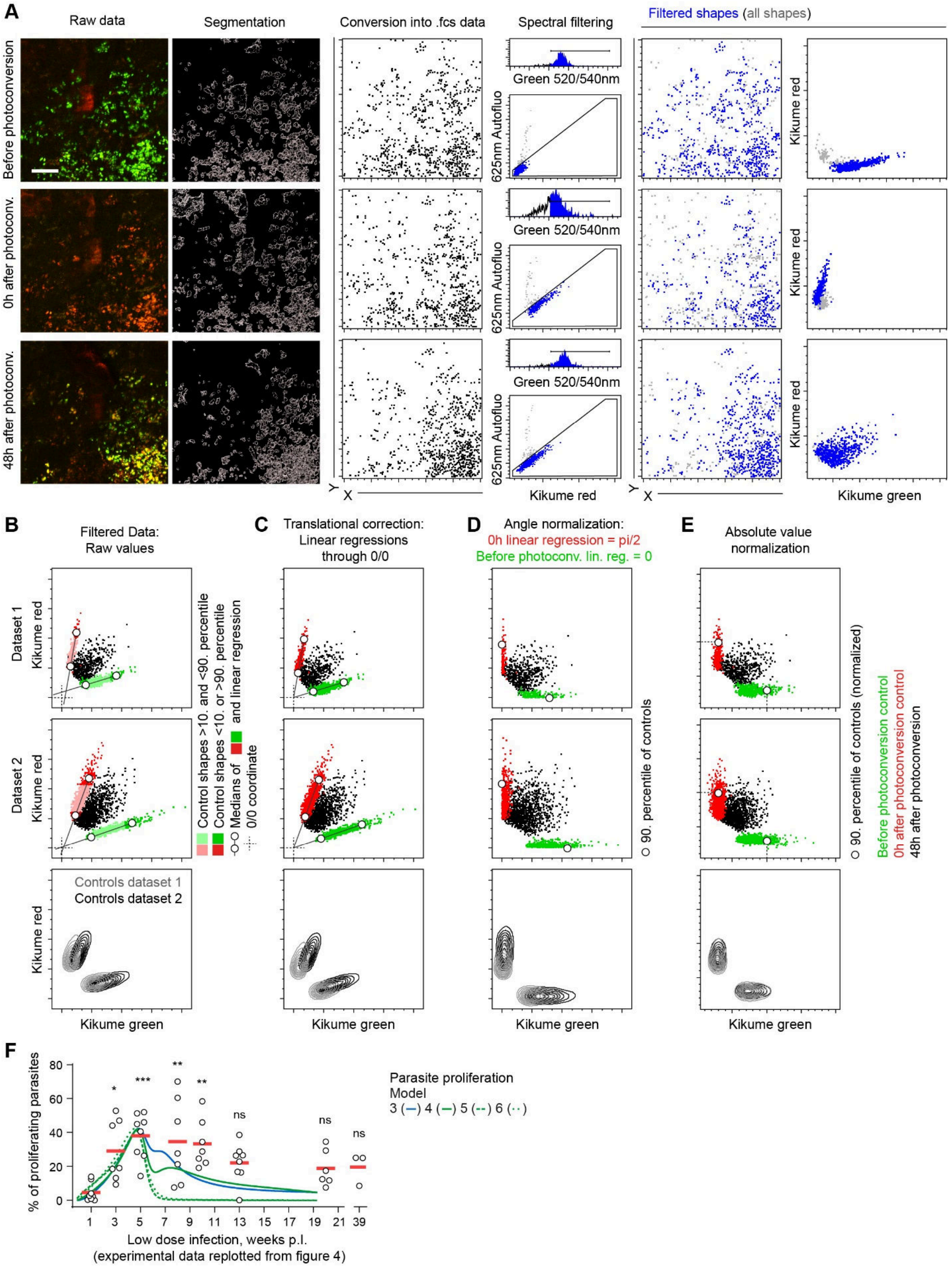

A. Fluorescence data extraction and filtering strategy. An example of data from the same site of imaging before (top), 0 h after photoconversion (middle) and 48h after photoconversion (bottom) is shown. Segmented shapes from the Imaris software are shown, which were converted into flow cytometry data. Spectral filtering was done sequentially, first selecting objects with a high ratio of 520/540 nm emission, and then selecting objects with a high Kikume Red (560-625 nm) to far red autofluorescence (>625 nm) ratio. Positively selected populations are shown in blue. Scale bar, 50 µm. **B.** Determination of slopes of the preconversion (green) and 0 h after photoconversion (red) control populations by determining the median (circles) red and green values of the 10th and 90th percentile in the control populations (percentiles determined according to the Kikume green in the preconversion control, according to Kikume red in the 0h after photoconversion control). The data of two independent examples (top and middle panel) are shown, as well as the overlay of the contour plots of the preconversion and 0h after photoconversion control (bottom panel). **C.** Lateral correction: linear regression curves run through 0/0 (dotted lines). **D.** Angle normalization: linear regression curves of preconversion and 0h after photoconversion controls run at a slope angle of 0 and pi/2, respectively, through the 0/0 coordinate. Circles denote the 90th percentile red fluorescence of the 0h after photoconversion and 90th percentile green fluorescence of the preconversion control. **E.** Normalization of the data to the 90th percentile of the control fluorescences. **F.** Overlay of modelled fractions of proliferating parasites using models 3 through 6 (see figure 1, supplemental figure S1). Experimental data (circles) replotted from figure 4).

Supplemental figure S5. Inhibition of cell recruitment to the site of infection. Related to figure 5.

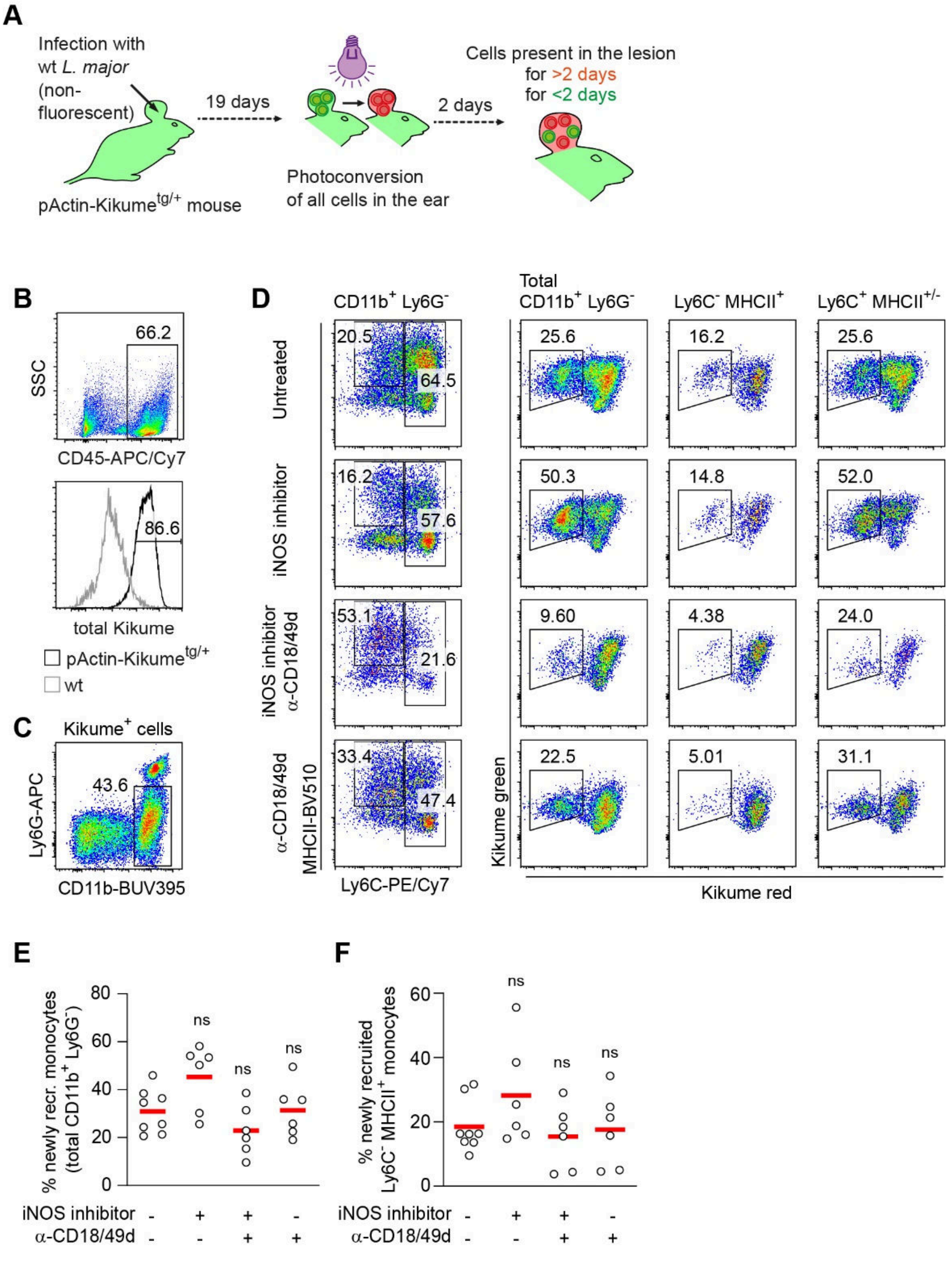

A. Experimental setup for determining the proportion of newly recruited cells in the infected ear. **B-C.** Gating strategy. (B) Kikume-expressing cells were gated from CD45^{+} leukocytes using a calculated total Kikume fluorescence channel. (C) From the resulting Kikume^{+} cells, CD11b^{+}Ly6G^{-} monocyte-derived non-neutrophilic phagocytes were analyzed. **D.** Analysis of recently recruited Ly6C^{+}MHCII$^{+/-}$ and matured Ly6C^{-}MHCII^{+} monocyte-derived non-neutrophilic phagocytes with and without iNOS inhibition by L-NIL and with and without

blocking of phagocyte recruitment. **E-F.** Fraction of total $CD11b^{+}Ly6G^{-}$ (E) and $Ly6C^{-}MHCII^{+}$ cells (F) recruited within the last 2 days before the end of the experiment. Each dot represents an individual ear, data pooled from two independent experiments. Horizontal bars denote the mean; ns, not significant according to one-way ANOVA with Dunnett's post test (comparison with no inhibitor/no antibody control).

Supplemental figure S6. Pathogen burden development under L-NIL with and without blocking of immune cell recruitment. Related to figure 6.

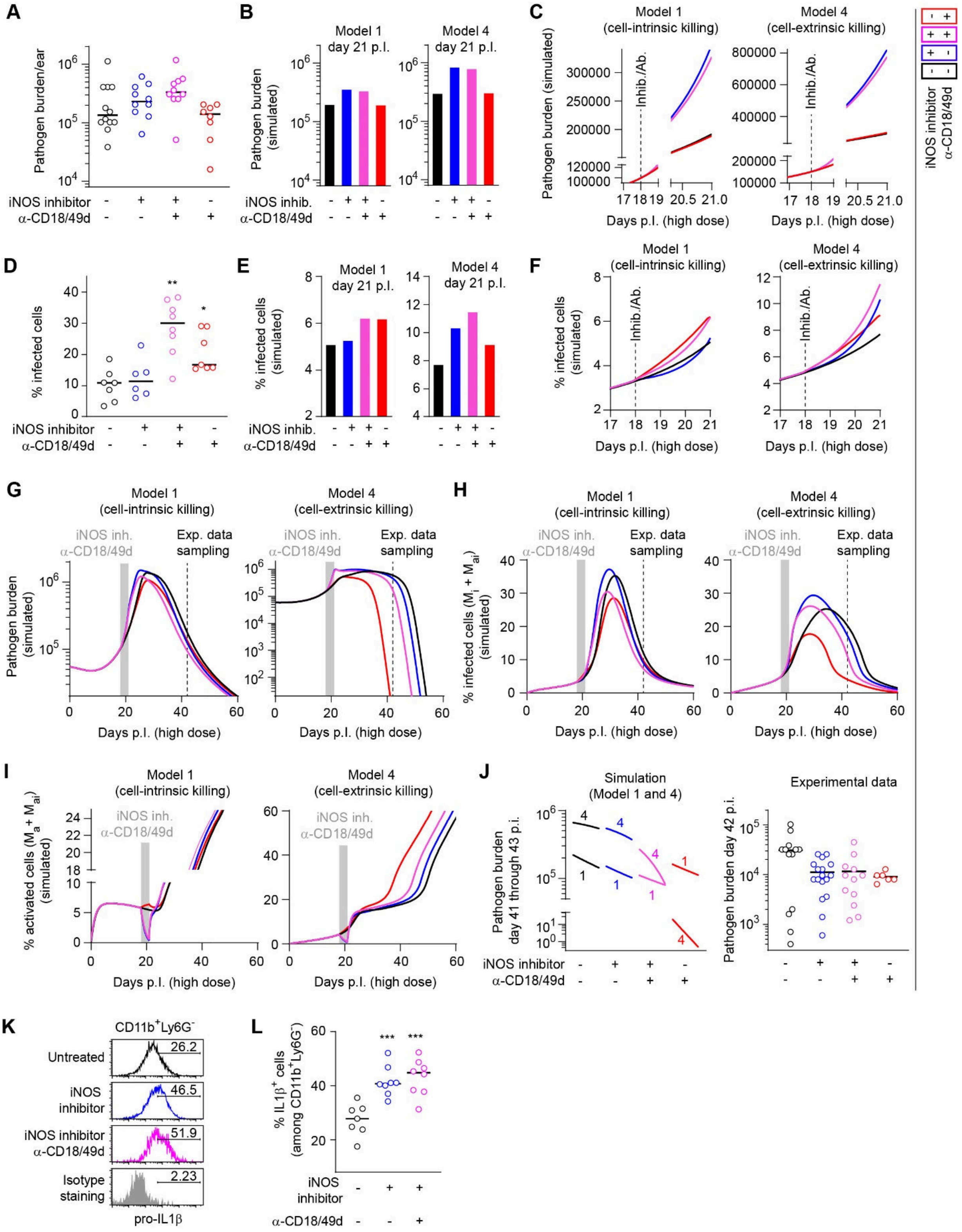

A. Pathogen burden determined experimentally using limiting dilution analysis at 3 wpi (high dose) with and without iNOS inhibition by L-NIL, and/or antibody-mediated blocking of immune cell recruitment from 3 days before analysis. Each symbol represents one infected ear, data are pooled from 4 independent experiments. Horizontal lines denote the median. **B.** Simulated pathogen burden in the ODE model of cell-intrinsic killing (left panel) or cell-extrinsic killing (right panel). Simulated *L. major* numbers shown at day 21 p.i. for untreated (black bars), activation-inhibited (blue bars), recruitment-inhibited (red bars) or a combined inhibition (pink bars) conditions, with treatment initiated at day 18 p.i. **C.** Simulation of pathogen burden in the ODE model of cell-intrinsic (left) or cell-extrinsic (right) killing of *L. major* in untreated (black curves), activation-inhibited (blue curves), recruitment-inhibited (red curves) or combined inhibition (pink curves) for three days, starting from day 18 p.i. **D.** Fraction of infected cells determined by flow cytometry under the conditions as described in (A). Each symbol represents one infected ear, data are pooled from 2 independent experiments. Horizontal lines denote the median. **, $p<0.01$; *, $p<0.05$ according to Kruskal-Wallis test with Dunn's post test (comparison with no inhibitor/no antibody control). **E-F.** Simulated fraction of infected cells in the ODE model as described in (B-C). **G-I.** Simulation of pathogen burden (G), total fraction of infected cells (H), and total fraction of activated cells (I) over 60 days in the ODE model of cell-intrinsic (left panels) or cell-extrinsic (right panels) killing in untreated (black curves), activation-inhibited (blue curves), recruitment-inhibited (red curves) or combined inhibition (pink curves) for three days. The inhibition (grey interval) was modeled to take effect for 3 days, starting from day 18 p.i. A vertical dashed line denotes day 42 p.I., for which time point the modelling predictions were tested experimentally. **J.** Left panel, simulated pathogen burden for model 1 and model 4 in the conditions described under (G-H), curves show the time course from day 41 to day 43. Right panel, pathogen burden determined by limiting dilution analysis at day 42 p.i., after treatment from day 18 p.i. on with three doses of L-NIL (blue), one dose of anti-CD18/CD49d (red), both (pink), or not treated (black). Horizontal bars denote the median. **K-L.** Flow cytometry analysis of pro-IL1β in $CD11b^{+}Ly6G^{-}$ phagocytes isolated from the site of infection of control (black curve and symbols), L-NIL treated (iNOS inhibition, blue curve and symbols) and L-NIL/anti-CD18/49d treated (pink curve and symbols) infected mice. The grey histogram shows an isotype control for the pro-IL1β staining. Each symbol represents one infected ear. Horizontal bars represent the mean. ***, $p<0.001$ according to one-way ANOVA with Dunnett's post test (comparison with no inhibitor/no antibody control).

Supplemental figure S7. Ectopic phagocyte entry in late *L. major* infection. Related to figure 7.

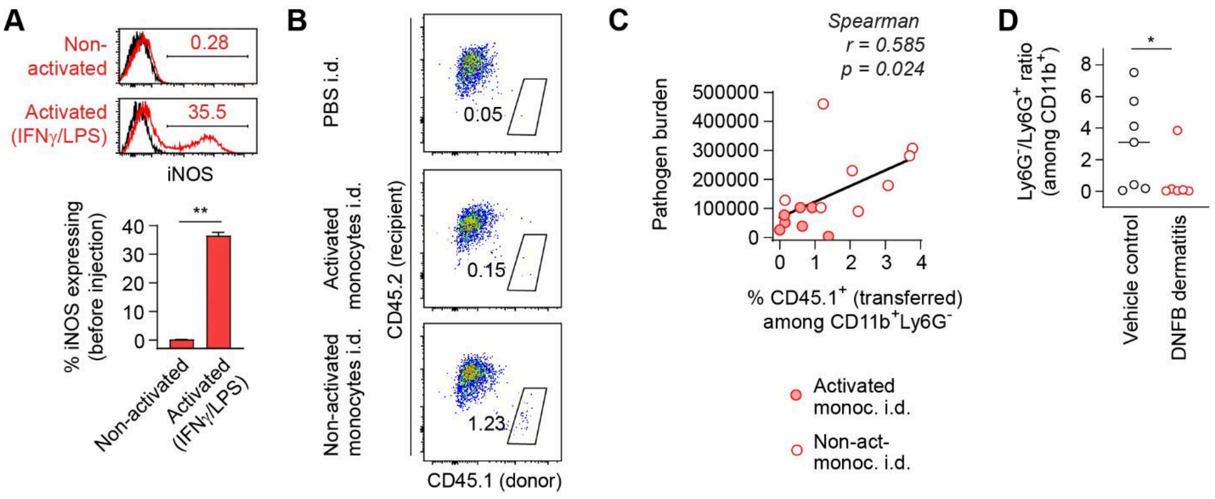

A. Analysis of iNOS expression in bone marrow-isolated monocytes with and without IFNγ/LPS activation prior to injection into 10 wpi infection sites.Red curves; anti-iNOS stained samples; black curves: isotype control staining. Bar diagram shows the mean + standard deviation of 5 replicate analyzed. **, $p<0.01$ according to Mann-Whitney test. **B.** Flow cytometry analysis of CD45.1 (donor) and CD45.2 (recipient) ratios among $CD11b^{+}Ly6G^{-}$ phagocytes isolated from the site of a 10 week infection injected i.d. with PBS (upper panel), activated (middle panel) or non-activated monocytes (lower panel) in the infected ear 3 days before analysis. **C.** Correlation between the pathogen burden and fraction of monocytes detected 3 days after i.d. deposition into the site of injection. **D.** Ratio of $Ly6G^{-}$ to $Ly6G^{+}$ cells among $CD11b^{+}$ phagocytes isolated from a 10 week infection site treated with vehicle solution (black symbols) or DNFB in order to induce contact dermatitis three days before analysis. Each symbol represents one individual infected ear. Data pooled from two independent experiments. The median is shown by a horizontal line. *, $p<0.05$ according to Mann-Whitney test.
