# Supplementary material for: Nitric oxide controls proliferation of Leishmania major by inhibiting the recruitment of permissive host cells
Source: Immunity. 2021 Dec 14;54(12):2724–2739.e10. doi: 10.1016/j.immuni.2021.09.021 (PMC8691385; doi:10.1016/j.immuni.2021.09.021)
Supplement: Document S2. Article plus supplemental information [file mmc2.pdf]

Immunity

Nitric oxide controls proliferation of *Leishmania major* by inhibiting the recruitment of permissive host cells

Graphical abstract

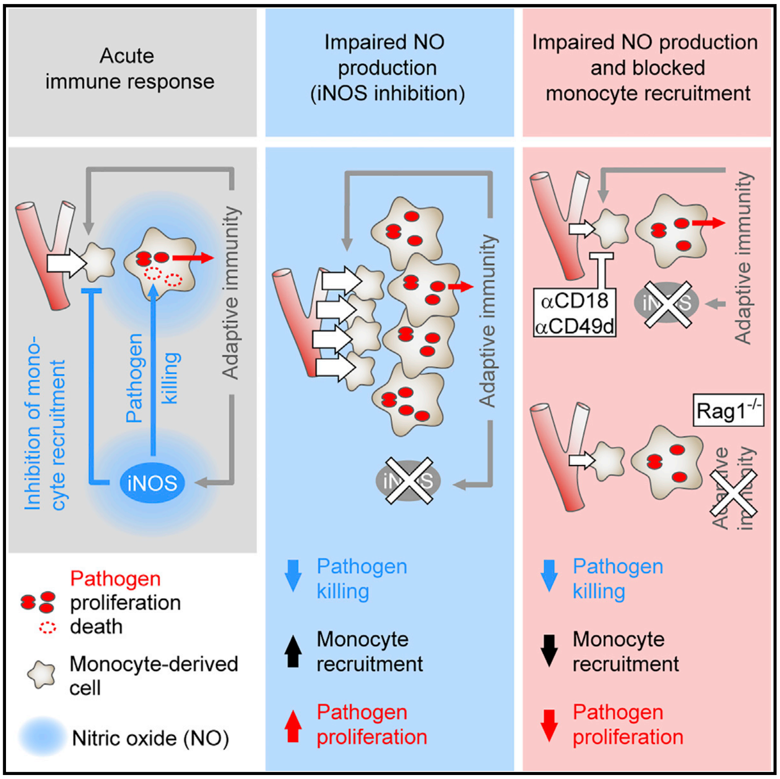

Highlights

- Direct killing of *L. major* by NO occurs only during the peak of the immune response
- Efficient *L. major* proliferation requires newly recruited monocyte-derived cells
- Loss of NO production increases both pathogen proliferation and monocyte recruitment
- NO dampens *L. major* proliferation indirectly, limiting the pathogen's cellular niche

Authors

Pauline Formaglio,
Mohamad Alabdullah,
Anastasios Siokis, ...,
Sebastian Binder,
Michael Meyer-Hermann,
Andreas J. Müller

Correspondence

pauline.formaglio@pasteur.fr (P.F.),
andreas.mueller@med.ovgu.de (A.J.M.)

In brief

Besides direct antimicrobial activity, nitric oxide (NO) can inhibit the entry of inflammatory cells into infected tissues. Intracellular pathogens can hijack such cells as niches for proliferation. Formaglio et al. show that restriction of proliferation-permissive host cell recruitment by NO represents a mechanism that controls *Leishmania major* infection.

Formaglio et al., 2021, Immunity *54*, 2724–2739
December 14, 2021 © 2021 The Author(s). Published by Elsevier Inc.
https://doi.org/10.1016/j.immuni.2021.09.021

CellPress
OPEN ACCESS

Immunity

Article

Nitric oxide controls proliferation of *Leishmania major* by inhibiting the recruitment of permissive host cells

Pauline Formaglio,[1,11,*] Mohamad Alabdullah,[1,11] Anastasios Siokis,[2] Juliane Handschuh,[1] Ina Sauerland,[1] Yan Fu,[1] Anna Krone,[1] Patricia Gintschel,[1] Juliane Stettin,[1] Sandrina Heyde,[1] Juliane Mohr,[1] Lars Philipsen,[1] Anja Schröder,[3] Philippe A. Robert,[2,4] Gang Zhao,[2] Sahamoddin Khailaie,[2] Anne Dudeck,[1] Jessica Bertrand,[3] Gerald F. Späth,[5] Sascha Kahlfuß,[1] Philippe Bousso,[6] Burkhart Schraven,[1] Jochen Huehn,[7,8] Sebastian Binder,[2] Michael Meyer-Hermann,[2,9] and Andreas J. Müller[1,10,12,*]
[1]Institute of Molecular and Clinical Immunology, Health Campus Immunology Infectiology and Inflammation (GC-I[3]), Otto-von-Guericke-University, Magdeburg 39120, Germany
[2]Department of Systems Immunology and Braunschweig Integrated Centre of Systems Biology, Helmholtz Centre for Infection Research, Braunschweig 38124, Germany
[3]Experimental Orthopedics, Health Campus Immunology Infectiology and Inflammation (GC-I[3]), Otto von Guericke University, Magdeburg 39120, Germany
[4]Department of Immunology, University of Oslo, Oslo 0372, Norway
[5]Molecular Parasitology and Signalling Unit, Institut Pasteur, Paris 75015, France
[6]Dynamics of Immune Responses Unit, Institut Pasteur, INSERM U1223, Paris 75015, France
[7]Department Experimental Immunology, Helmholtz Centre for Infection Research, 38124 Braunschweig, Germany
[8]Cluster of Excellence RESIST (EXC 2155), Hannover Medical School, Hannover 30625, Germany
[9]Institute of Biochemistry, Biotechnology and Bioinformatics, Technische Universität Braunschweig, Braunschweig 38106, Germany
[10]Intravital Microscopy of Infection and Immunity, Helmholtz Centre for Infection Research, Braunschweig 38124, Germany
[11]These authors contributed equally
[12]Lead contact
*Correspondence: pauline.formaglio@pasteur.fr (P.F.), andreas.mueller@med.ovgu.de (A.J.M.)
https://doi.org/10.1016/j.immuni.2021.09.021

SUMMARY

Nitric oxide (NO) is an important antimicrobial effector but also prevents unnecessary tissue damage by shutting down the recruitment of monocyte-derived phagocytes. Intracellular pathogens such as *Leishmania major* can hijack these cells as a niche for replication. Thus, NO might exert containment by restricting the availability of the cellular niche required for efficient pathogen proliferation. However, such indirect modes of action remain to be established. By combining mathematical modeling with intravital 2-photon biosensors of pathogen viability and proliferation, we show that low *L. major* proliferation results not from direct NO impact on the pathogen but from reduced availability of proliferation-permissive host cells. Although inhibiting NO production increases recruitment of these cells, and thus pathogen proliferation, blocking cell recruitment uncouples the NO effect from pathogen proliferation. Therefore, NO fulfills two distinct functions for *L. major* containment: permitting direct killing and restricting the supply of proliferation-permissive host cells.

INTRODUCTION

Inflammatory reactions involve the massive recruitment of immune cells that deploy cellular defenses at sites of infection but also have to be tightly controlled to avoid unnecessary immunopathology (Ortega-Gómez et al., 2013; Sugimoto et al., 2016). Besides its well-established role as an antimicrobial effector mechanism (Liew et al., 1990; MacMicking et al., 1997; Olekhnovitch et al., 2014), nitric oxide (NO), which is produced by inducible NO synthase (iNOS), provides critical immunomodulatory feedback. Through metabolic reprogramming of monocyte-derived cells at the site of infection and blunting of cytokine production (Postat et al., 2018), NO dampens the recruitment of inflammatory cells such as monocyte-derived inflammatory phagocytes (called monocyte-derived cells hereafter) and neutrophils (Bogdan, 2015; Chandrasekar et al., 2013; Kobayashi, 2010; Mishra et al., 2013; Norman et al., 2008).

Although restricting the entry of inflammatory cells is of utmost importance for limiting tissue damage to the host, intracellular pathogens such as *Toxoplasma*, *Mycobacterium*, or *Leishmania* spp. depend on cell recruitment for efficient establishment of infection (Abidin et al., 2017; Grainger et al., 2013; Heyde et al., 2018; Mishra et al., 2017; Terrazas et al., 2017). For example, the protozoan parasite *Leishmania major* exhibits its highest proliferation rates in monocyte-derived cells newly

 © 2021 The Author(s). Published by Elsevier Inc.
This is an open access article under the CC BY license (http://creativecommons.org/licenses/by/4.0/).

CellPress
OPEN ACCESS

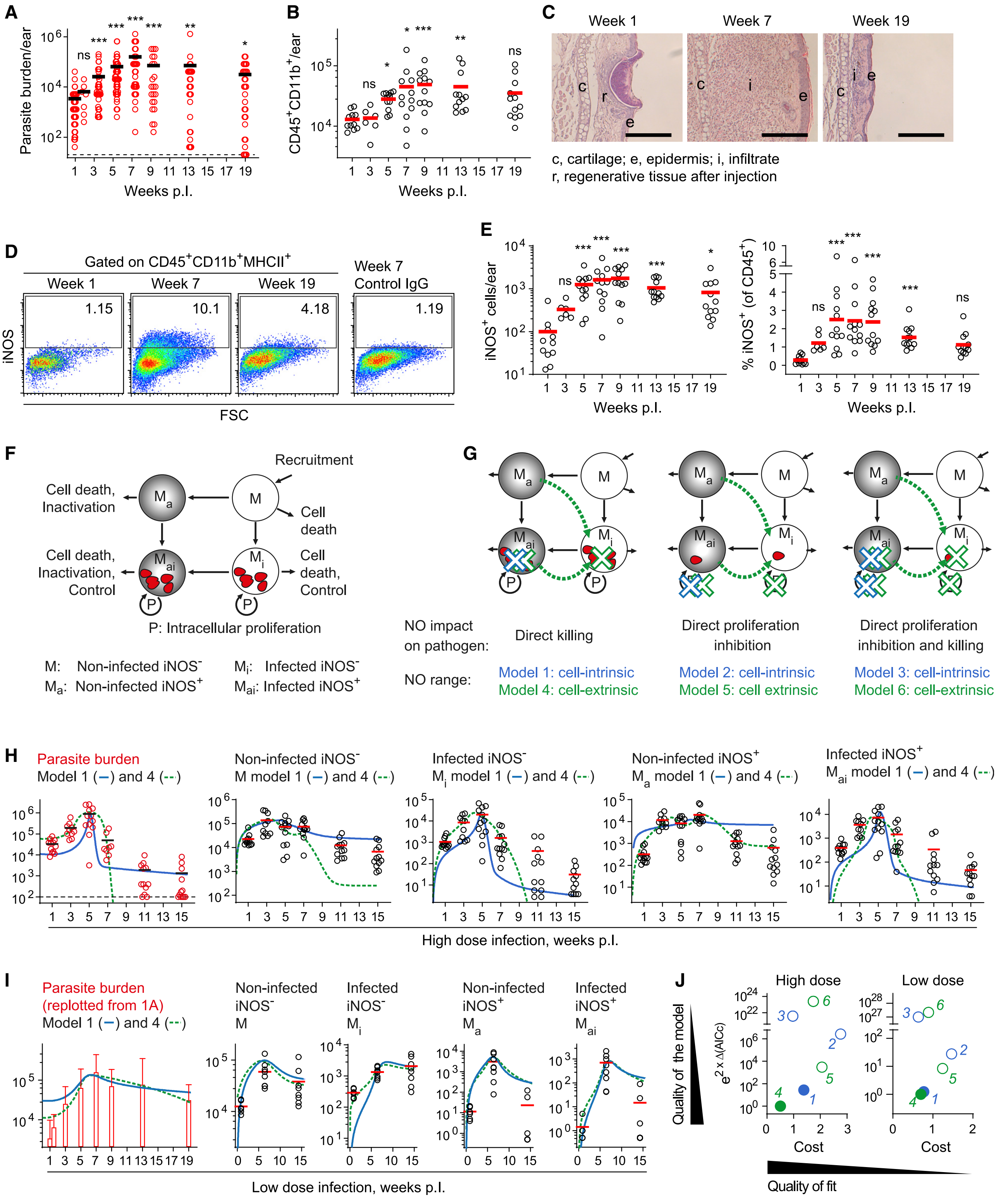

Figure 1. Ordinary differential equation-based modeling of *L. major* high- and low-dose infection

(A–E) Time course of low-dose (5×10^3 metacyclics) *L. major* infection analyzed by limiting dilution (A), flow cytometry (B), hematoxylin and eosin staining (C), and intracellular flow cytometry staining of iNOS (D and E). ***p < 0.001; **p < 0.01; *p < 0.05; ns, not significant, according to Kruskal-Wallis with Dunn's post-test (comparison with 1 wpi data). Scale bar, 200 μm.

(legend continued on next page)

recruited to the site of infection (Heyde et al., 2018; Romano et al., 2017). Therefore, besides direct antimicrobial activity, NO might restrict the availability of the cellular niche required for efficient pathogen proliferation. Furthermore, NO-dependent metabolic reprogramming of monocyte-derived cells could affect their permissiveness for intracellular pathogen proliferation (Postat et al., 2018).

To address to what extent intracellular pathogens are controlled by such indirect modes of action, we employed infection with *L. major* as a physiological model of inflammation involving massive recruitment of monocyte-derived cells in the skin (Postat et al., 2018; Romano et al., 2017; Scorza et al., 2017). These cells not only constitute the main infected cellular compartment but also express iNOS in response to T cell-derived interferon-γ (IFNγ) (De Trez et al., 2009; León et al., 2007; Müller et al., 2012; Olekhnovitch et al., 2014; Romano et al., 2017). NO is critical for pathogen control, which ensues in most immunocompetent individuals (Belkaid et al., 2000; Okwor and Uzonna, 2008; Scott and Novais, 2016). However, a small number of parasites remain at the site of infection without overt pathology, which is critical for immunity against secondary infections (Belkaid et al., 2001, 2002). Even in late phases of persistent infection, control of *L. major* relies on iNOS (Mandell and Beverley, 2017; Stenger et al., 1996). *In vitro*, high concentrations of NO kill *L. major* (Olekhnovitch et al., 2014), but in the infected tissue, viable parasites can be observed in iNOS-expressing cells (Mandell and Beverley, 2017). Furthermore, NO might reduce pathogen proliferation without killing the pathogen (Kloehn et al., 2015; Lemesre et al., 1997; Müller et al., 2013). How the various ways in which NO affects *L. major* physiology and the inflammatory microenvironment contributes to pathogen containment have not yet been fully elucidated.

Here, by employing biosensors for *in vivo* quantification of *L. major* death and proliferation, we found both overt pathogen killing and high pathogen proliferation at the peak of the immune response and reduced pathogen proliferation in the absence of killing during the chronic phase. Low pathogen proliferation resulted from reduced availability of permissive host cells at the site of infection, whereas the increase in pathogen proliferation upon iNOS inhibition was reverted by blocking extravasation of immune cells from the bloodstream. *In situ* deposition of monocytes, but not recruitment of neutrophils, increased pathogen burden during late stages of the infection. Thus, besides a direct impact on pathogen viability, mainly during the peak of infection, NO contributes to lasting control of infection by inhibiting the recruitment of cellular compartments in which *L. major* can proliferate.

RESULTS

Availability of permissive host cells and pathogen killing are the main determinants of the *L. major* infection course in mathematical models

To dissect direct killing of intracellular pathogen from non-lethal modes of action of NO, we used a model of intradermal *L. major* ear skin infection (Heyde et al., 2018; Müller et al., 2012, 2013; Peters et al., 2008; Romano et al., 2017), in which the restriction of inflammatory cell recruitment by NO is well established (Postat et al., 2018). The ear pinnae of C57BL/6 wild-type (WT) mice were inoculated with 5×10^3 *L. major* metacyclics and analyzed by limiting dilution analysis over the course of the infection (Figure 1A). In agreement with previous work (Belkaid et al., 2002), parasite burden gradually increased, reached its maximum at 7 weeks post-infection (wpi), and eventually decreased by about 90% but was still detectable at 19 wpi. Similarly, neutrophils and monocyte-derived cells, as reflected by the number of $CD45^+$ $CD11b^+$ cells present at the site of inoculation, progressively expanded until 9 wpi and contracted at later time points (Figure 1B). Histological examination of the tissue mirrored this observation, showing substantial immune infiltration at the peak of infection, whereas no overt pathology was detected by 19 wpi (Figure 1C).

$CD11b^+MHCII^+$ cells are a main iNOS-expressing monocyte-derived cell population during *L. major* infection (De Trez et al., 2009). The total number of iNOS-expressing $CD11b^+MHCII^+$ cells was highest between 7 and 9 wpi but remained elevated after this peak compared with before the onset of the adaptive immune response (Figures 1D and 1E). Thus, an initial phase of *L. major* infection is characterized by high parasite load and massive immune cell infiltration, and a later phase is associated with low parasite burden and low, but continuous, phagocyte activation.

Pathogen killing and direct inhibition of proliferation might act not only cell-intrinsically but also via diffusion across the tissue (Olekhnovitch et al., 2014). To systematically analyze which modes of action could explain best the observed course of pathogen control, we set out to model the temporal evolution of pathogen burden, infection, and activation of monocyte-derived cells using ordinary differential equations (ODEs) . In brief, ODE systems (Figures 1F and S1A) were set up in which non-activated monocyte-derived cells (M) are recruited to the site of infection and can become activated as a result of pathogen burden and thus express iNOS (M_a), become infected (M_i), or both via sequential activation or infection in either order (M_{ai}). In three of these ODE systems, iNOS mediates direct killing (model 1),

(F) Ordinary differential equation model of monocyte-derived cells according to their activation (iNOS expression) and infection state. Arrows: possible state transitions. M, non-activated, non-infected cells; M_a, activated (iNOS expressing); M_i, infected; M_{ai}, activated and infected; P, parasite intracellular proliferation. Gray shading: activation (iNOS expression), red: parasites.

(G) *L. major* containment in the different models. Blue: cell-intrinsic killing and proliferation inhibition by activated, infected cells (models 1–3). Green: cell-extrinsic killing and proliferation inhibition within all infected cells exerted by all activated cells (models 4–6).

(H and I) High-dose (2×10^6 stationary parasites, H) and low-dose (5×10^3 purified metacyclic parasites, I) infection data used to inform the models. Each symbol in (A), (B), (E), (H), and (I) represents one infected ear. Horizontal bars denote the mean. Data cumulated from at least two independent experiments. Parasite burden data in (I) replotted from (A) as mean + standard deviation. Overlays of best fits for model 1 (solid lines) and model 4 (dashed lines) are shown.

(J) Costs of fitting plotted against quality of the model determined as a function of the AICc (corrected Akaike information criterion). Low-cost, high-quality models appear in the lower left corner of the graphs. The AICc of the model with the lowest AICc (model 4) was set to 0. The models that cannot be excluded by $e^{(2 \times \Delta AICc)}$ are represented by filled circles; all other models are represented by empty circles.

See also Figure S1.

CellPress
OPEN ACCESS

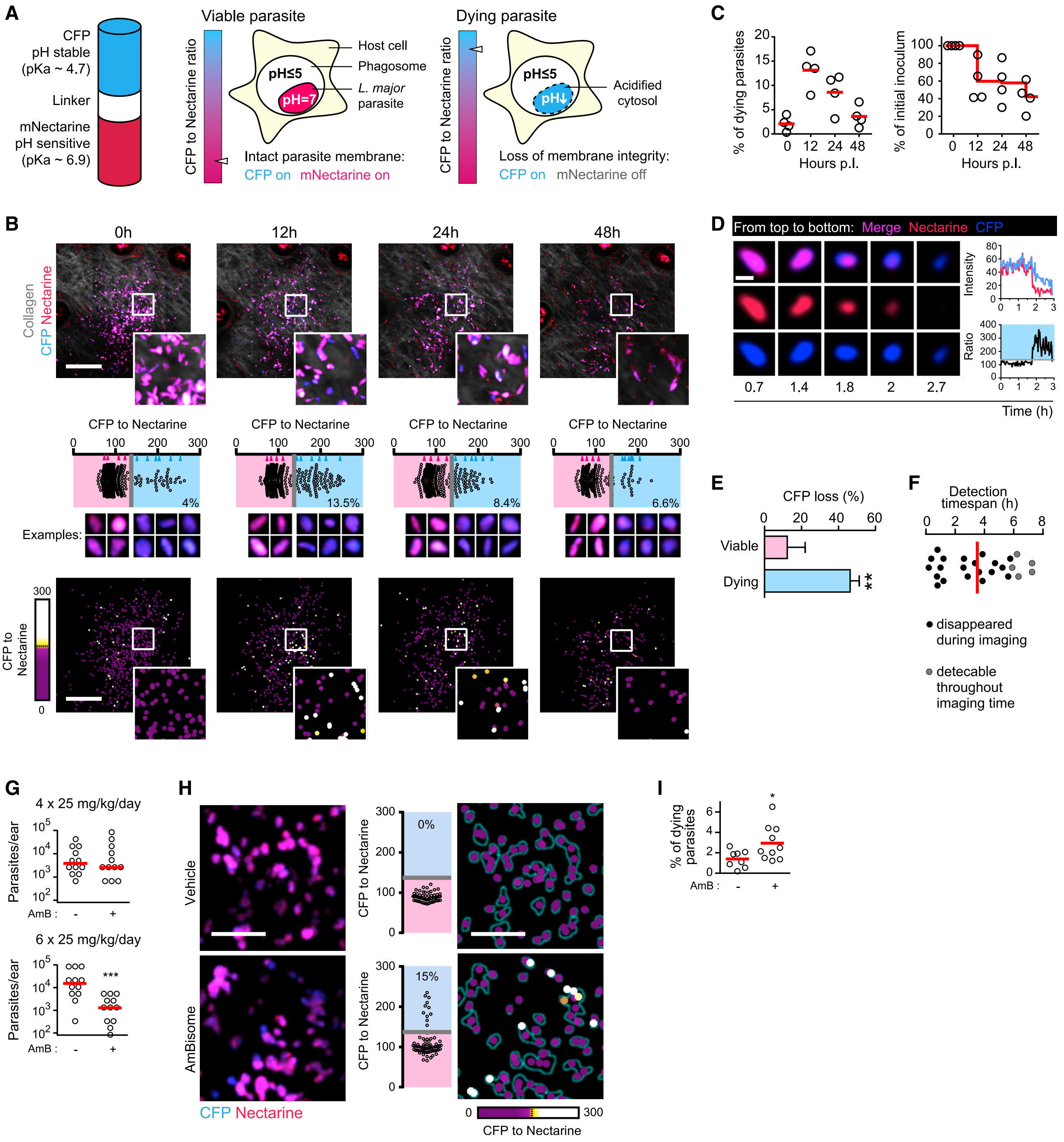

Figure 2. Establishment of a pH-based death reporter parasite *L. major*^{necR}

(A) Reporter construct expressed within *L. major*^{necR}. In a viable parasite, both CFP (blue) and pH-sensitive mNectarine (red) exhibit fluorescence, because the pH is neutral within the parasite. In a dying parasite, the parasite cytosol is acidified by the phagosomal environment (≤pH 5), and mNectarine fluorescence is lost. (B–F) IV-2PM of mice infected with stationary-phase promastigote *L. major*^{necR}. (B) Upper row: parasites (red: mNectarine, CFP: blue) and collagen second harmonic (SH) signal (gray) imaged at the indicated time points p.i. Middle row: CFP-to-mNectarine ratio and examples of segmented parasites in the respective images. The parasites shown are marked as wedges in the graphs. Bottom row: heatmaps of the CFP-to-mNectarine ratio. Maximum-intensity projections of at least 17 z planes spaced 3 μm apart are shown. Scale bar, 100 μm. (C) Fraction of dying parasites detected by IV-2PM in early infections with stationary-phase promastigotes (left graph) and number of parasites detected by IV-2PM (right graph). Each circle represents one infection site imaged longitudinally; lines denote the mean. (D) IV-2PM example of a parasite losing mNectarine fluorescence before losing CFP fluorescence. Note the sharp increase in the CFP (blue curve) to mNectarine (red curve) fluorescence ratio (black curve). Scale bar, 5 μm. (E) Comparison of loss of CFP fluorescence (% initial fluorescence) in parasites exhibiting mNectarine fluorescence loss (n = 25; see E, blue bar) with parasites that retained mNectarine fluorescence throughout the imaging experiment (n = 13, pink bar). Mean values + standard deviations are shown. **$p < 0.01$, according to Mann-Whitney test. (F) Detection time span of dying parasites (n = 25) tracked from 11 h

(legend continued on next page)

direct inhibition of *L. major* growth (model 2), or a combination of both (model 3) through the action of only iNOS-expressing infected cells, controlling the pathogen cell-intrinsically within activated, infected cells (Figures 1G and S1A, blue symbols). Three additional ODE systems in which a direct effect—killing (model 4), growth inhibition (model 5), or both (model 6)—is exerted by all iNOS-expressing cells on the pathogens in all infected cells (both cell extrinsic and cell intrinsic) were set up (Figures 1G and S1A, green symbols). In all models, pathogen proliferation depended on the availability of infectable host cells (Figures 1G and S1A, red symbols), because *L. major* is assumed to be obligate intracellular in the mammalian host for efficient proliferation (Abidin et al., 2017; Heyde et al., 2018; Terrazas et al., 2017; Walker et al., 2014).

We then estimated the model parameters by numerical optimization (Robert et al., 2018) based on experimental data (Figures 1A, 1H, and 1I) on *L. major* burden of, infection of, and iNOS expression by monocyte-derived cells. Because the course of pathogen burden and immune reaction have been shown to evolve at different speeds depending on the initial infection dose, we used high-dose (2×10^6 stationary parasites) and low-dose (5×10^3 purified metacyclic parasites) infection data as two separate datasets to assess the performance of each model (Figures 1H, 1I, and S1B–S1E).

To select the best model for the observed data, the corrected Akaike information criterion (AICc) (Zhao et al., 2017) was calculated (Figure 1J). Bootstrapping confirmed that the range of parameter values was constrained by the experimental data and did not detect relevant correlation between parameters; therefore, each parameter could be identified independently (Figure S1F; Tables S1 and S2). In the high- and the low-dose settings, models 1 and 4 explained best the course of events observed in the experiments, regarding both the quality of the model, determined by low AICc, and the low cost of fitting (Figure 1J). Altogether, the mathematical modeling predicted that direct killing of *L. major*, but not direct growth inhibition by iNOS, is a dominant mechanism of pathogen control.

Overt pathogen killing occurs under high immune activation, but not during persistent infection

iNOS inhibition increases pathogen burden not only during early phases with high immune activation but also during the persistence phase of *L. major* infection (Müller et al., 2013; Stenger et al., 1996). This drove us to investigate pathogen killing with a reporter system for *L. major* death within phagocytes *in vivo*: Because intracellular *L. major* resides inside acidic phagolysosomes (Figures S2A and S2B) but can maintain its intracellular pH as neutral (Antoine et al., 1998), we hypothesized that membrane integrity loss would result in a decrease of pH within dying parasites. To monitor *L. major* cytosolic pH, a pH-stable blue fluorescent protein (CFP, ~4.0 pK_A) (Breart et al., 2008) fused to a pH-sensitive red fluorescent protein (mNectarine, ~6.9 pK_A) (Johnson et al., 2009) was constitutively expressed in the parasite. Viable intracellular parasites expressing the reporter system should exhibit fluorescence from both blue and red protein and show a low CFP-to-mNectarine ratio. In contrast, dying, acidified parasites should display blue fluorescence from CFP only and be identifiable by a high CFP-to-mNectarine ratio (Figure 2A). This newly generated *L. major*^{necR} death reporter strain responded to low pH in the presence of the protonophore carbonylcyanid-3-chlorphenylhydrazon (CCCP) *in vitro* with an increased CFP-to-mNectarine ratio (Figures S2C and S2D), as did *L. major*^{necR} parasites within macrophages activated with IFNγ/lipopolysaccharide (LPS) to produce NO (Figure S3A). Decreased mNectarine fluorescence with a stable CFP signal lasted for an average of 12.3 h (Figure S3B). To confirm these findings *in vivo*, WT mice were infected with stationary-phase *L. major*^{necR} promastigotes and subjected to intravital 2-photon microscopy (IV-2PM) at different time points up to 48 h post-infection (p.i.) in the living tissue. Unless specifically enriched, stationary-phase cultures consist mainly of non-metacyclic forms, which die rapidly upon uptake by phagocytes (Späth and Beverley, 2001) and should thus display swift activation of the biosensor. By segmentation of the parasites acquired by IV-2PM (Figures S3C–S3F) and *in situ* cytometry (Moreau et al., 2012), we determined their CFP-to-mNectarine ratio (Figure 2B; STAR Methods). Immediately after inoculation, most parasites are expected to be extracellular and should appear viable with a low CFP-to-mNectarine ratio. A CFP-to-mNectarine threshold value detecting at least 95% of the promastigote population at 0 h p.i. as alive was determined (Figures 2B and 2C). Visual inspection *a posteriori* confirmed that the established cutoff defined two distinct parasite populations displaying an evident shift in color from magenta to blue (Figure 2B, middle panel) and could be applied to calculate the fraction of dying parasites at subsequent time points. The proportion of parasites lacking a red signal and displaying predominantly CFP fluorescence peaked at 12 h p.i. and gradually returned to basal levels by 48 h p.i. By this time point, parasite numbers had decreased by more than 50%, suggesting that the parasites with an elevated CFP-to-mNectarine ratio at 12 h p.i. represented the fraction of dying parasites (Figures 2B and 2C). Time-lapse imaging performed at the peak of promastigote death revealed loss of mNectarine fluorescence followed by gradual fading of the CFP content, culminating with parasite disappearance (Figure 2D). Of 25 tracked parasites that exhibited cytosol acidification at the beginning (n = 16) or within (n = 9) the course of imaging, 20 were eventually cleared. In addition, all parasites that had switched color subsequently displayed a loss of CFP signal, in contrast to parasites that retained mNectarine fluorescence

p.i. on which exhibited mNectarine loss either at the beginning (n = 16) or within the course (n = 9) of an 8-h 2-photon time-lapse acquisition *in vivo*. Black-filled circles: parasites that disappeared during imaging, gray-filled circles: parasites still detectable at the end of the imaging. Vertical bar: mean.
(G–I) Low-dose infected mice (5×10^3 metacyclic *L. major*^{necR} per ear) were treated from day 15 p.i. onward with AmBisome or vehicle solution. Pathogen burden (G) and pathogen death assessed by IV-2PM of *L. major*^{necR} (H and I). (H) Example images (left), quantification (middle), and heatmap of parasite death (right) of vehicle-treated (upper row) and AmBisome-treated mice after 5 consecutive days of treatment. Maximum-intensity projections of at least 22 z planes spaced 3 μm apart are shown. Scale bar, 20 μm. (I) Fraction of dying parasites determined by IV-2PM of *L. major*^{necR} after 5 days of treatment. Each symbol represents one individual ear. Data pooled from two independent experiments. ***$p < 0.001$; *$p < 0.05$, according to Mann-Whitney test.
See also Figure S2.

CellPress
OPEN ACCESS

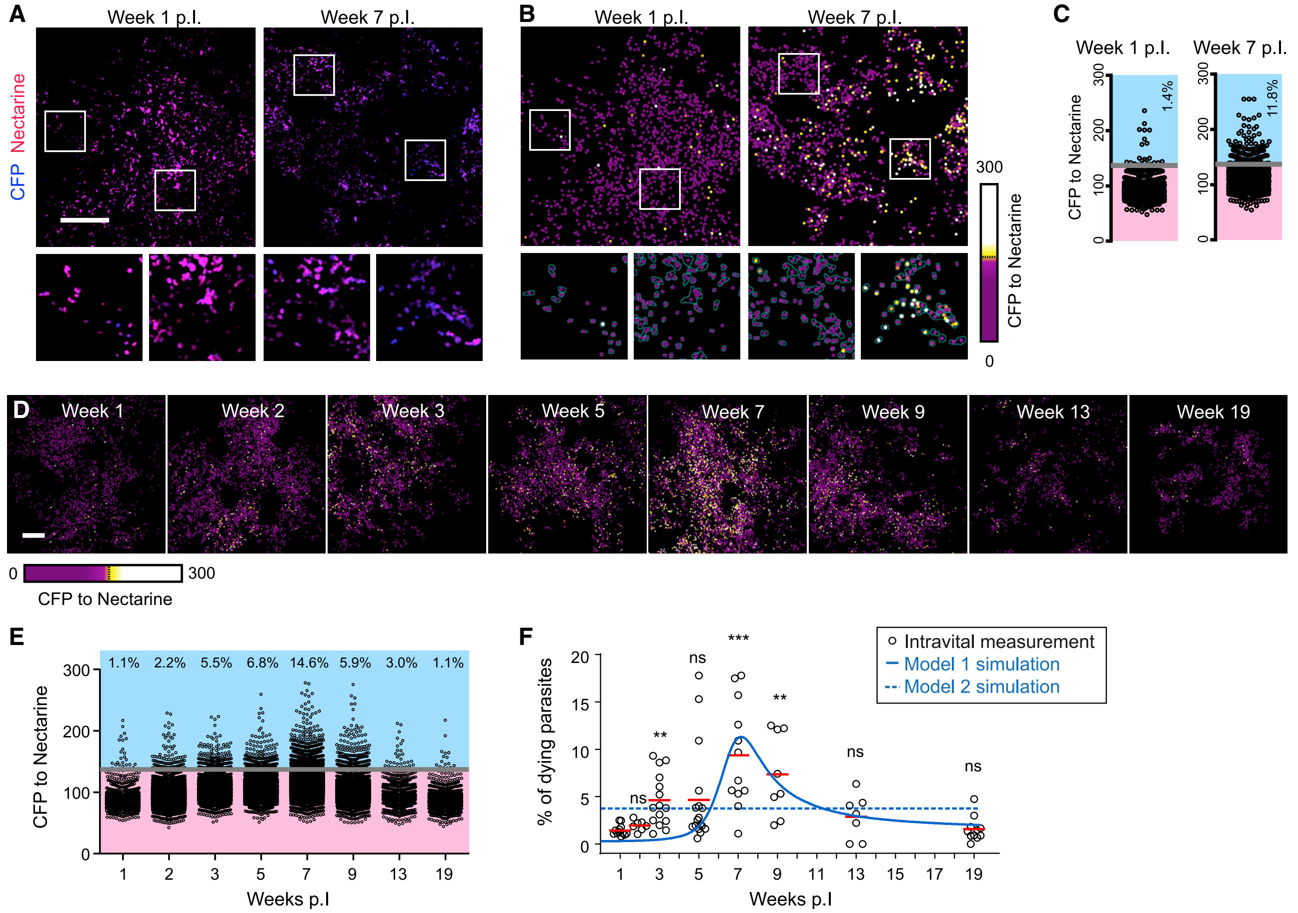

Figure 3. IV-2PM and modeling of *L. major* death over the course of the infection

Low-dose *L. major*^{necR} infection analyzed by IV-2PM at the indicated time points.

(A–C) Examples of infection sites with a low (1 wpi) and a high (7 wpi) frequency of dying. Segmented parasites in IV-2PM (A), death heatmaps (B), and quantifications (C) are shown.

(D) *L. major*^{necR} death heatmaps determined by IV-2PM throughout the infection. Projections of at least 14 z planes spaced 3 μm apart are shown in (A), (B), and (D) with scale bars of 100 μm.

(E) Quantification of images in (D).

(F) Analysis of 7 to 15 infected ears per time point, and comparison with simulated parasite death rates in model 1 (solid line) and model 2 (dashed line). Each symbol represents one infected ear. Horizontal bars denote the mean. ***$p < 0.001$; **$p < 0.01$; ns, not significant, according to Kruskal-Wallis with Dunn's post-test (comparison with 1 wpi). Data pooled from 2 to 3 independent experiments. See also Figure S3.

and persisted throughout the experiment (n = 13, Figure 2E). Parasites with a high CFP-to-mNectarine ratio could be detected for an average 3.5 h (Figure 2F), which provided a large-enough time span to assess the fraction of dying parasites also at single time points, without the necessity of tracking parasites individually (as exemplified in Figure 2B).

To test the reporter construct in the context of anti-*Leishmania* treatment, we measured parasite death in an established infection treated with liposomal amphotericin B (AmBisome). For this, WT mice were infected in the ear dermis with 5 × 10^3 *L. major*^{necR} metacyclics, and two weeks later they were either mock-treated or injected daily with AmBisome, which acts on *L. major* by causing parasite membrane permeabilization (Minodier et al., 2003; Solomon et al., 2011). We found that 6 days of treatment decreased pathogen burden, whereas no effect could be detected after only four daily injections of AmBisome (Figure 2G). We concluded that after five days of treatment, the antileishmanial action of AmBisome would be initiated, wherefore IV-2PM of the infected ear tissue was performed at this time point and revealed increased proportions of dying parasites in AmBisome-injected versus mock-treated animals (Figures 2H and 2I). Thus, the decrease in pathogen load induced by AmBisome is associated with the detection of increased death rates. Altogether, the new *L. major*^{necR} death reporter strain allowed faithful assessment of pathogen death rates *in vivo*.

To determine parasite death over the course of the immune response, we imaged the site of infection of *L. major*^{necR}-infected (5 × 10^3 metacyclics) mice by IV-2PM at time points up to 19 wpi. Acquired large-field microscopy images (Figure 3A) were segmented, converted into a heatmap of CFP-to-mNectarine ratios (Figure 3B), and quantified (Figure 3C). Importantly, although most parasites retained mNectarine fluorescence and appeared viable at 1 wpi, the proportion of *L. major* exhibiting a high CFP-to-mNectarine ratio gradually increased with the onset of

the immune response, being highest between 7 to 9 wpi (Figures 3A–3C), at which time point the immune effector response had reached its maximum (see Figure 1A). At later time points, parasite death rates decreased to low levels comparable to 1 wpi (Figures 3D–3F). Simulation of parasite death in mathematical models that included iNOS-dependent killing recapitulated the death rates measured over the infection time course (Figures 3F and S3G). In contrast, models in which only direct inhibition of proliferation contained the parasite (models 2 and 5) had to be excluded, because in these models, parasite death was constant over time (Figures 3F and S3G; Tables S3 and S4). Altogether, efficient pathogen killing occurs only around the peak of infection, whereas during the persistent phase of infection, pathogen numbers remain low in the absence of overt pathogen death.

L. major proliferation is high during immune cell recruitment but low during the persistence phase of the infection

Because pathogen killing peaks under full immune activation, decreased proliferation at later stages of the infection could explain how the *L. major* burden remains low during persistence with low killing. To explore such bimodal proliferation behavior in the context of the course of an immune response, we employed *L. major*^{SWITCH}. This well-established reporter strain allows proliferation measurement in the ongoing infection via dilution and turnover of the photoconvertible fluorescence protein mKikume (Heyde et al., 2018; Hurrell et al., 2017; Müller et al., 2013). Mice infected with *L. major*^{SWITCH} (5×10^3 metacyclics) were analyzed at different time points by photoconversion of mKikume; IV-2PM analysis of the dilution of red, photoconverted mKikume protein; and *de novo* production of green, non-photoconverted mKikume in proliferating parasites 48 h later (Figures 4A, 4B, and S4). Analysis over the whole course of the infection revealed that *L. major* proliferation was highest between 5 and 8 wpi (Figures 4C–4E), the period with the highest increase of recruited cells (see Figure 1B). Importantly, mathematical modeling of proliferation showed that models involving direct killing only (models 1 and 4) recapitulated the data from *in vivo* measurements with the *L. major*^{SWITCH} reporter strain better than models involving direct proliferation inhibition (models 2 and 5, Figures 4E and S4F). This suggested that *L. major* proliferation is not directly inhibited by iNOS activity, because iNOS expression and high *L. major* proliferation seem to be correlated (see Figure 1E).

Reduced presence of monocyte-derived cells dampens *L. major* proliferation in the absence of iNOS

To investigate whether the restriction of cellular recruitment per se can dampen pathogen proliferation, we sought to analyze the effect of lowered cell recruitment in the absence of iNOS activity. For this, we employed *Rag1*$^{-/-}$ mice, which are devoid of an adaptive immune response against *L. major*. Despite the lack of T cell-mediated iNOS induction, pathology and pathogen numbers have been shown to develop slowly in these mice, with a *L. major* burden comparable to that of WT animals at 5 wpi (Belkaid et al., 2000). Therefore, reduced monocyte-derived cell recruitment in *Rag1*$^{-/-}$ mice might reduce pathogen proliferation at early phases of infection and thus compensate for the lack of iNOS-mediated killing. As expected, *Rag1*$^{-/-}$ mice infected with 5×10^3 *L. major* metacyclics exhibited reduced monocyte recruitment and an absence of iNOS expression, but they displayed a parasite burden equal to that of WT controls (Figure 5A). Parasite killing determined by the *L. major*^{necR} death reporter strain was reduced in the *Rag1*$^{-/-}$ mice (Figures 5B–5D), and in line with previous observations (Müller et al., 2012), individual cells seemed to be more densely populated by *L. major* in *Rag1*$^{-/-}$ mice (Figure 5B, insets). In contrast, IV-2PM using the *L. major*^{SWITCH} proliferation reporter revealed that despite the absence of iNOS expression (Figure 5A), pathogen proliferation was diminished (Figures 5E–5G). This indicated that in *Rag1*$^{-/-}$ mice, reduced availability of recruited proliferation-permissive cells dampened pathogen proliferation in the absence of iNOS-mediated killing.

Next, we sought to corroborate the connection between iNOS inhibition and increased pathogen proliferation via uncontrolled recruitment of the permissive cellular compartment. For this, we blocked leukocyte extravasation using anti-CD18/CD49d antibodies while inhibiting iNOS using the inhibitor L-NIL (Figure 6A). As expected, iNOS inhibition alone resulted in an increase in lesion thickness (Postat et al., 2018), which could be reverted by blocking leukocyte extravasation through injection of anti-CD18/CD49d (Figure 6B). Likewise, by highlighting newly recruited cells (Figure S5A), we could show an increase in newly recruited monocyte-derived cells in iNOS-inhibited mKikume mice (Heyde et al., 2018; Nowotschin and Hadjantonakis, 2009), which was most pronounced in Ly6C^{+} phagocytes. Anti-CD18/CD49d injection blunted this recruitment even if iNOS was inhibited (Figures 6C and S5B–S5F). To measure pathogen proliferation in the combined absence of iNOS activity and blocked host cell recruitment, IV-2PM of the proliferation reporter strain *L. major*^{SWITCH} was employed. The increase in *L. major* proliferation observed upon iNOS inhibition was reverted upon anti-CD18/CD49d treatment (Figures 6D and 6E). This indicated that even under conditions at which pathogen killing is taking place, iNOS contributes to reducing *L. major* proliferation by restricting the recruitment of proliferation-permissive cells to the site of infection. In contrast, the effect of anti-CD18/CD49d on the absolute pathogen burden after 3 days of treatment was observable neither experimentally nor in simulations (Figures S6A and S6B). This suggested that inhibition of iNOS-mediated killing affected pathogen numbers faster than inhibition of cell recruitment did, which we also observed in the mathematical models (Figure S6C). In line with this finding, efficient turnover of recruited cells at the site of infection can require several days (Heyde et al., 2018).

Anti-CD18/CD49d administration, both with and without iNOS inhibition, increased the proportion of infected cells experimentally, as well as in simulations (Figures S6D–S6F), which could result from newly recruited cells that may dilute the infected cells before being infected themselves. To address long-term effects of a transient inhibition of iNOS and/or anti-CD18/CD49d administration, we simulated a 3-day inhibition of monocyte activation and/or blocked cell recruitment and modeled the subsequent 3 weeks of infection (Figure 6F). This approach predicted that transient inhibition of phagocyte activation would result in a lower pathogen burden and infection rate (Figures 6G, S6G, and S6H, blue curves), and a higher fraction of activated cells (Figure S6I). Model 4 predicted an even lower pathogen burden

CellPress
OPEN ACCESS

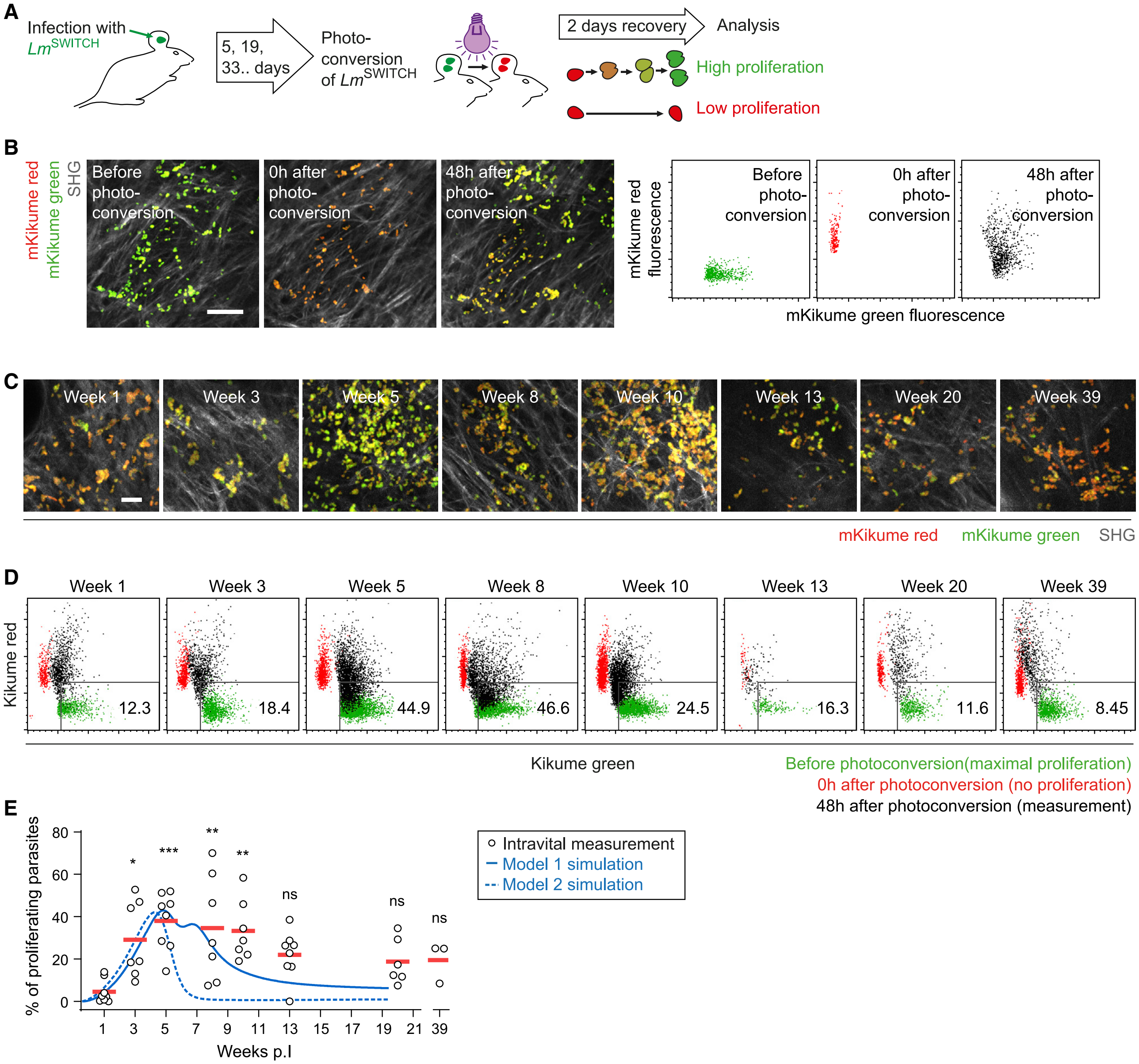

Figure 4. IV-2PM and modeling of *L. major* proliferation over the course of the infection

(A) Proliferation measurement setup. Low-dose *L. major*^{SWITCH}-infected ears photoconverted from green to red at indicated time points and imaged by IV-2PM 48 h later. Parasite proliferation results in recovery from red to green.

(B) Left: examples of segmented parasites before, 0 h after, and 48 h after photoconversion. The SH signal from collagen fibers is shown in gray. Scale bar, 50 μm. Right: green and red fluorescence content of the same segmented parasite objects before (green), 0 h after (red), and 48 h after (black) photoconversion.

(C) Examples of IV-2PM 48 h after photoconversion at different time points in the course of infection. Segmented red and green parasite fluorescence and collagen SH signals are shown. Scale bar, 20 μm. Maximum-intensity projections of at least 20 z planes spaced 3 μm apart are shown in (B) and (C).

(D) Quantification of pathogen proliferation. Data from measurements of the same site before (green), 0 h (red), and 48 h after (black) photoconversion are overlaid. Data normalized as described in STAR Methods.

(E) Pathogen proliferation rates analyzed over time and overlaid with modeled parasite proliferation rates in model 1 (solid line) and model 2 (dashed line). Each symbol represents one infected ear. Horizontal bars denote the mean. ***$p < 0.001$; **$p < 0.01$; *$p < 0.05$; ns, not significant, according to Kruskal-Wallis with Dunn's post-test (comparison with 1 wpi). Data pooled from 2 independent experiments. See also Figure S4.

and infection rate when phagocyte recruitment was transiently blocked (Figures S6G and S6H, pink and red curves). When we treated infected mice according to the modeled conditions (Figure 6F), we were able to confirm the predicted trends for the effects of iNOS, as well as recruitment blocking on the infection (Figures 6H and S6J). Thus, not only iNOS-mediated killing of the pathogen but also dampened phagocyte recruitment during acute infection affects the course of the infection later.

Because NO suppresses the respiratory capacity of monocytes (Postat et al., 2018), metabolic reprogramming of the

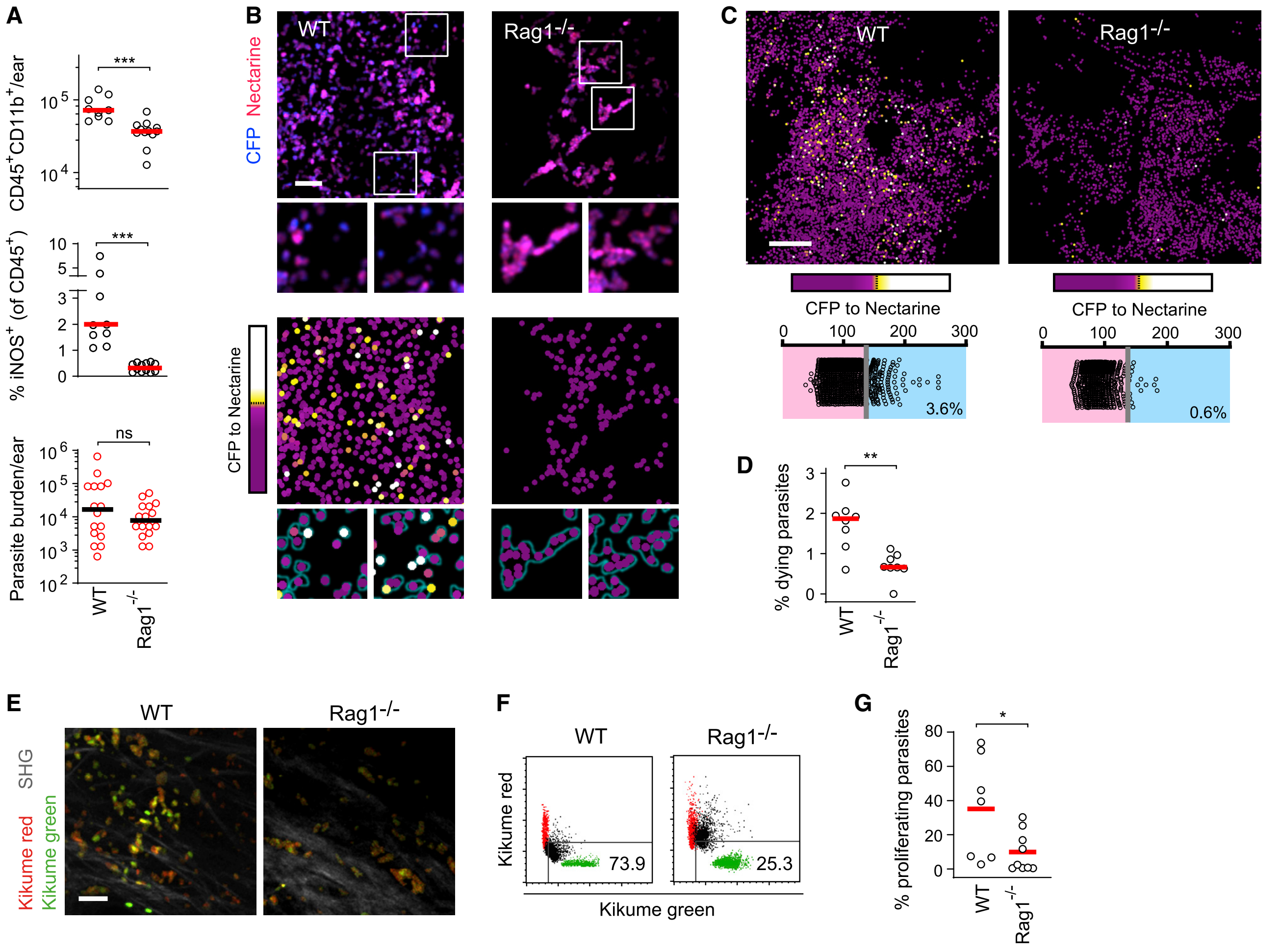

Figure 5. Lack of monocyte recruitment, no overt parasite killing, and low *L. major* proliferation in the absence of iNOS induction in *Rag1*$^{-/-}$ mice

(A–D) Low-dose *L. major*^{necR}-infected WT and *Rag1*$^{-/-}$ mice analyzed 5 wpi. (A) Recruited immune cells (top), iNOS expression (middle), and pathogen burden (bottom) at the site of infection. Each circle represents one infected ear. Data pooled from 2 (flow cytometry) to 3 (limiting dilution) experiments with at least 9 ears per group. (B–D) Assessment of parasite death in WT versus *Rag1*$^{-/-}$ mice employing the *L. major*^{necR} reporter strain. (B) IV-2PM (upper panel) and corresponding heatmaps of parasite death (lower panel). Projections of at least 20 z planes spaced 3 μm apart are shown. Scale bar, 20 μm. (C) Representative large-field IV-2PM in WT and *Rag1*$^{-/-}$ mice depicting the CFP-to-mNectarine ratios of single pathogens (upper panel) and corresponding quantitative analysis (lower panels). Projections of at least 25 z planes spaced 3 μm apart are shown. Scale bar, 100 μm. (D) Fraction of dying parasites in WT versus *Rag1*$^{-/-}$ in 8 infected ears per group. Data pooled from 2 independent experiments. Horizontal bars denote the mean. ***p < 0.001, according to Mann-Whitney test.

(E–G) Parasite proliferation in WT versus *Rag1*$^{-/-}$ mice employing IV-2PM of the reporter strain *L. major*^{SWITCH}. (E) IV-2PM examples of WT and *Rag1*$^{-/-}$ ears infected with *L. major*^{SWITCH} 48 h after photoconversion. Scale bar, 20 μm. Segmented and masked red and green channels, as well as the collagen SH signal (gray), are shown from one z plane of a three-dimensional image. (F) Quantifications of recovery from photoconversion (black) with measurements of the same site before (green) and 0 h after (red) photoconversion was overlaid. Data normalized as described in STAR Methods. (G) Fraction of proliferating parasites in WT versus *Rag1*$^{-/-}$ mice. Each symbol represents one imaged infection site. Data accumulated from at least 7 imaged areas acquired in 3 independent experiments. Horizontal bars represent the mean. **p < 0.001; *p < 0.05; ns, not significant, according to Mann-Whitney test in (A), (D), and (G).

See also Figure S5.

host cells might be responsible for the increased pathogen proliferation under iNOS inhibition. To address this, phagocytes isolated from infected control mice, L-NIL-treated mice (iNOS inhibition) and L-NIL+anti-CD18/CD49d-treated mice (iNOS inhibition and blocked recruitment) were subjected to metabolic flux analysis (Figure 6I). In addition, we analyzed cells from infected *Rag1*$^{-/-}$ mice, which exhibit low monocyte recruitment not due to iNOS activity (see Figure 5) but due to the lack of an adaptive immune response (Belkaid et al., 2000). As expected, iNOS inhibition augmented oxidative phosphorylation (Postat et al., 2018), whereas cells from infected control animals mainly engaged glycolysis (Figure 6I). Increased oxidative phosphorylation was also observed when phagocyte recruitment was blocked additionally to iNOS inhibition. Phagocytes from infected *Rag1*$^{-/-}$ mouse ears exhibited an even higher respiratory capacity (Figures 6J and 6K) and a primarily aerobic phenotype. This underlines that a lack of NO production in the absence of a T cell response results in high oxidative phosphorylation (Figure 6I, right panel). In line with these findings, inhibition of iNOS resulted in increased production of pro-IL-1β, which was also

CellPress
OPEN ACCESS

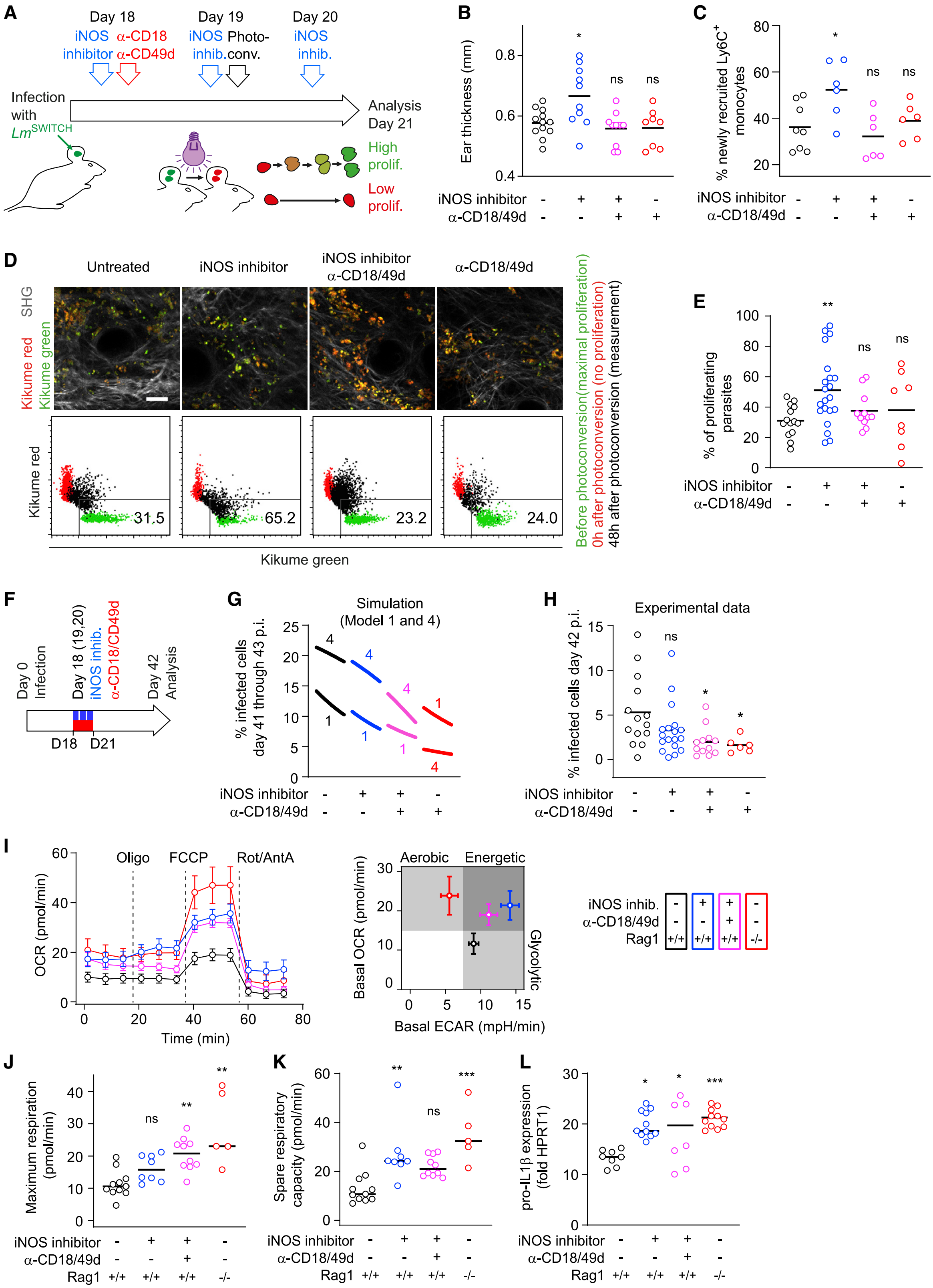

(legend on next page)

observed under conditions of impaired phagocyte recruitment (Figures 6L, S6K, and S6L). Therefore, *L. major* proliferation was mainly associated with the availability of recruited host phagocytes, not with the metabolic or inflammatory phenotype of these cells.

Monocyte administration, but not neutrophil recruitment, increases pathogen burden at later stages of the infection

To investigate the immediate effect of the presence of monocytes on pathogen burden, we injected non-activated or IFNγ/LPS-activated bone marrow monocytes into the site of infection during the persistence phase 10 wpi of high-dose infected mice (Figure S7A). Three days after injection, both the infection rate and the pathogen burden at the site of infection were increased for lesions injected with non-activated monocytes (Figures 7A and 7B), which could be detected in higher numbers than activated monocytes in the respective lesions (Figures 7C and S7B). The total number of $CD11b^{+}Ly6G^{-}$ phagocytes and neutrophils ($CD11b^{+}Ly6G^{+}$) remained unchanged; thus, the increase in pathogen numbers and infection rate correlated only with the fraction of new monocytes at the site of infection (Figures 7D and S7C). To test the impact of a neutrophil-dominated inflammation on pathogen numbers, we used 2,4-dinitrofluorobenzene (DNFB) to induce contact dermatitis (Bonneville et al., 2007) at 10 wpi. Neutrophil numbers were increased 3 days after DNFB application compared with vehicle control. In contrast, $CD11b^{+}Ly6G^{-}$ phagocyte numbers remained unchanged (Figures 7E, 7F, and S7D). Neither the overall pathogen burden nor the infection rate in cells isolated from the infection site were affected by DNFB (Figures 7G and 7H). Thus, monocyte administration, but not neutrophil recruitment, can increase pathogen numbers in the late phase of the infection.

Altogether, these findings suggest that iNOS promotes containment of *L. major* through direct killing in the acute phase and by restricting recruitment of permissive monocyte-derived cells, thus dampening pathogen proliferation during the chronic phase of infection.

DISCUSSION

In the present study, we observed that iNOS activity exerted intracellular pathogen containment via two distinct ways: direct killing and restriction of the permissive host cell. Using *L. major* as a tool to evaluate the mode of action of the immune response, we found that intracellular pathogens were efficiently killed mainly at the peak of infection. In contrast, a low pathogen proliferation rate was attributable to control of the infection burden later. Without inflammatory cell recruitment, intracellular pathogen proliferation was reduced even in the absence of functional iNOS. Antibody-mediated blockade of monocyte recruitment abolished the effect of iNOS inhibition on pathogen proliferation, whereas *in situ* monocyte deposition, but not neutrophil recruitment, increased pathogen burden at late stages of the infection.

Low-level persistent infections increase a pathogen's chances of transmission, and many pathogens have evolved strategies to prolong their survival within host phagocytes without overt pathology (Mandell and Beverley, 2017; Monack et al., 2004; Pawlowski et al., 2012). In this context, T helper (Th) 1 responses required for efficient NO production and pathogen containment induce not only intracellular defense mechanisms within phagocytes but also chemokines responsible for the recruitment of monocytes, which can be hijacked as permissive host cells (Heyde et al., 2018; Romano et al., 2017; Shi and Pamer, 2011; Terrazas et al., 2017). In addition, the balance of Th1 and Th2 immunity dictates the outcome of an early infection via regulation of the size of the permissive host cell reservoir (Carneiro et al., 2020). By inhibiting inflammatory cell recruitment, NO exerts functions beyond its well-known antimicrobial activity (Postat et al., 2018). Our data now show that the NO-mediated feedback, which restricts the cellular compartment permissive for *L. major*, represents an antimicrobial mode of action per se.

Figure 6. Blocking monocyte recruitment abolishes the iNOS inhibition-dependent increase in *L. major* proliferation and affects the course of infection

(A) Experimental setup, high-dose infections.

(B) Ear thickness at day 21.

(C) Fraction of $Ly6C^{+}$ cells (gated according to Figure S6) recruited within the last four days before analysis. Each dot in (A) and (B) represents an individual ear. Data pooled from two independent experiments.

(D) Parasite proliferation determined by IV-2PM. Top row: IV-2PM data. Scale bar, 20 μm. Segmented red and green channels and collagen SH signals (gray) are shown from one z plane of a three-dimensional image. Bottom row: quantifications (black) of the images shown with measurements of the same site before (green) and 0 h after (red) photoconversion was overlaid. Data normalized as described in STAR Methods.

(E) Fraction of proliferating parasites. Each symbol represents one imaged area. Data from at least 8 imaged areas acquired in three independent experiments.

(F) Setup for transient iNOS inhibition (blue) and anti-CD18/CD49d-mediated recruitment blocking (red).

(G) Simulated infection rates for models 1 and 4 (fraction of $M_i + M_{ai}$ among all monocytes) modeled according to (F). Curves show the time course from day 41 to day 43. Numbers indicate the model (1 or 4) for each curve.

(H) Fraction of infected $Ly6G^{-}CD11b^{+}$ monocyte-derived cells determined by flow cytometry at day 42 p.i., treated according to (F). Each circle represents one infected ear.

(I–K) Metabolic flux analysis of monocyte-derived cells isolated from the infected ear tissue at 3 wpi. (I) Left panel: oxygen consumption rate (OCR) in cells from control (black), L-NIL-treated (blue), L-NIL and anti-CD18/CD49d-treated (magenta), and *Rag1*$^{-/-}$ (red) animals. Right panel: basal OCR and extracellular acidification rate (ECAR). Circles show the mean ± SEM of at least 5 replicates, consisting of pools of isolated monocytes normalized to 10^5 viable cells, collected in three independent experiments from 16 infected ears per group. (J and K) Maximum respiration (J) and spare respiratory capacity (K) under the different conditions described under (I). Each circle shows one replicate of 10^5 pooled viable cells, collected in three independent experiments from 16 infected ears per group in total.

(L) Quantitative PCR of pro-IL-1β expression by monocytes as described under (I). Each circle represents one infected ear.

Horizontal bars denote the median. ns, not significant; *p < 0.05; **p < 0.01; ***p < 0.001, according to Kruskal-Wallis with Dunn's post-test (comparison with control [no treatment, *Rag1*$^{+/+}$ where applicable] condition) in (B), (C), (E), (H), and (J)–(L). See also Figure S6.

CellPress
OPEN ACCESS

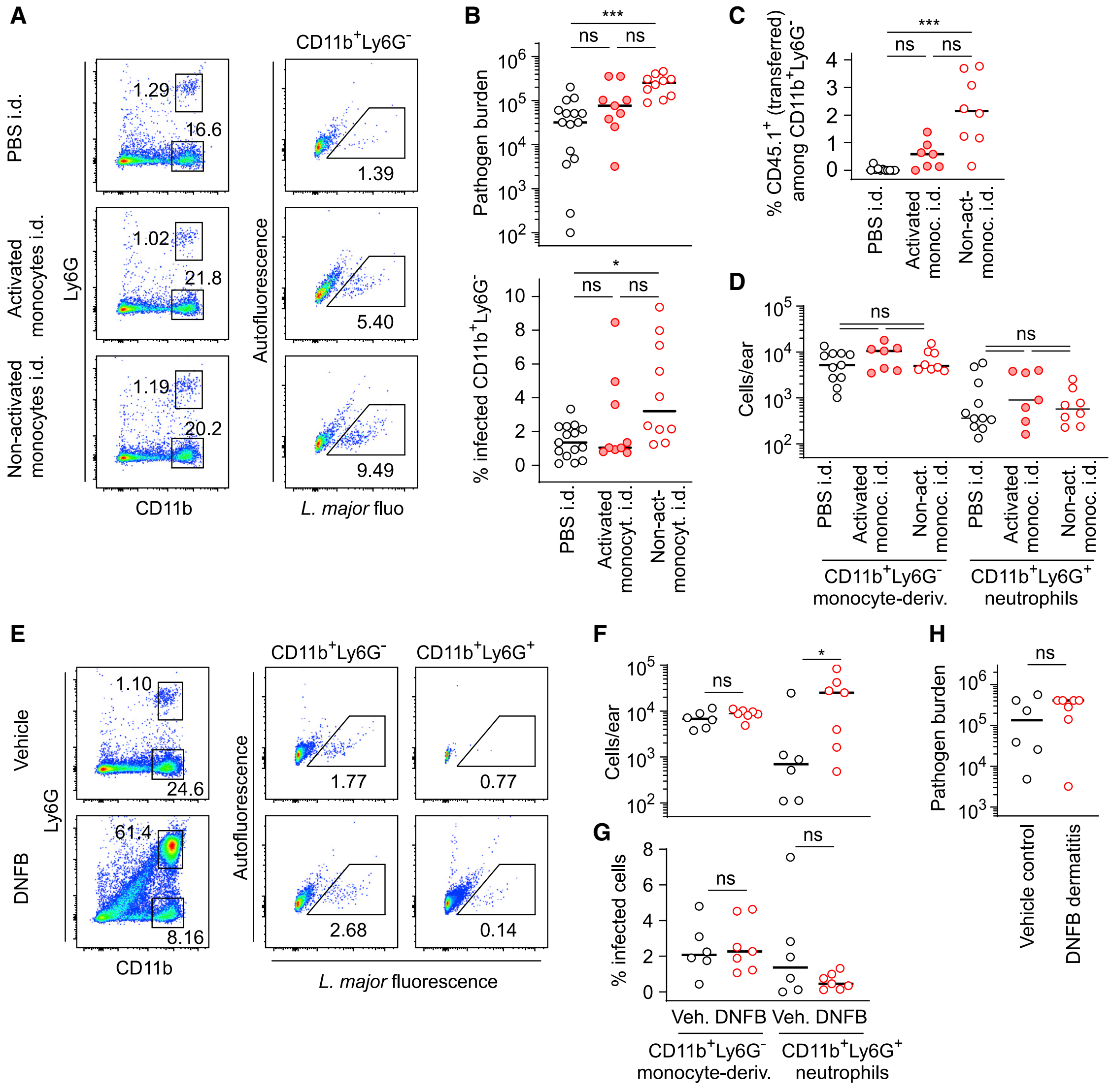

Figure 7. Monocyte administration, but not neutrophil recruitment, increases pathogen burden at later stages of the infection

(A) Flow cytometry analysis of high-dose infection sites injected at 10 wpi with PBS (top row) or with 2 × 10^6 iNOS-expressing (middle row) or non-activated (bottom row) bone marrow monocytes, analyzed 3 days after injection.

(B) Analysis of pathogen burden (top panel) and infection rate in Ly6G$^-$CD11b$^+$ monocyte-derived cells by flow cytometry (bottom panel). Each circle in (B)–(D) represents one infected ear at 10 wpi (high-dose infection) injected with PBS (black circles) or with activated (filled red circles) or non-activated (empty red circles) bone marrow monocytes into the infection site three days before analysis.

(C) Fraction of injected CD45.1$^+$ donor cells among all Ly6G$^-$CD11b$^+$ monocyte-derived cells at the site of infection.

(D) Total number of Ly6G$^-$CD11b$^+$ monocyte-derived cells (left) and Ly6G$^+$CD11b$^+$ neutrophils (right) isolated from the site of infection. Horizontal bars in (B)–(D) denote the median.

(E) Flow cytometry of infection sites treated epicutaneously with vehicle (top panels) DNFB at 10 wpi (high-dose infection), 3 days before analysis.

(F– H) Cell number (F) and fraction of infected cells (G) determined for Ly6G$^-$CD11b$^+$ monocyte-derived cells (left) and Ly6G$^+$CD11b$^+$ neutrophils (right) isolated from the site of infection, as well as pathogen burden (H). Black circles in (F)–(H) represent vehicle-treated infection sites, and red circles represent DNFB-treated infection sites. Each circle represents one infected ear. Data collected in at least two independent experiments. Horizontal bars denote the median.

ns, not significant; *$p < 0.05$; ***$p < 0.001$, according to Kruskal-Wallis with Dunn's post-test in (B)–(D), (F), and (G) or Mann-Whitney test in (H). See also Figure S7.

iNOS inhibition results in expansion of pathogen numbers even after overt pathology has subsided (Stenger et al., 1996). However, we observed that substantial pathogen killing coincided with heightened pathology and immune activation. In line with the notion that pathogen control must also rely on mechanisms different from direct killing, *Leishmania* persists not in immunoprivileged cell types but instead within phagocytes that express iNOS (Mandell and Beverley, 2017). Together with our findings on a peak of pathogen death under full immune activation, this suggests that although iNOS contributes to controlling the pathogen at all times of the infection, the amount of NO produced during the persistence phase might not be high enough to exert direct pathogen killing. In our mathematical models and reporter system analyses, the peak in pathogen proliferation coincided

with high immune cell recruitment but preceded the peak of pathogen killing by 1–2 weeks. This highlights two interesting time points: At the peak of pathogen proliferation, despite high iNOS expression, the phagocyte population is constantly replenished by newly recruited cells. Then, 1–2 weeks later, the number of recruited phagocytes and the pathogen burden have reached their maximum. Because efficient iNOS induction allowing pathogen killing might take several days (Olekhnovitch et al., 2014), it is conceivable that the massive recruitment of phagocytes temporarily dilutes the effective fraction of NO-producing cells. Furthermore, although iNOS-independent effects of Th1-related cytokines, especially of tumor necrosis factor (TNF), are thought to contribute to *Leishmania* containment (Fromm et al., 2012; Rocha et al., 2007; Wilhelm et al., 2001), in our model system, iNOS expression in recruited monocytes is tightly linked with both interferon-γ receptor (IFNγR) signaling and tumor necrosis factor receptor (TNFR) signaling (Olekhnovitch et al., 2014).

Because iNOS dampens *L. major* proliferation both *in vitro* and *in vivo* (Kloehn et al., 2015; Müller et al., 2013; Olekhnovitch et al., 2014), it would be tempting to speculate that NO exerts inhibition of pathogen proliferation directly; thus, overall pathogen proliferation would be inversely correlated with immune activation. However, *Leishmania* proliferation has been postulated to be highest at the peak of the infection (Belkaid et al., 2000; Jara et al., 2019; Mandell and Beverley, 2017). In addition, newly recruited inflammatory phagocytes represent a niche for rapidly proliferating *L. major* (Heyde et al., 2018). In line with this, our modeling and experimental data showed that direct proliferation inhibition by NO is neither required nor sufficient to explain the observed course of events and that pathogen proliferation is highest at the peak of the immune response. Thus, the increased proliferation rate upon iNOS inhibition is mainly attributable not to non-controlled growth but to increased availability of permissive inflammatory monocytes, because it could be reverted by blocking phagocyte recruitment. NO restricts the pathogen on a cell-extrinsic, tissue-wide level (Olekhnovitch et al., 2014), and our findings connect this observation with the observations of iNOS-dependent dampening of both pathogen proliferation (Müller et al., 2013) and monocyte-derived cell recruitment (Postat et al., 2018). These are likely the result of the same mode of action: NO affects phagocyte recruitment, leading to a lack of proliferation-permissive host cells in the tissue, which in turn restricts pathogen proliferation non-lethally.

We observed that *in situ* deposition of monocytes reactivated the infection and increased pathogen numbers at late stages of the infection. This is especially interesting with regard to clinical data showing that skin inflammation can induce *Leishmania* lesions in persistently infected patients (Kerr et al., 2009; Rab et al., 1992). The phenotype of such an inflammation might be decisive for whether the lesions are reactivated, because the parasite required monocyte-derived cells rather than neutrophils to efficiently proliferate. Non-activated monocytes increased pathogen numbers more than IFNγ/LPS-activated monocytes did. Although it would be tempting to speculate that iNOS expression in the activated monocytes inhibits the pathogen, the fraction of activated cells remained below 5%. According to previous data, more than 10% of cells need to produce iNOS to control the pathogen (Olekhnovitch et al., 2014), so the effect of monocyte activation most likely arises from altered efficiency in transferring viable cells to the infection site.

NO limits immunopathology in infections with various pathogens, including *Pseudomonas* (Hopkins et al., 2006), *Brucella* (Lacey et al., 2019), and *Mycobacterium* spp. For the latter, excessive phagocyte recruitment has been modeled to be detrimental for containment (Segovia-Juarez et al., 2004). Moreover, iNOS deficiency results in an increased neutrophil recruitment and a lack of *Mycobacterium* clearance (Beisiegel et al., 2009). In addition, NO inhibits IL-1β-dependent neutrophil influx, preventing the establishment of an inflammatory environment permissive for *Mycobacterium* (Mishra et al., 2013, 2017). Although the contributions of killing versus proliferation inhibition in these models were not fully established, NO-mediated recruitment inhibition seems to be a general mechanism by which the immune system counteracts the establishment of conditions suitable for pathogen proliferation.

Because reduced cell recruitment results from NO-dependent dampening of mitochondrial respiration within monocyte-derived cells (Postat et al., 2018), the reprogrammed metabolism of potential host cells could, in principle, by itself alter the pathogen proliferation rate observed upon iNOS inhibition. However, we found that changes in pathogen proliferation mainly depended on the availability of newly recruited monocyte-derived cells, not on the metabolic state of the phagocytes. In addition, transient inhibition of iNOS early in the infection (i.e., 3 wpi, high-dose infection) resulted in lower infection several weeks later, both in our modeling and in our experimental data. This is most likely due to an accelerated course of the infection shown for high infection doses, which results in faster control (Belkaid et al., 2000). Antibody-mediated blocking of immune cell recruitment additionally dampened pathogen burden over the long-term perspective, but in contrast to iNOS inhibition, it did not do so immediately after three days of treatment. This different timing can be explained by the different kinetics expected for the inhibition of pathogen killing versus the effect of a shortage of newly recruited host cells: Upon iNOS inhibition, NO concentration in the tissue is expected to decrease rapidly because of a half-life of minutes (Thomas et al., 2001). Thus, iNOS-dependent pathogen killing should rapidly subside upon iNOS inhibition. In contrast, the turnover time of recently recruited cells is in the range of several days (Heyde et al., 2018). Thus, even if phagocyte recruitment is blocked instantaneously, recently recruited phagocytes left at the site of infection might allow an increase in parasite burden. Nevertheless, the observed decrease in *L. major* proliferation has important effects on the course of the infection later.

Altogether, our data suggest a dual role for NO in the containment of *L. major*, not only permitting the direct killing of the pathogen mainly at the peak of the immune response but also restricting the supply of infectable phagocytes. This sublethal mode of containment may represent a critical constituent of the equilibration between the immune response and the pathogen during persistent infections.

Limitations of the study

We used infection and mathematical models that focused on the interaction between myeloid cells and intracellular pathogen, whereas the contribution of the adaptive immune response

CellPress
OPEN ACCESS

was included only via the analysis of iNOS as a function of T helper activation. Modeling combined with *in vivo* analyses that include the interplay of pathogen density and iNOS with the activation and balance of distinct T cell effector functions will be instrumental to dissect the induction, execution, and contraction of immune responses against intracellular pathogens.

STAR★METHODS

Detailed methods are provided in the online version of this paper and include the following:

- KEY RESOURCES TABLE
- RESOURCE AVAILABILITY
  - Lead contact
  - Materials availability
  - Data and code availability
- EXPERIMENTAL MODEL AND SUBJECT DETAILS
  - Mice
  - Pathogen
  - Bone marrow-derived monocytes
  - Bone marrow-derived neutrophils
  - Peritoneal macrophages
- METHOD DETAILS
  - *In vivo* infections
  - *In vitro* infections
  - Parasite pH manipulation *in vitro*
  - iNOS inhibition and blocking of cell recruitment
  - AmBisome treatment
  - Monocyte deposition
  - Irritant contact dermatitis
  - Determination of ear swelling
  - Extraction of leukocytes from infected ears
  - Limiting dilution assay
  - Flow cytometry
  - Photoconversion
  - Intravital 2-photon microscopy (IV-2PM)
  - Metabolic analyses
  - Quantitative PCR analysis
  - Mathematical models and model selection
  - Cell-intrinsic properties
  - Cell-intrinsic and extrinsic properties
  - Bootstrapping analysis of the fitted model parameters
- QUANTIFICATION AND STATISTICAL ANALYSIS
  - IV-2PM image processing and quantification of parasite death
  - Image processing and quantification for proliferation measurement
  - Statistical analysis

SUPPLEMENTAL INFORMATION

Supplemental information can be found online at https://doi.org/10.1016/j.immuni.2021.09.021.

ACKNOWLEDGMENTS

Invaluable discussions with Tatjana Ruhl, Christa Zellweger, Toni Aebischer, David Kilias, Thomas Tüting, and all members of the Müller lab are gratefully acknowledged. We also thank Ute Bank and Thomas Schüler for reagents and Jan Dudeck, Roland Hartig, Guido Höbbel, the MPBIC platform, and ZTL of the Medical Faculty OvGU for excellent technical support. This work was supported by funding from the European Research Council (ERC) under the European Union's Horizon 2020 research and innovation program (StG ImmProDynamics, grant agreement 714233 to A.J.M.), the German Research Foundation (DFG) (SFB854-Z01, SFB854-B31, MU 3744/2-1, MU3744/4-1, and SPP2225 [MU3744/5-1] to A.J.M. and KA 4514/2-1 to S. Kahlfuß), and the federal state of Saxony-Anhalt and the European Regional Development Fund (project NeutrEat to A.J.M.).

AUTHOR CONTRIBUTIONS

P.F., M.A., and A.J.M. conceived and designed the research; P.F., M.A., A. Siokis, J. Handschuh, P.G., A.K., I.S., Y.F., J.S., S.H., J.M., and A. Schröder performed the experiments; P.F., J. Handschuh, M.A., A.K., S. Kahlfuß, J.M., L.P., and A.J.M. evaluated the experimental data; P.F., M.A., A. Siokis, P.A.R., J. Huehn, S.B., M.M.-H., and A.J.M. discussed and A. Siokis, P.A.R., S.B., and M.M.-H. established the mathematical model; J.B., S. Khailaie, G.Z., A.D., S. Kahlfuß, G.F.S., P.B., and B.S. provided critical technology; P.F., J. Handschuh, A. Siokis, and A.J.M. wrote the manuscript; and all authors reviewed and discussed the manuscript.

DECLARATION OF INTERESTS

The authors declare no competing interests.

Received: December 3, 2020
Revised: August 4, 2021
Accepted: September 28, 2021
Published: October 22, 2021

REFERENCES

Abidin, B.M., Hammami, A., Stäger, S., and Heinonen, K.M. (2017). Infection-adapted emergency hematopoiesis promotes visceral leishmaniasis. PLoS Pathog. *13*, e1006422.

Antoine, J.C., Prina, E., Lang, T., and Courret, N. (1998). The biogenesis and properties of the parasitophorous vacuoles that harbour *Leishmania* in murine macrophages. Trends Microbiol. *6*, 392–401.

Beisiegel, M., Kursar, M., Koch, M., Loddenkemper, C., Kuhlmann, S., Zedler, U., Stäber, M., Hurwitz, R., and Kaufmann, S.H. (2009). Combination of host susceptibility and virulence of Mycobacterium tuberculosis determines dual role of nitric oxide in the protection and control of inflammation. J. Infect. Dis. *199*, 1222–1232.

Belkaid, Y., Hoffmann, K.F., Mendez, S., Kamhawi, S., Udey, M.C., Wynn, T.A., and Sacks, D.L. (2001). The role of interleukin (IL)-10 in the persistence of *Leishmania major* in the skin after healing and the therapeutic potential of anti-IL-10 receptor antibody for sterile cure. J. Exp. Med. *194*, 1497–1506.

Belkaid, Y., Mendez, S., Lira, R., Kadambi, N., Milon, G., and Sacks, D. (2000). A natural model of *Leishmania major* infection reveals a prolonged "silent" phase of parasite amplification in the skin before the onset of lesion formation and immunity. J. Immunol. *165*, 969–977.

Belkaid, Y., Piccirillo, C.A., Mendez, S., Shevach, E.M., and Sacks, D.L. (2002). CD4+CD25+ regulatory T cells control *Leishmania major* persistence and immunity. Nature *420*, 502–507.

Binder, S.C., Hernandez-Vargas, E.A., and Meyer-Hermann, M. (2015). Reducing complexity: An iterative strategy for parameter determination in biological networks. Comput. Phys. Commun. *190*, 15–22.

Bogdan, C. (2015). Nitric oxide synthase in innate and adaptive immunity: an update. Trends Immunol. *36*, 161–178.

Bonneville, M., Chavagnac, C., Vocanson, M., Rozieres, A., Benetiere, J., Pernet, I., Denis, A., Nicolas, J.F., and Hennino, A. (2007). Skin contact irritation conditions the development and severity of allergic contact dermatitis. J. Invest. Dermatol. *127*, 1430–1435.

Breart, B., Lemaître, F., Celli, S., and Bousso, P. (2008). Two-photon imaging of intratumoral CD8+ T cell cytotoxic activity during adoptive T cell therapy in mice. J. Clin. Invest. *118*, 1390–1397.

Carneiro, M.B., Lopes, M.E., Hohman, L.S., Romano, A., David, B.A., Kratofil, R., Kubes, P., Workentine, M.L., Campos, A.C., Vieira, L.Q., and Peters, N.C. (2020). Th1-Th2 Cross-Regulation Controls Early *Leishmania* Infection in the Skin by Modulating the Size of the Permissive Monocytic Host Cell Reservoir. Cell Host Microbe *27*, 752–768.e7.

Chandrasekar, B., Deobagkar-Lele, M., Victor, E.S., and Nandi, D. (2013). Regulation of chemokines, CCL3 and CCL4, by interferon γ and nitric oxide synthase 2 in mouse macrophages and during *Salmonella enterica* serovar Typhimurium infection. J. Infect. Dis. *207*, 1556–1568.

da Silva, R., and Sacks, D.L. (1987). Metacyclogenesis is a major determinant of *Leishmania* promastigote virulence and attenuation. Infect. Immun. *55*, 2802–2806.

De Trez, C., Magez, S., Akira, S., Ryffel, B., Carlier, Y., and Muraille, E. (2009). iNOS-producing inflammatory dendritic cells constitute the major infected cell type during the chronic *Leishmania major* infection phase of C57BL/6 resistant mice. PLoS Pathog. *5*, e1000494.

Fromm, P.D., Kling, J., Mack, M., Sedgwick, J.D., and Körner, H. (2012). Loss of TNF signaling facilitates the development of a novel Ly-6C(low) macrophage population permissive for *Leishmania major* infection. J. Immunol. *188*, 6258–6266.

Grainger, J.R., Wohlfert, E.A., Fuss, I.J., Bouladoux, N., Askenase, M.H., Legrand, F., Koo, L.Y., Brenchley, J.M., Fraser, I.D., and Belkaid, Y. (2013). Inflammatory monocytes regulate pathologic responses to commensals during acute gastrointestinal infection. Nat. Med. *19*, 713–721.

Handman, E., Hocking, R.E., Mitchell, G.F., and Spithill, T.W. (1983). Isolation and characterization of infective and non-infective clones of *Leishmania tropica*. Mol. Biochem. Parasitol. *7*, 111–126.

Hasenberg, M., Köhler, A., Bonifatius, S., Borucki, K., Riek-Burchardt, M., Achilles, J., Männ, L., Baumgart, K., Schraven, B., and Gunzer, M. (2011). Rapid immunomagnetic negative enrichment of neutrophil granulocytes from murine bone marrow for functional studies *in vitro* and *in vivo*. PLoS ONE *6*, e17314.

Heyde, S., Philipsen, L., Formaglio, P., Fu, Y., Baars, I., Höbbel, G., Kleinholz, C.L., Seiß, E.A., Stettin, J., Gintschel, P., et al. (2018). CD11c-expressing Ly6C+CCR2+ monocytes constitute a reservoir for efficient *Leishmania* proliferation and cell-to-cell transmission. PLoS Pathog. *14*, e1007374.

Hopkins, N., Gunning, Y., O'Croinin, D.F., Laffey, J.G., and McLoughlin, P. (2006). Anti-inflammatory effect of augmented nitric oxide production in chronic lung infection. J. Pathol. *209*, 198–205.

Hurrell, B.P., Beaumann, M., Heyde, S., Regli, I.B., Müller, A.J., and Tacchini-Cottier, F. (2017). Frontline Science: *Leishmania mexicana* amastigotes can replicate within neutrophils. J. Leukoc. Biol. *102*, 1187–1198.

Janssen, J.J.E., Lagerwaard, B., Bunschoten, A., Savelkoul, H.F.J., van Neerven, R.J.J., Keijer, J., and de Boer, V.C.J. (2021). Novel standardized method for extracellular flux analysis of oxidative and glycolytic metabolism in peripheral blood mononuclear cells. Sci. Rep. *11*, 1662.

Jara, M., Maes, I., Imamura, H., Domagalska, M.A., Dujardin, J.C., and Arevalo, J. (2019). Tracking of quiescence in *Leishmania* by quantifying the expression of GFP in the ribosomal DNA locus. Sci. Rep. *9*, 18951.

Johnson, D.E., Ai, H.W., Wong, P., Young, J.D., Campbell, R.E., and Casey, J.R. (2009). Red fluorescent protein pH biosensor to detect concentrative nucleoside transport. J. Biol. Chem. *284*, 20499–20511.

Kerr, A., Dawe, R.S., Nathwani, D., Evans, A.T., and Ferguson, J. (2009). Presentation of leishmaniasis (*Leishmania infantum*) in the skin of a patient with severe atopic dermatitis. Br. J. Dermatol. *161*, 202–203.

Kloehn, J., Saunders, E.C., O'Callaghan, S., Dagley, M.J., and McConville, M.J. (2015). Characterization of metabolically quiescent *Leishmania* parasites in murine lesions using heavy water labeling. PLoS Pathog. *11*, e1004683.

Kobayashi, Y. (2010). The regulatory role of nitric oxide in proinflammatory cytokine expression during the induction and resolution of inflammation. J. Leukoc. Biol. *88*, 1157–1162.

Lacey, C.A., Chambers, C.A., Mitchell, W.J., and Skyberg, J.A. (2019). IFN-γ-dependent nitric oxide suppresses Brucella-induced arthritis by inhibition of inflammasome activation. J. Leukoc. Biol. *106*, 27–34.

Lemesre, J.L., Sereno, D., Daulouède, S., Veyret, B., Brajon, N., and Vincendeau, P. (1997). *Leishmania* spp.: nitric oxide-mediated metabolic inhibition of promastigote and axenically grown amastigote forms. Exp. Parasitol. *86*, 58–68.

León, B., López-Bravo, M., and Ardavín, C. (2007). Monocyte-derived dendritic cells formed at the infection site control the induction of protective T helper 1 responses against *Leishmania*. Immunity *26*, 519–531.

Liew, F.Y., Millott, S., Parkinson, C., Palmer, R.M., and Moncada, S. (1990). Macrophage killing of *Leishmania* parasite *in vivo* is mediated by nitric oxide from L-arginine. J. Immunol. *144*, 4794–4797.

MacMicking, J.D., North, R.J., LaCourse, R., Mudgett, J.S., Shah, S.K., and Nathan, C.F. (1997). Identification of nitric oxide synthase as a protective locus against tuberculosis. Proc. Natl. Acad. Sci. USA *94*, 5243–5248.

Mandell, M.A., and Beverley, S.M. (2017). Continual renewal and replication of persistent *Leishmania major* parasites in concomitantly immune hosts. Proc. Natl. Acad. Sci. USA *114*, E801–E810.

Minodier, P., Retornaz, K., Horelt, A., and Garnier, J.M. (2003). Liposomal amphotericin B in the treatment of visceral leishmaniasis in immunocompetent patients. Fundam. Clin. Pharmacol. *17*, 183–188.

Mishra, B.B., Lovewell, R.R., Olive, A.J., Zhang, G., Wang, W., Eugenin, E., Smith, C.M., Phuah, J.Y., Long, J.E., Dubuke, M.L., et al. (2017). Nitric oxide prevents a pathogen-permissive granulocytic inflammation during tuberculosis. Nat. Microbiol. *2*, 17072.

Mishra, B.B., Rathinam, V.A., Martens, G.W., Martinot, A.J., Kornfeld, H., Fitzgerald, K.A., and Sassetti, C.M. (2013). Nitric oxide controls the immunopathology of tuberculosis by inhibiting NLRP3 inflammasome-dependent processing of IL-1β. Nat. Immunol. *14*, 52–60.

Misslitz, A., Mottram, J.C., Overath, P., and Aebischer, T. (2000). Targeted integration into a rRNA locus results in uniform and high level expression of transgenes in *Leishmania* amastigotes. Mol. Biochem. Parasitol. *107*, 251–261.

Monack, D.M., Mueller, A., and Falkow, S. (2004). Persistent bacterial infections: the interface of the pathogen and the host immune system. Nat. Rev. Microbiol. *2*, 747–765.

Moreau, H.D., Lemaître, F., Terriac, E., Azar, G., Piel, M., Lennon-Dumenil, A.M., and Bousso, P. (2012). Dynamic in situ cytometry uncovers T cell receptor signaling during immunological synapses and kinapses *in vivo*. Immunity *37*, 351–363.

Müller, A.J., Aeschlimann, S., Olekhnovitch, R., Dacher, M., Späth, G.F., and Bousso, P. (2013). Photoconvertible pathogen labeling reveals nitric oxide control of *Leishmania major* infection *in vivo* via dampening of parasite metabolism. Cell Host Microbe *14*, 460–467.

Müller, A.J., Filipe-Santos, O., Eberl, G., Aebischer, T., Späth, G.F., and Bousso, P. (2012). CD4+ T cells rely on a cytokine gradient to control intracellular pathogens beyond sites of antigen presentation. Immunity *37*, 147–157.

Norman, M.U., Zbytnuik, L., and Kubes, P. (2008). Interferon-gamma limits Th1 lymphocyte adhesion to inflamed endothelium: a nitric oxide regulatory feedback mechanism. Eur. J. Immunol. *38*, 1368–1380.

Nowotschin, S., and Hadjantonakis, A.K. (2009). Use of KikGR a photoconvertible green-to-red fluorescent protein for cell labeling and lineage analysis in ES cells and mouse embryos. BMC Dev. Biol. *9*, 49.

Okwor, I., and Uzonna, J. (2008). Persistent parasites and immunologic memory in cutaneous leishmaniasis: implications for vaccine designs and vaccination strategies. Immunol. Res. *41*, 123–136.

Olekhnovitch, R., Ryffel, B., Müller, A.J., and Bousso, P. (2014). Collective nitric oxide production provides tissue-wide immunity during *Leishmania* infection. J. Clin. Invest. *124*, 1711–1722.

Ortega-Gómez, A., Perretti, M., and Soehnlein, O. (2013). Resolution of inflammation: an integrated view. EMBO Mol. Med. *5*, 661–674.

Pawlowski, A., Jansson, M., Sköld, M., Rottenberg, M.E., and Källenius, G. (2012). Tuberculosis and HIV co-infection. PLoS Pathog. *8*, e1002464.

Peters, N.C., Egen, J.G., Secundino, N., Debrabant, A., Kimblin, N., Kamhawi, S., Lawyer, P., Fay, M.P., Germain, R.N., and Sacks, D. (2008). *In vivo* imaging

reveals an essential role for neutrophils in leishmaniasis transmitted by sand flies. Science *321*, 970–974.

Picelli, S., Faridani, O.R., Björklund, A.K., Winberg, G., Sagasser, S., and Sandberg, R. (2014). Full-length RNA-seq from single cells using Smart-seq2. Nat. Protoc. *9*, 171–181.

Postat, J., Olekhnovitch, R., Lemaître, F., and Bousso, P. (2018). A Metabolism-Based Quorum Sensing Mechanism Contributes to Termination of Inflammatory Responses. Immunity *49*, 654–665.e5.

Rab, M.A., Hassan, M., Bux, D., Mahmood, M.T., and Evans, D.A. (1992). The isolation and cultivation of *Leishmania infantum* from apparently normal skin of visceral leishmaniasis patients in northern Pakistan. Trans. R. Soc. Trop. Med. Hyg. *86*, 620–621.

Robert, P.A., Jönsson, H., and Meyer-Hermann, M. (2018). MoonFit, a minimal interface for fitting ODE dynamical models, bridging simulation by experimentalists and customization by C++ programmers. bioRxiv. https://doi.org/10.1101/281188.

Rocha, F.J., Schleicher, U., Mattner, J., Alber, G., and Bogdan, C. (2007). Cytokines, signaling pathways, and effector molecules required for the control of *Leishmania* (*Viannia*) *braziliensis* in mice. Infect. Immun. *75*, 3823–3832.

Romano, A., Carneiro, M.B.H., Doria, N.A., Roma, E.H., Ribeiro-Gomes, F.L., Inbar, E., Lee, S.H., Mendez, J., Paun, A., Sacks, D.L., and Peters, N.C. (2017). Divergent roles for Ly6C+CCR2+CX3CR1+ inflammatory monocytes during primary or secondary infection of the skin with the intra-phagosomal pathogen *Leishmania major*. PLoS Pathog. *13*, e1006479.

Runarsson, T., and Yao, X. (2002). Constrained Evolutionary Optimization. In Evolutionary Optimization, R. Sarker, M. Mohammadian, and X. Yao, eds. (Springer US), pp. 87–113.

Scorza, B.M., Carvalho, E.M., and Wilson, M.E. (2017). Cutaneous Manifestations of Human and Murine Leishmaniasis. Int. J. Mol. Sci. *18*, 1296.

Scott, P., and Novais, F.O. (2016). Cutaneous leishmaniasis: immune responses in protection and pathogenesis. Nat. Rev. Immunol. *16*, 581–592.

Segovia-Juarez, J.L., Ganguli, S., and Kirschner, D. (2004). Identifying control mechanisms of granuloma formation during M. tuberculosis infection using an agent-based model. J. Theor. Biol. *231*, 357–376.

Shi, C., and Pamer, E.G. (2011). Monocyte recruitment during infection and inflammation. Nat. Rev. Immunol. *11*, 762–774.

Solomon, M., Pavlotsky, F., Leshem, E., Ephros, M., Trau, H., and Schwartz, E. (2011). Liposomal amphotericin B treatment of cutaneous leishmaniasis due to *Leishmania tropica*. J. Eur. Acad. Dermatol. Venereol. *25*, 973–977.

Späth, G.F., and Beverley, S.M. (2001). A lipophosphoglycan-independent method for isolation of infective *Leishmania* metacyclic promastigotes by density gradient centrifugation. Exp. Parasitol. *99*, 97–103.

Stenger, S., Donhauser, N., Thüring, H., Röllinghoff, M., and Bogdan, C. (1996). Reactivation of latent leishmaniasis by inhibition of inducible nitric oxide synthase. J. Exp. Med. *183*, 1501–1514.

Sugimoto, M.A., Sousa, L.P., Pinho, V., Perretti, M., and Teixeira, M.M. (2016). Resolution of Inflammation: What Controls Its Onset? Front. Immunol. *7*, 160.

Terrazas, C., Varikuti, S., Oghumu, S., Steinkamp, H.M., Ardic, N., Kimble, J., Nakhasi, H., and Satoskar, A.R. (2017). Ly6C^{hi} inflammatory monocytes promote susceptibility to *Leishmania donovani* infection. Sci. Rep. *7*, 14693.

Thomas, D.D., Liu, X., Kantrow, S.P., and Lancaster, J.R., Jr. (2001). The biological lifetime of nitric oxide: implications for the perivascular dynamics of NO and O2. Proc. Natl. Acad. Sci. USA *98*, 355–360.

van Zandbergen, G., Bollinger, A., Wenzel, A., Kamhawi, S., Voll, R., Klinger, M., Müller, A., Hölscher, C., Herrmann, M., Sacks, D., et al. (2006). *Leishmania* disease development depends on the presence of apoptotic promastigotes in the virulent inoculum. Proc. Natl. Acad. Sci. USA *103*, 13837–13842.

Walker, D.M., Oghumu, S., Gupta, G., McGwire, B.S., Drew, M.E., and Satoskar, A.R. (2014). Mechanisms of cellular invasion by intracellular parasites. Cell. Mol. Life Sci. *71*, 1245–1263.

Wilhelm, P., Ritter, U., Labbow, S., Donhauser, N., Röllinghoff, M., Bogdan, C., and Körner, H. (2001). Rapidly fatal leishmaniasis in resistant C57BL/6 mice lacking TNF. J. Immunol. *166*, 4012–4019.

Zhao, G., Wirth, D., Schmitz, I., and Meyer-Hermann, M. (2017). A mathematical model of the impact of insulin secretion dynamics on selective hepatic insulin resistance. Nat. Commun. *8*, 1362.

STAR★METHODS

KEY RESOURCES TABLE

REAGENT or RESOURCE	SOURCE	IDENTIFIER
Antibodies		
anti-CD18	BD Biosciences	Cat# 555280; RRID: AB_395703
anti-CD49d	BioXcell	Cat# BE0071; RRID: AB_1107657
anti-CD16/32	BioLegend	Cat# 101320; RRID: AB_1574975
anti-NOS2	SantaCruz	Cat# sc-650-G;RRID: AB_631832
Goat IgG	Jackson ImmunoResearch	Cat# 005-000-003; RRID: AB_2336985
APC-anti-iNOS	eBioscience	Cat# 17-5920-82; RRID: AB_2573244
APC-anti-pro-IL1β	eBioscience	Cat# 17-7114-80; RRID: AB_10670739
DyeLight 649 anti-goat	Jackson ImmunoResearch	Cat# 205-492-146; RRID: AB_2339069
Ly6G-Brilliant Violet 421	BioLegend	Cat# 127601; RRID: AB_1089179
MHCII (I-A/I-E)-Brilliant Violet 510	BioLegend	Cat# 107635; RRID: AB_2561397
CD45-PerCP/Cy5.5	BioLegend	Cat# 103131; RRID: AB_893344
CD11b-APC/Cy7	BioLegend	Cat# 101225; RRID: AB_830641
CD4-AF488	BioLegend	Cat# 100532; RRID: AB_493373
CD11c-APC	BioLegend	Cat# 117309; RRID: AB_313778
CD45-PE	BioLegend	Cat# 103105; RRID: AB_312970
CD3e-Brilliant Violet 421	BioLegend	Cat# 100341; RRID: AB_2562556
CD11b-Brilliant UV395	BD Biosciences	Cat# 563553; RRID: AB_2738276
CD45-APC/Cy7	BioLegend	Cat# 103115; RRID: AB_312980
Ly6C-PE/Cy7	BioLegend	Cat# 128017; RRID: AB_1732093
CD45.2-APC/Fire750	BioLegend	Cat# 109851; RRID: AB_2629722
Ly6C-APC/Cy7	BioLegend	Cat# 128025; RRID: AB_10643867
Ly6G-APC/Cy7	BioLegend	Cat# 127623; RRID: AB_10645331
Ly6G-APC	BioLegend	Cat# 127613; RRID: AB_1877163
TER119	BioLegend	Cat# 116254; RRID: AB_2832400
CD5	BioLegend	Cat# 100602; RRID: AB_312731
CD45R	BioLegend	Cat# 103270; RRID: AB_2832305
F4/80	BioLegend	Cat# 123164; RRID: AB_2888757
CD117	BioLegend	Cat# 135114; RRID: AB_2561477
CD49b	BioLegend	Cat# 103526; RRID: AB_2888793
Chemicals, peptides, and recombinant proteins		
M199 medium	Sigma-Aldrich	Cat# FG0615
FCS	Biowest	Cat# S1400
Adenine	Sigma-Aldrich	Cat# A8626; CAS Number 73-24-5
Biotin	Sigma-Aldrich	Cat# B4501; CAS Number 58-85-5
Hemin	Sigma-Aldrich	Cat# H9039; CAS Number 16009-13-5
Biopterin	Sigma-Aldrich	Cat# B2517; CAS Number 22150-76-1
Peanut agglutinin	Vector Laboratories	Cat# L-1070
Ketamine	Ratiopharm	ATC Code N01AX03
Rompun	Bayer	ATC Code N05CM92
Acepromazin	CEVA GmbH	ATC Code QN05AA04
AmBisome	Gilead Sciences	ATC Code J02AA01
L-NIL	Sigma-Aldrich	Cat# I8021; CAS Number 159190-45-1
DNFB	Sigma Aldrich	Cat# D1529; CAS Number 70-34-8
Olive oil	Sigma-Aldrich	Cat#O1514; CAS Number 8001-25-0

(*Continued on next page*)

CellPress
OPEN ACCESS

Continued

REAGENT or RESOURCE	SOURCE	IDENTIFIER
Penicillin/ Streptomycin	GIBCO/ThermoFisher	Cat# 15140122
Liberase TL	Roche/Sigma-Aldrich	Cat# 5401020001
DNase I	ThermoFisher/Invitrogen	Cat# 18047-019
Paraformaldehyde	Sigma-Aldrich	Cat# 158127; CAS Number 30525-89-4
Perm/Wash buffer	BD Biosciences	Cat# 554723
RPMI 1640	Pan Biotech	Cat# P04-18500
GolgiPlug	BD Biosciences	Cat# 555029
LPS	Sigma-Aldrich	Cat# L8274; EC Number 297-473-0
IFN-γ	R&D Systems	Cat# 485-MI
Seahorse XF DMEM medium	Agilent	Cat# 103575-100
RNeasy Plus Micro Kit	QIAGEN	Cat# 74034
SUPERase-In RNase-Inhibitor	ThermoFisher/Invitrogen	Cat# AM2694
SYBR Green PCR Master Mix	ThermoFisher/Applied Biosystems	Cat#4309155
Carbonylcyanid-3-chlorphenylhydrazon (CCCP)	Sigma-Aldrich	Cat# C2759; CAS Number 555-60-2
RPMI 1640 without phenol red	GIBCO/ThermoFisher	Cat# 11835030
GlutaMax	GIBCO/ThermoFisher	Cat# 35050061
LysoSensor Green DND-189	ThermoFisher	Cat# L7535
BamHI	New England Biolabs	Cat# R0136
KpnI	New England Biolabs	Cat# R0142
BglII	New England Biolabs	Cat# R0144
BfrI	New England Biolabs	Cat# R0520
SwaI	New England Biolabs	Cat# R0604
Hygromycin B	ThermoFisher	Cat# 10687010; CAS Number 31282-04-9
Calf intestinal alkaline phosphatase	New England Biolabs	Cat# M0290
Critical commercial assays		
Monocyte Isolation Kit (BM)	Miltenyi Biotec	Cat# 30-100-629
CD11c MicroBeads	Miltenyi Biotec	Cat# 130-108-338 and 130-125-835
MitoStress Test Kit	Agilent	Cat# 103707-100
CountBright Absolute Counting beads	ThermoFisher	Cat# C36950
Agencourt® RNAClean XP beads	Beckman Coulter	Cat# A63987
Experimental models: Organisms/strains		
C57BL/6J mouse line	Charles River	Strain Code 027
B6.SJL-*Ptprc*^{a}*Pepc*^{b}/BoyJ mouse line	Charles River	Strain Code 494
B6.129S7-*Rag1*^{tm1Mom} mouse line	The Jackson Laboratory	Stock Number #002216
Tg(CAG-KikGR)33Hadj/J mouse line	The Jackson Laboratory	Stock Number #013753
L. major LRC-L137 V121 wild-type	(Handman et al., 1983)	N/A
L. major LRC-L137 V121, dsRed expressing	(Misslitz et al., 2000)	N/A
L.major^{SWITCH}	(Müller et al., 2013)	N/A
L. major^{necR}	This paper	N/A
Oligonucleotides		
mNectarine forward primer and linker AGT GGATCCGACGAGATGGAGCAGAAGCTG ATCTCGGAGGAGGACCTGAAGgtgagcaa gggcgaggag	Eurofins	N/A
mNectarine reverse primer and linker GCTG GTACCATCGACCTCATCTCCGCTGAGCT CGGACGAGGActtgtacagctcgtccatgc	Eurofins	N/A
pro-IL1beta forward primer TACCT GTGTCTTTCCCGTGG	Eurofins	N/A

(*Continued on next page*)

Continued		
REAGENT or RESOURCE	SOURCE	IDENTIFIER
pro-IL1beta reverse primer AGCAG GTTATCATCATCATCCCA	Eurofins	N/A
HPRT1 forward primer CAGGC CAGACTTTGTTGGAT	Eurofins	N/A
HPRT1 reverse primer GGCTT TGTATTTGGCTTTTCC	Eurofins	N/A
Recombinant DNA		
L. major^{necR} targeting vector	This paper	N/A
pBAD-mNectarine	Addgene	Acc. Number 21717
pLEXSY-hyg2	Jena Bioscience	Cat# EGE-232
pcDNA3.1-CFP-DEVG-YFP	(Breart et al., 2008)	N/A
Software and algorithms		
BD FACSDiva	BD Biosiences	Version 8.0.2
NxT Software	ThermoFisher	Version 4.2
FlowJo	LLC, BD Biosiences	Version 10.6.2
ZEN	Zeiss	Version 2.1
Imaris	Bitplane, Perkin Elmer	Versions 9.3.1. through 9.5.1
MoonFit	(Robert et al., 2018)	N/A
Fiji	ImageJ	Version 1.51 s
DiscIT	(Moreau et al., 2012)	N/A
cellR	Olympus	Version 2.0
Other		
ibiTreat µSlide I 0.8 Luer	Ibidi	Cat# 80196
poly-L-lysine coated µ-Slide VI 0.4	Ibidi	Cat# 80606

RESOURCE AVAILABILITY

Lead contact

Further information and requests for resources and reagents should be directed to and will be fulfilled by the lead contact, Andreas J. Müller (andreas.mueller@med.ovgu.de).

Materials availability

All *L. major* strains and integration vectors generated in this study will be made available on request by the lead contact with a completed Materials Transfer Agreement.

Data and code availability

All data reported in this paper, and any additional information required to reanalyze the data, will be shared by the lead contact upon request. All unpublished code employed for mathematical modeling was deposited under https://github.com/AnastasiosSiokis/Leishmania.

EXPERIMENTAL MODEL AND SUBJECT DETAILS

Mice

All mice used in this study were maintained on the C57BL/6 genetic background. C57BL/6 and CD45.1 wt (B6.SJL-*Ptprc*^{a}*Pepc*^{b}/BoyJ) mice were bred in the Central Animal Laboratory (ZTL) of the Medical Faculty of Otto-von-Guericke-University Magdeburg, or purchased from Charles River (Sulzfeld, Germany). *Rag1*$^{-/-}$ (B6.129S7-*Rag1*^{tm1Mom}, kindly provided by Thomas Schüler, University of Magdeburg) and photoconvertible mKikume-expressing mice (Tg(CAG-KikGR)33Hadj/J were purchased from Jaxmice (Bar Harbor, MA)) and bred in the ZTL. All mice were housed under SPF conditions, sex- and age-matched animals between 7 and 14 weeks were used for infections. All animal experiments were reviewed and approved by the Ethics Committee of the Office for Veterinary Affairs of the State of Saxony-Anhalt, Germany (permit license numbers IMKI/G/03-1253/14, IMKI/G/01-1314/15 and IMKI/G/01-1575/19) in accordance with legislation of both the European Union (Council Directive 499 2010/63/EU) and the Federal Republic of Germany (according to § 8, Section 1 TierSchG, and TierSchVersV).

Pathogen

L. major LRC-L137 V121 wild-type (Handman et al., 1983), dsRed or mKikume expressing *L.major*^{SWITCH} parasites (Misslitz et al., 2000; Müller et al., 2013) were grown in M119 medium supplemented with 10% heat-inactivated fetal calf serum, 0.1 mM adenine, 1 mg/ml biotin, 5 mg/ml hemin, and 2 mg/ml biopterin (all from Sigma-Aldrich) at 26 C° for a maximum of 6 passages. The *L. major*^{necR} targeting vector for expression of a CFP-mNectarine fusion construct was generated as follows: The mNectarine gene was amplified from the pBAD-mNectarine plasmid (Johnson et al., 2009) (Addgene accession number 21717) using AGTGGATCCGACGAGATG GAGCAGAAGCTGATCTCGGAGGAGGACCTGAAGGTGAGCAAGGGCGAGGAG forward and GCTGGTACCATCGACCTCATCTCC GCTGAGCTCGGACGAGGACTTGTACAGCTCGTCCATGC for insertion of linker sequences, and cloned via BamHI and KpnI (New England Biolabs) into a pcDNA3.1-CFP-DEVG-YFP expression vector (Breart et al., 2008) to replace in frame YFP with mNectarine in the fusion construct. The resulting CFP-DEVG-mNectarine fusion protein gene was cloned via BamHI/BglII and BfrI (New England Biolabs) into a pLEXSY-hyg2 vector (Jena Bioscience), targeting an rDNA locus of *L. major* LRC-L137 V121. The resulting plasmid was linearized using SwaI (New England Biolabs), dephosphorylated using alkaline phosphatase (New England Biolabs) and electroporated into *L. major*. Stable transfectants were selected with 30 mg/ml hygromycin B (ThermoFisher). Single clones were obtained by limiting dilution.

Bone marrow-derived monocytes

Monocytes were isolated from bone marrow via negative magnetic selection using the Monocyte Isolation Kit (BM) (Miltenyi Biotec). The cells were incubated for 24h in RPMI (10% FCS, 5% P/S) in a 24 well plate (10^6 cells/ml/well). In order to activate the monocytes, LPS (1μg/ml) and INF-γ (10 ng/ml) were added to the designated wells.

Bone marrow-derived neutrophils

Bone marrow cells from C57BL/6 mice were filtered through a 70-μm cell strainer in PBS and neutrophils were isolated by negative MACS selection (Miltenyi Biotec) as described previously (Hasenberg et al., 2011) using biotinylated anti-TER119 (clone TER119, 1 μg/ml), anti-CD5 (clone 53-7.3, 2.5 μg/ml), anti-CD45R (clone RA3-6B2, 1.25 μg/ml), anti-F4/80 (clone BM8, 5 μg/ml), anti-CD117 (clone 2B8, 1 μg/ml) and anti-CD49b (clone HMa2, 2.5 μg/ml) antibodies (all from Biolegend).

Peritoneal macrophages

Peritoneal macrophages were obtained by two sequential i.p. injections and aspirations of 5 mL ice-cold PBS into untreated C57BL/6 mice post mortem.

METHOD DETAILS

In vivo infections

For low dose infection, metacyclic parasites were prepared from stationary-phase cultures by lectin agglutination (da Silva and Sacks, 1987). For this, cultures were concentrated 10-fold by centrifugation, incubated at room temperature for 20 min in the presence of 50 μg/ml peanut agglutinin (Vector Laboratories, #L-1070) and spun at 100 g for 10 min. Non-agglutinated metacyclics were recovered from the supernatant, washed three times, enumerated and resuspended in PBS at a concentration of $5x10^3$ parasites per μl. For infection, mice were lightly anesthetized with a mixture of ketamine (Ketamin-ratiopharm, 70 mg/kg body weight) and xylazine (Rompun 2%, Bayer, 7 mg/kg body weight) and inoculated into each ear pinna with 1 μl of parasite suspension using a microsyringe (NanoFil, World Precision Instruments) mounted with a 35G-beveled needle. For high dose infection of mice, stationary phase cultures were centrifuged (1000 g, 10 min, RT) and resuspended for injection in 5 μl PBS. $2x10^6$ (for flow cytometry and metabolic flux experiments) or $5x10^5$ (for IV-2PM) parasites were subsequently injected into the ear dermis.

In vitro infections

For pH characterization of the parasitophorous vacuole environment, *in vitro* infections were performed with purified DsRed-expressing *L. major* metacyclic promastigotes at a multiplicity of infection of 5:1 for macrophages (with washing of the adherent cells after 2h p.I.) and 2:1 for neutrophils (without washing), in RPMI1640 without phenol red (GIBCO) supplemented with 10% FCS, 1% Penicillin/Streptomycin and 1% GlutaMax in poly-L-lysine coated microslides (μ-Slide VI 0.4; Ibidi, Munich, Germany). To stain the acidified lysosomal compartment, LysoTracker Green DND-189 (ThermoFisher) was added to a final concentration of 1 μM to the culture, and cells were further incubated for 15 min at 37°C and 5% CO_2 before imaging. To determine the LysoSensor staining around the parasites, fluorescence profile plots were obtained using the ImageJ software. As a negative control, the protonophore carbonyl cyanide CCCP (Sigma-Aldrich) was additionally added to a final concentration of 1 mM to the cell culture before incubation with LysoSensor Green (ThermoFisher). Cells were imaged 24h after infection using an Olympus BX61 microscope controlled by the cellR software (Version 2.0, Olympus Biosystems) and equipped with a 20x objective. A mercury arc lamp served as illumination source and was combined with excitation filters at 430/24 nm and 556/20 nm, a FITC/Cy3/Cy5 triple-band dichroic beamsplitter and emission filters at 520/28 nm and 617/73 nm. For characterization of the death reporter *in vitro*, peritoneal macrophages were seeded in microslides (ibiTreat μSlide I 0.8 Luer, Ibidi) in RPMI 1640 GlutaMax medium (GIBCO) supplemented with 10% FCS and 1% Penicillin/Streptomycin. Cells were allowed to adhere for 6 hours prior to infection at a ratio of 1:5 with purified *L. major*^{necR} and *L. major*^{SWITCH} metacyclic promastigotes opsonized for 20 min at room temperature with 4% naive mouse serum. Cells were washed 2 hours after

infection and incubated for 24 hours at 37°C and 5% CO_2 before macrophage activation with IFNγ (10 ng/ml, R&D Systems) and LPS (1 μg/ml, *E. coli* O26:B6, Sigma-Aldrich). Optionally, iNOS was inhibited by addition of N6-(1-iminoethyl)-L-lysine hydrochloride (L-NIL) (23 μg/ml, Sigma-Aldrich). Cells were imaged 48 hours after infection for over 40 hours at a rate of 6 images/h using an Olympus BX61 microscope controlled by the cellR software (Olympus Biosystems) and equipped with a 20x objective. A mercury arc lamp served as illumination source and was combined with excitation filters at 387/11 nm, 480/17 nm and 556/20 nm, a DAPI/FITC/TRITC triple-band dichroic beamsplitter and emission filters at 470/24 nm, 520/28 nm and 617/73 nm. The green signal from *L. major*^{SWITCH} parasites was used by the autofocus module of the system to keep infected cells in focus. Movies were analyzed using Fiji (ImageJ Version 1.51 s, http://imageJ.nij.gov/ij). Parasites were manually tracked and their CFP and mNectarine fluorescence content as well as background fluorescence were measured over time. After background subtraction, values from both channels were normalized by the corresponding average value measured over the first hour of recording. Parasites from LNIL-treated cultures (n = 11) were used as controls to determine the 95% confidence interval within which CFP and mNectarine fluorescence values of viable parasites are expected to lie. The detection span of the death reporter was defined as the time between the decrease of mNectarine and CFP fluorescence below their respective lower confidence interval limit.

Parasite pH manipulation *in vitro*

100 μL of an exponentially-growing culture of the *L. major*^{necR} strain were harvested, washed with PBS adjusted at pH = 4.5, 6 or 7 and incubated for 10 min with either 10 μM CCCP (Sigma-Aldrich) or 0.01% DMSO in buffered PBS before image acquisition. Images were acquired using a 40x objective mounted on an Olympus BX61 microscope controlled by the cellR software (Version 2.0, Olympus Biosystems). A mercury arc lamp served as illumination source and was combined with excitation filters at 430/24 nm and 556/20 nm, a ECFP/EYFP/DsRed triple-band dichroic beamsplitter and emission filters at 470/24 nm and 617/73 nm. Using Fiji (ImageJ Version 1.51 s, http://imageJ.nij.gov/ij), parasite outlines were extracted from phase contrast images and the resulting ROIs were then applied to fluorescence images to measure the CFP and mNectarine fluorescence signals of individual parasites. Background fluorescence values from both channels were measured in a 5-pixel band surrounding each parasite ROI and were subtracted from values measured within parasite ROIs.

iNOS inhibition and blocking of cell recruitment

To inhibit iNOS activity, L-N^6-(1-Iminoethyl)lysine dihydrochloride (L-NIL, Sigma-Aldrich) was freshly prepared at a concentration of 2 mg/mL in PBS and mice were daily injected with 200 μg intraperitoneally (i.p.) for 3 days, starting 18 days p.i. To block leukocytes extravasation, mice were injected with 200 μg anti-CD18 (BD Biosciences) and 200 μg anti-CD49d (BioXcell) i.p. once on day 18 p.i.

AmBisome treatment

To induce parasite death and assess the functionality of the viability reporter strain *in vivo*, mice infected with *L. major*^{necR} metacyclic parasites were treated with AmBisome (Gilead Sciences), starting 15 days p.i.. AmBisome was reconstituted at 4 mg/ml with sterile water for injection and then mixed 1:1 with 5% dextrose prior to treatment. Mice were injected daily with 25 mg/kg/day administered intraperitoneally for 4 to 6 days prior to IV-2PM or parasite load determination. Control animals were treated with the water/sucrose vehicle solution.

Monocyte deposition

Monocytes were incubated for 24h after activation with LPS (1μg/ml) and INF-γ (10 ng/ml) or mock-treatment. The cells were centrifuged (450 g, 8 min, 4°C) and injected in 10 μl PBS into the ear dermis 3 days prior to analysis by flow cytometry and limiting dilution analysis of pathogen burden.

Irritant contact dermatitis

To induce contact dermatitis, a single dose of 20 μl of 0.5% 2,4-dinitrofluorobenezene (DNFB, Sigma-Aldrich) in acetone/olive oil (4:1) was applied epicutaneously onto the infected ear. Vehicle controls were treated with the solvent only. Mice were analyzed 3 days after treatment.

Determination of ear swelling

Thickness of the infected ears was determined using a Mitutoyo 7301 caliper. For this, the mice were anesthetized with isoflurane and the center of caliper measurement area (10 mm diameter) was placed on the border of the ear pinna, thus covering a 5 mm diameter lens-shaped area of the outer part of the ear including the site of infection. Measurements were done in a blinded manner.

Extraction of leukocytes from infected ears

Infected ears harvested from euthanized mice were separated into dorsal and ventral sheets using tissue forceps before being digested for 60 min at 37 C° while shaking at 600 rpm in RPMI medium 1640 supplemented with 100 U/ml Penicillin/Streptomycin (GIBCO), 0.5 mg/ml liberase TL (Roche), and 50 ng/ml DNase (Sigma-Aldrich). Single cell suspensions in PBS were prepared by crushing digested ears through a 70 μm cell strainer. After washing the cells suspension once with PBS once, dermal cells were analyzed by flow cytometry or employed for limiting dilution assay.

Limiting dilution assay

Quadruplicate samples of each ear homogenate were serially diluted in 1:2 steps in *Leishmania* culture medium in 96-well plates. After 14 days at 26°C, the highest dilution which exhibited parasite growth was identified for each replicate and the initial parasite number was defined as the mean value of the replicates, calculated back to the original volume of the ear homogenate.

Flow cytometry

For characterization of the immune infiltrate and iNOS expression at the site of infection, single cell suspensions obtained from digested ears were fixed for 1h at 4°C in 2% PFA, permeabilized for 15 min at RT with 1x Perm/Wash buffer (BD Biosciences) and blocked for 15 min at RT with anti-CD16/32 antibody (clone 93, 10 μg/ml). For analysis of pro-IL1β, cells were incubated for 4h at 37°C in RPMI (10% FCS, 5% P/S) containing 0.1% GolgiPlug in a 24 well plate prior to fixation. Samples were then incubated for 30 min at RT in Perm/Wash buffer with anti-NOS2 antibody (goat polyclonal, 1 μg/ml) or control IgG (goat, 1 μg/ml) for low dose infection, and directly APC-labeled anti-iNOS antibody (Clone CXNFT, 2 μg/ml) for high dose infections, or APC-labeled anti-pro-IL1β (Clone NJTEN3, 1 μg/ml). Secondary staining of non-labeled primary antibody was revealed with DyeLight 649 anti-goat secondary antibody (7 μg/ml) incubated for 45 min at RT (iNOS) or 4°C (pro-IL1β) in Perm/Wash buffer. Surface staining was performed for 20 min at 4°C with a selection from the following antibodies: Ly6G-Brilliant Violet 421 (clone 1A8, 0.5 μg/ml), MHCII (I-A/I-E)-Brilliant Violet 510 (clone M5/114.15.2, 2.5 μg/ml), CD45-PerCP/Cy5.5 (clone 30-F11, 0.5 μg/ml), CD11b-APC/Cy7 (clone M1/70, 0.5 μg/ml), CD4-AF488 (clone RM4-5, 2.5 μg/ml), CD11c-APC (clone N418, 1 μg/ml), CD45-PE (clone 30-F11, 0.5 μg/ml), CD3e-Brilliant Violet 421 (Clone 145-2C11, 0.5 μg/ml). For staining of non-fixed cells, selections from the following antibodies were used additionally: CD11b-Brilliant UV395 (Clone M1/70, 2 μg/ml), CD45-APC/Cy7 (clone 30-F11, 2 μg/ml), Ly6C-PE/Cy7 (Clone HK 1.4, 2 μg/ml), CD45.2-APC/Fire 750 (Clone 104, 1 μg/ml), Ly6C-APC/Cy7 (Clone HK 1.4, 2 μg/ml), Ly6G-APC/Cy7 (Clone 1A8, 0.5 μg/ml), and Ly6G-APC (Clone 1A8, 0.5 μg/ml).

Analysis was performed with a Fortessa (BD Biosciences) flow cytometer using the BD FACSDiva software or an Attune NxT flow cytometer using the NxT Software. Cytometers were equipped with excitation lasers at 395nm, 405 nm, 488 nm, 561 nm, and 633 nm. For calculation of absolute cell numbers, $5x10^4$ CountBright Absolute Counting beads were added to the samples before measurement. Data were analyzed with the FlowJo software as exemplified in Figures S1D and S1E.

Photoconversion

Mice anaesthetized with 100 mg/kg ketamine and 10 mg/kg xylazine i.p. were photoconverted on the ear (ventral side) with violet light at 405 nm wavelength using an assembly of 2 × 2 LED diodes (Strato, half-viewing angle: 15°; Radiant Power: 10 mW), illumination time was set to 2 minutes at a distance of 2 cm. Before and after photoconversion of the parasites, a three-dimensional image of the site of infection spanning at least 500 μm x 500 μm x 30 μm was acquired by using IV-2PM. The readout image of the same site was localized 48h later using triangulation as described previously (Müller et al., 2013). To analyze the dynamics of host cell recruitment at the site of infection (Heyde et al., 2018), mKikume-expressing mice infected with nonfluorescent *L. major* were photoconverted using the same procedure 4 days before analysis (i.e., day 17 p.i.) by flow cytometry. For flow cytometry of mKikume-expressing mouse cells, non-photoconverted mKikume was acquired using 488 nm excitation and 530/30 nm emission, photoconverted mKikume was detected using 561 nm excitation and 610/10 nm emission.

Intravital 2-photon microscopy (IV-2PM)

Mice were injected with 100 mg/kg ketamine and 10 mg/kg xylazine i.p., and were supplemented with 3 mg/kg Acepromazin i.p. after the onset of anesthesia for mKikume imaging. The ventral side of the ear was prepared for IV-2PM as described (Müller et al., 2013). In brief, the animals were placed on a heating stage adjusted to 37 °C, their ear was fixed to a metal platform using double-sided tape and was covered with Vidisic carbomer gel (Bausch+Lomb). A coverslip sealed to a surrounding parafilm blanket was placed onto the gel and fixed above the ear. 2-photon imaging was performed with a W Plan-Apochromat 20x/1,0 DIC VIS-IR objective using a Zeiss LSM 700 upright microscope with the ZEN software environment (Version 2.1, Zeiss), equipped with a Mai Tai DeepSee Ti:Sa laser (Spectra-Physics) tuned to 920 or 980 nm to image mKikume expressing Lm^{SWITCH}, with the emitted signal being split by 465 nm, 520 nm, 555 nm, and 620 nm long pass dichroic mirrors and filtered with 509/22 nm (green mKikume, 520 nm part), 543/20 nm (green mKikume, 540 nm part), 589/54 nm (red mKikume, mNectarine), 660/80 nm (far red autofluorescence) and SHG unfiltered below 465 nm. Excitation was tuned to 920 nm to excite CFP-mNectarine expressing *L. major*^{necR}, with the emitted signal was split by 465 nm, 490 nm, 520 nm, and 555 nm long pass dichroic mirrors and filtered with 485 nm short pass (CFP below 485 nm designated as CFP490), 509/22 nm (CFP, above 498 nm designated CFP520) and 589/54 nm band pass (red mNectarine) filters (SHG unfiltered below 465 nm) before collection with non-descanned detectors.

Metabolic analyses

Single cell suspensions were prepared from infected ears at 3 wpi as described above. For MACS purification of inflammatory monocyte-derived cells, CD11c MicroBeads (Miltenyi Biotec) were employed according to the manufacturer's instructions, as it was previously shown that inflammatory monocyte-derived cells express this marker (Heyde et al., 2018; Postat et al., 2018). The isolated cells were counted and pooled from several infected ears to reach constant cell number within each experiment of $6 – 8 x10^4$ cells per well, which were plated in XFe96 cell culture plates coated with Cell-Tak (Fisher Scientific). Seahorse XF DMEM medium was supplemented with 10 mM glucose, 2 mM glutamine and 1mM pyruvate with an adjusted pH of 7.4. Metabolic analysis was performed by analyzing

each sample using an XFe96 Extracellular Flux Analyzer (Seahorse Bioscience). According to the manufacturer's instructions, baseline ectracellular acificication rate (ECAR) and oxygen consumption rate (OCR) was measured untreated cells, and OCR was additionally determined in response to 1 μM oligomycin, 1.5 μM fluorocarbonyl cyanide phenylhydrazone (FCCP) and 0.5 μM rotenone plus antimycin A (MitoStress Test Kit, Agilent). Maximal respiration and spare respiratory capacity were calculated by monitoring OCR. For normalization to 10^5 cells between different experiments, we made use of the direct correlation between measured metabolic flux rates and plated cell numbers (Janssen et al., 2021).

Quantitative PCR analysis

1000 cells/sample were taken from MACS-purified inflammatory monocyte-derived cells (CD11c MicroBeads, see above) lysed in 10μl lysis buffer (RNeasy Plus lysis buffer, QIAGEN) containing 5% SUPERase-In RNase-Inhibitor 20U/μl (Invitrogen). RNA from lysed cells was purified using Agencourt® RNAClean XP beads (Beckman Coulter) according to manufacturer's instruction. Reverse transcription and cDNA clean-up was performed using the Smart-seq2 protocol as previously described (Picelli et al., 2014). Primers for pro-IL-1β (5′-TACCTGTGTCTTTCCCGTGG-3′ and 5′-AGCAGGTTATCATCATCATCCCA_3′) and HPRT1 as a housekeeping gene (5′-CAGGCCAGACTTTGTTGGAT-3′ and 5′-GGCTTTGTATTTGGCTTTTCC-3′) were used for quantitative PCR analysis in a SYBR Green PCR Master Mix (Applied Biosystems). To run the quantitative PCR, 40 cycles of 95°C (15 s), and 60°C (60 s) followed by a dissociation protocol 95°C (15 s), 60°C (20 s), and 95°C (15 s) were performed on a ABI Prism® 7000 Sequence detection system (Applied Biosystems). For each samples, technical duplicates were performed. Samples which exhibited a too low cDNA concentration for the housekeeping gene ($c_t > 26$) were excluded from the analysis.

Mathematical models and model selection

$$\frac{dM(t)}{dt} = k_{\mathrm{Mrec}} + \frac{aP(t)}{S_{\mathrm{M}} + P(t)} - k_{\mathrm{i}}P(t)M(t) - k_{\mathrm{a}}\frac{D(t)}{C_{\mathrm{im}} + M_{\mathrm{a}}(t) + M_{\mathrm{ai}}(t)}M(t) - d_{\mathrm{M}}M(t) \quad \text{(Equation 1)}$$

$$\frac{dM_{\mathrm{i}}(t)}{dt} = k_{\mathrm{i}}P(t)M(t) - k_{\mathrm{ai}}\frac{D(t)}{C_{\mathrm{im}} + M_{\mathrm{a}}(t) + M_{\mathrm{ai}}(t)}M_{\mathrm{i}}(t) - d_{\mathrm{M_i}}M_{\mathrm{i}}(t) \quad \text{(Equation 2)}$$

$$\frac{dM_{\mathrm{ai}}(t)}{dt} = k_{\mathrm{ai}}\frac{D(t)}{C_{\mathrm{im}} + M_{\mathrm{a}}(t) + M_{\mathrm{ai}}(t)}M_{\mathrm{i}}(t) + k_{\mathrm{ia}}P(t)M_{\mathrm{a}}(t) - d_{\mathrm{M_{ai}}}M_{\mathrm{ai}}(t) \quad \text{(Equation 3)}$$

$$\frac{dM_{\mathrm{a}}(t)}{dt} = k_{\mathrm{a}}\frac{D(t)}{C_{\mathrm{im}} + M_{\mathrm{a}}(t) + M_{\mathrm{ai}}(t)}M(t) - k_{\mathrm{ia}}P(t)M_{\mathrm{a}}(t) - d_{\mathrm{M_a}}M_{\mathrm{a}}(t) \quad \text{(Equation 4)}$$

$$\frac{dD(t)}{dt} = k_{\mathrm{D}}P(t)[1 - D(t)]D(t) \quad \text{(Equation 5)}$$

Six ordinary differential equation (ODE) models representing different hypotheses on the mode of *L. major* control were formulated. All models take into account the population dynamics for (1) non-infected iNOS⁻ (M), (2) infected iNOS⁻ (M_i), (3) non-infected iNOS⁺ (M_a) and (4) infected iNOS⁺ (M_{ai}) monocyte-derived phagocytes, where iNOS⁺ denotes activation. The models differ in the mode of parasite burden (P) control.

Upon infection by P, basal recruitment at the site of infection, k_{Mrec}, as well as recruitment of monocytes due to the parasite burden, $\frac{aP(t)}{S_{\mathrm{M}} + P(t)}$, are considered (Equation 1). The newly recruited monocyte-derived phagocytes, M, either phagocytose or are actively invaded by P, resulting in their infection (M_i) with rate k_i, $k_iP(t)M(t)$ (Equations 1 and 2). Furthermore, monocyte-derived phagocytes residing at the site of infection interact with newly recruited Th1 cells, resulting in their activation, shown by iNOS⁺. Since the scope of the model is to understand the effect of monocyte-derived phagocytes, the population of Th1 cells and their interactions with monocytes is not explicitly modeled. Instead, the time interval between infection and Th1 presence is modeled with a sigmoid as a delay function, D, with rate k_D and is dependent on the parasite burden, P:$k_DP(t)[1-D(t)]D(t)$ (Equation 5). Non-infected (M) as well as infected (M_i) monocyte-derived phagocytes get activated with rates k_a and k_{ai}, following $k_a\frac{D(t)}{C_{\mathrm{im}} + M_{\mathrm{a}}(t) + M_{\mathrm{ai}}(t)}M(t)$ and $k_{ai}\frac{D(t)}{C_{\mathrm{im}} + M_{\mathrm{a}}(t) + M_{\mathrm{ai}}(t)}M_i$, respectively (Equations 1–4). This leads to the appearance of non-infected iNOS⁺ (M_a) and infected iNOS⁺ (M_{ai}) monocyte-derived phagocytes. Additionally, M_a get infected with rate k_{ia}, $k_{ia}P(t)M_a(t)$. Natural death of all four monocyte populations is taken into consideration, $d_XX(t)$, where X = M, M_i, M_{ai}, M_a (Equations 1–4).

In order to understand the mode of *L. major* control and separate between direct killing, proliferation inhibition or a combination of both, as well as between cell-intrinsic and extrinsic control, six hypotheses were formulated and tested.

CellPress
OPEN ACCESS

Cell-intrinsic properties

In model 1, we hypothesized that parasites proliferate in both infected iNOS⁻ and iNOS⁺ monocyte-derived phagocytes, with the same rate k_P,$k_P\left(1-\frac{P(t)}{C_P}\right)P(t)[M_i(t)+M_{ai}(t)]$. Additionally, parasites are actively killed only by infected iNOS⁺ monocytes, with rate k_r, $k_rP(t)M_{ai}(t)$, and undergo natural death, with rate d_P, $d_PP(t)$ (Equation 6a). In the second model, active parasite killing is absent, but instead, there is the possibility of impaired parasite proliferation in infected iNOS⁺ monocytes, $k_P\left(1-\frac{P(t)}{C_P}\right)P(t)[M_i(t)+\beta M_{ai}(t)]$, where $\beta\in[0,1]$ (Equation 6b), and $\beta=0$ represents complete parasite proliferation inhibition whereas $\beta=1$, denotes normal proliferation in infected iNOS⁺ monocytes (Equation 6b). The third model is a combination of the previous two models, i.e., impaired parasite proliferation together with active killing by infected iNOS⁺ monocyte-derived phagocytes (Equation 6c).

$$\frac{dP(t)}{dt}=k_P\left(1-\frac{P(t)}{C_P}\right)P(t)[M_i(t)+M_{ai}(t)]-k_rP(t)M_{ai}(t)-d_PP(t) \quad \text{(Equation 6a)}$$

$$\frac{dP(t)}{dt}=k_P\left(1-\frac{P(t)}{C_P}\right)P(t)[M_i(t)+\beta M_{ai}(t)]-d_PP(t) \quad \text{(Equation 6b)}$$

$$\frac{dP(t)}{dt}=k_P\left(1-\frac{P(t)}{C_P}\right)P(t)[M_i(t)+\beta M_{ai}(t)]-k_rP(t)M_{ai}(t)-d_PP(t) \quad \text{(Equation 6c)}$$

Cell-intrinsic and extrinsic properties

A combination of cell-intrinsic and extrinsic properties was then investigated. Similar to the cell-intrinsic cases, the fourth model considers parasite proliferation in both infected iNOS⁻ and iNOS⁺ monocyte-derived phagocytes, with rate k_P, $k_P\left(1-\frac{P(t)}{C_P}\right)P(t)[M_i(t)+M_{ai}(t)]$. In this case however, parasites were not only killed by infected iNOS⁺ but also by non-infected iNOS+ monocytes, with rate k_r, $k_rP(t)[M_{ai}(t)+M_a(t)]$. Additionally, parasites undergo natural death, with rate d_P, $d_PP(t)$ (Equation 6d).

The remaining two models consider parasite proliferation inhibition by the presence of infected and non-infected iNOS⁺ monocyte-derived phagocytes $k_P\left(1-\frac{P(t)}{C_P}\right)P(t)\frac{M_i(t)+M_{ai}(t)}{C_{iP}+M_{ai}(t)+M_a(t)}$. The two models differ in the absence (Equation 6e) or presence (Equation 6f) of active parasite killing, with rate k_r, $k_rP(t)[M_{ai}(t)+M_a(t)]$, by the entire activated compartment.

$$\frac{dP(t)}{dt}=k_P\left(1-\frac{P(t)}{C_P}\right)P(t)[M_i(t)+M_{ai}(t)]-k_rP(t)[M_{ai}(t)+M_a(t)]-d_PP(t) \quad \text{(Equation 6d)}$$

$$\frac{dP(t)}{dt}=k_P\left(1-\frac{P(t)}{C_P}\right)P(t)\frac{M_i(t)+M_{ia}(t)}{C_{iP}+M_{ai}(t)+M_a(t)}-d_PP(t) \quad \text{(Equation 6e)}$$

$$\frac{dP(t)}{dt}=k_P\left(1-\frac{P(t)}{C_P}\right)P(t)\frac{M_i(t)+M_{ai}(t)}{C_{iP}+M_{ai}(t)+M_a(t)}-k_rP(t)[M_{ai}(t)+M_a(t)]-d_PP(t) \quad \text{(Equation 6f)}$$

The initital population of non-infected iNOS⁻, $M(0)$, and *L. major* burden, $P(0)$, were estimated because their amount is not known at the time of infection, whereas the remaining populations were assumed zero, $M_i(0)=M_{ia}(0)=M_a(0)=0$. All the parameter values for the six models can be found on Table S5 (low dose infection) and Table S6 (high dose infection). The initital population of non-infected iNOS⁻, $M(0)$, and *L. major* burden, $P(0)$, were estimated because their amount is not known at the time of infection, whereas the remaining populations were assumed zero, $M_i(0)=M_{ia}(0)=M_a(0)=0$. All the parameter values for the six models can be found on Table S5 (low dose infection) and Table S6 (high dose infection).

The temporal evolution of the number of dying and proliferating parasites was obtained by considering the death (starting with k_r and d_P) and proliferation (starting with k_P) terms from Equations 6a–6f multiplied by P(t) within a time window (t, t+τ), and normalized as percentage of the parasite burden *P(t)* in this time window. The duration in which a pathogen stays in the "dying" state and similarly the duration of a parasite division are unknown, requiring the use of two additional parameters, τ(dying) and τ(proliferating) respectively, in order to be able to recapitulate the low dose death and proliferation data. These two additional parameters together with the

death and proliferation data were taken into account during the parameter estimation of the low dose infection. Numerical simulations were performed by C++, using *MoonFit,* a minimal graphical user interface for fitting ODE dynamical models developed in Qt creator (Robert et al., 2018). The time evolution of the five populations were simulated by a 4th order Runge-Kutta with adaptive time step. Parameter estimation was performed using Stochastic Ranking Evolutionary Strategies (Runarsson and Yao, 2002). In order to constrain the parameter boundaries, we first used an iterative fitting strategy, where each differential equation was analyzed separately, while the dynamics of the remaining differential equations were considered as fixed input by linear interpolation of the experimental data (Binder et al., 2015). Once these initial parameters were identified separately, we defined boundaries around them to further perform a global optimization, where the parameters were estimated by minimizing the residual sum of squares, normalized by the maximum experimental values for each individual variable (i.e., population), $RSS = \frac{\sum_i (y_i - f(x_i))^2}{\max(y_i)}$, where y_i is the experimental mean value for each data point, and $f(x_i)$ is the ODE solution. For model selection the corrected Akaike information criterion (AICc) was calculated by the following formula:

$AICc = 2P + RSS^2 + N\log(2\pi) + 2\frac{P(P+1)}{N-P-1}$, where P is the number of fitted parameters and N the number of data points (Zhao et al., 2017).

Bootstrapping analysis of the fitted model parameters

To identify correlations between different parameters and analyze their constraints, the favorable models (1 and 4) were fitted repeatedly with artificial experimental data which had been derived from the real experimental data by sampling all data points from a normal distribution with mean and standard deviation from the original data. Every optimization was performed with 250 different experimental sets using MATLAB. The distribution of each parameter including the best fit is shown in Figure S2A. In addition, the correlations between the parameter vectors generated from bootstrapping were investigated using Spearman's rank correlation (Figure S2B). No strong correlations (Spearman r > 0.5) were observed between parameters in any of the models, except for k_{Mrec} (basal monocyte recruitment) with d_{Ma} (natural death rate of activated monocytes), α (factor in the denominator for parasite-dependent monocyte recruitment) with d_{Ma} (natural death rate of activated monocytes), and P_0 (initial parasite number) and D_0 (initial delay between infection and Th1 induction). The correlation of k_{Mrec} with d_{Ma} and, to a lesser extent (r = 0.289 in the respective model) with k_a (monocyte activation rate) can be explained by the fact that they can compensate for each other (i.e., a higher basal recruitment can be compensated by higher activation and death of activated cells). The same is true for the correlation between α with d_{Ma}. Due to the uncertainty in the fraction of parasites from the initial inoculum that participate in the establishment of the infection (van Zandbergen et al., 2006) P_0 is difficult to determine and it is not unexpected that higher initial parasite numbers need to be compensated by an increased initial delay function in order to fit the course of immune activation.

QUANTIFICATION AND STATISTICAL ANALYSIS

IV-2PM image processing and quantification of parasite death

IV-2PM stack images spanning the entire site of primary infection were acquired at different time points through the course of disease. CFP fluorescence signal was detected at 465-485 nm (CFP490 channel) and 500-520 nm (CFP520 channel) while mNectarine fluorescence was collected at 565-610 nm (mNectarine channel)(Figure S3C). Using the Imaris Software (Versions 9.3.1. through 9.5.1., Bitplane, Perkin Elmer), a pre-detection channel defined as the geometric mean of the CFP490 and CFP520 channels was calculated (Figure S3D, 1st panel) and masked using a low threshold value to eliminate bleed-through contamination from the second harmonic (SH) signal. The resulting stack was then smoothed with a Gaussian filter and further used as a detection channel. In order to suitably segment both bright viable parasites and their possibly dimmer dying counterparts, a gradient-based segmentation algorithm was applied using both a custom-made Fiji (ImageJ Version 1.51 s, http://imageJ.nij.gov/ij) macro and the Imaris Software. Following contrast enhancement, object outer boundaries were roughly extracted from the detection channel using a Canny-Deriche edge detector (Image Edge plugin written by Thomas Boudier and Joris Meys) and further used as seeds for refined boundary detection using an active contour algorithm (Level Sets plugin, launched by Erwin Frise). Resulting objects were then filtered to retain only those whose CFP490 and CFP520 fluorescence was at least three-fold higher than background values calculated from five different manually-defined ROIs encompassing non-infected areas of the tissue. Inner object boundaries (i.e., gaps within parasite aggregates) were next recreated with the active contour algorithm using as seeds the discrepancies between filtered objects and the low-thresholded detection channel. An object channel, i.e., a binary image stack displaying the objects obtained by the successive boundary detections, was then created. To allow for a better separation of touching objects, a final segmentation channel defined as the geometric mean of the object channel and the detection channel was calculated (Figure S3D, second panel) and used as source for 3D surface recognition in Imaris using an automatically thresholded, local-contrast based, touching object-splitting algorithm. The positions and fluorescence properties of the segmented objects were next exported and converted to cytometry files thanks to the DiscIt software (Figure S3D, third panel). Using the FlowJo software (Version 10.6.2, LLC, BD Biosciences), objects containing saturated pixels were gated out when necessary. Autofluorescent particles exhibiting a high CFP520-to-CFP490 ratio were then excluded and remaining contaminants, characterized by a high mNectarine signal in comparison to their CFP490 content, were eventually eliminated (Figure S3D, fourth panel (histograms). Successive segmentation and filtering procedures enabled the efficient separation of parasites from hair and autofluorescent cells (shown in gray in Figure S3D (fourth and fifth panel), and in red and highlighted by stars and arrows in Figure S3E, upper panel). Coordinates of the selected objects could then be re-injected in Imaris to mask out excluded

particles and generate filtered images (Figure S3E, lower panel). CFP-to-Nectarine ratios of the selected objects, defined as 100*CFP490/mNectarine, were then calculated. The threshold value to identify dying parasites was determined on images from freshly inoculated sites where most parasites are extracellular in a pH neutral environment and thus can be used as a representative baseline for viable parasites (Figures 2B–2D). This threshold was set in a way that all images analyzed at 0h p.i. contained less than 5% dying parasites. The percentages of dying parasites were determined (Figure S3F, lower panel) using this value. The 100*CFP490/mNectarine values, along with object coordinates, were used to draw heatmaps (Figure S3F, upper panel) where live parasites appear purple and dying parasites are highlighted with yellow and white shades.

Image processing and quantification for proliferation measurement

Large field IV-2PM images acquired before, 0h after and 48h after photoconversion were rough segmented according to a summed up channel of mKikume red and green using the Imaris Software (Versions 9.3.1. through 9.5.1., Bitplane, Perkin Elmer). The positions and fluorescence properties of the segmented cells (including autofluorescent objects) were exported and converted to cytometry files using the DiscIt software (Moreau et al., 2012). Autofluorescent objects, such as hair follicles and keratinocytes, were filtered out by excluding objects with a low mKikume green 520/540 nm ratio, as described previously (Müller et al., 2013). Furthermore, objects exhibiting high fluorescence above 625 nm as compared to mKikume red emission (560-620nm) were also excluded from the analysis. The resulting filtered datasets (Figure S4A) were used for data normalization in order to ensure comparability between individual mice. In order to normalize the mKikume red and green values from different mice, the following strategy aligning the fluorescence values before and 0h after photoconversion was established: The median green and red fluorescence of the green values below the 10th and over the 90th percentile of the preconversion controls, and analogously of the red values of the 0h after photoconversion controls were determined and the linear regression between the respective median coordinates was determined (Figure S4B). All fluorescence values (including 48h after photoconversion measurements) were then drift transformed in a way the linear regression curves ran through the 0/0 coordinate (Figure S4C). Cartesian coordinates were then turned into polar coordinates and angular coordinates were normalized with the following formula so that the slope angle of the linear regression of the control before photoconversion becomes 0, and the slope angle of the 0h after photoconversion control equals pi/2 (90°): $\alpha' = \frac{\pi}{2} * \frac{\alpha - \theta}{\theta' - \theta}$ where α' is the normalized angular coordinate, α is the initial angular coordinate, θ is the initial slope angle of the linear regression calculated for the before photoconversion dataset and θ' is the initial slope angle of the linear regression calculated for the 0h after photoconversion dataset. Finally, the datasets were normalized between 0 and the 90th percentile of the control values (Figure S4D).

Statistical analysis

Statistical analysis was performed using the Prism software (Version 8.0, GraphPad). To compare multiple samples within time courses or pairwise analysis within datasets with more than two experimental groups, one-way analysis of variance (ANOVA) were done for datasets that had passed a Shapiro-Wilk normal distribution test, Kruskal-Wallis tests were performed for datasets with non-normal distribution. Appropriate multiple comparison post-tests (Dunnet's for multiple comparisons with the control group in ANOVA, Dunn's test for Kruskal-Wallis analyses) were employed as indicated in the respective figure legends. Two-group comparisons were made by two-sided, unpaired Mann-Whitney tests. Representation of the mean, median and error (in cases in which not all samples are shown individually) are indicated together with sample size in the figure legends.

Immunity, Volume *54*

Supplemental information

Nitric oxide controls proliferation of *Leishmania major* by inhibiting the recruitment of permissive host cells

Pauline Formaglio, Mohamad Alabdullah, Anastasios Siokis, Juliane Handschuh, Ina Sauerland, Yan Fu, Anna Krone, Patricia Gintschel, Juliane Stettin, Sandrina Heyde, Juliane Mohr, Lars Philipsen, Anja Schröder, Philippe A. Robert, Gang Zhao, Sahamoddin Khailaie, Anne Dudeck, Jessica Bertrand, Gerald F. Späth, Sascha Kahlfuß, Philippe Bousso, Burkhart Schraven, Jochen Huehn, Sebastian Binder, Michael Meyer-Hermann, and Andreas J. Müller

Supplemental Materials

Supplemental table 1. Spearman correlations for high dose infection models. Related to figure 1.

Supplemental table 2. Spearman correlations for low dose infection models. Related to figure 1.

Supplemental table 3. Model selection for high dose infection models. Related to figure 3.

Supplemental table 4. Model selection for low dose infection models. Related to figure 3.

Supplemental table 5. Estimated parameters for the high dose infection models. Related to figure 3.

Supplemental table 6. Estimated parameters for the low dose infection models. Related to figure 3.

Supplemental figure S1. ODE models for *L. major* infection. Related to figure 1.

Supplemental figure S2. *In vitro* characterization of cell death reporter parasite *L. major*^{necR}. Related to figure 2.

Supplemental figure S3. Analysis of parasite death in the ongoing infection. Related to figure 3.

Supplemental figure S4. Analysis of parasite proliferation in the ongoing infection. Related to figure 4.

Supplemental figure S5. Inhibition of cell recruitment to the site of infection. Related to figure 5.

Supplemental figure S6. Pathogen burden development under L-NIL with and without blocking of immune cell recruitment. Related to figure 6.

Supplemental figure S7. Ectopic phagocyte entry in late *L. major* infection. Related to figure 7.

Supplemental table 1. Spearman correlations for high dose infection models. Related to figure 1.

	k_{Mrec}	α	S_M	k_i	k_a	k_{ai}	k_{ia}	k_P	k_r	k_D	d_M	d_{Mi}	d_{Mai}	d_{Ma}	d_P	c_P	c_{im}	$M(0)$	$P(0)$	$D(0)$	Model
k_{Mrec}	1.000	-0.132	-0.003	-0.319	0.288	-0.053	0.007	-0.151	-0.239	0.122	-0.014	-0.076	0.135	0.636	0.095	0.069	0.086	-0.105	-0.002	0.172	1
α	-0.132	1.000	0.023	0.032	0.126	0.050	-0.106	-0.163	-0.015	-0.103	-0.060	-0.024	0.092	0.133	0.119	-0.021	-0.081	-0.033	-0.028	0.068	1
S_M	-0.003	0.023	1.000	0.052	-0.082	0.040	-0.010	-0.037	0.071	0.100	-0.121	-0.073	0.071	-0.099	-0.073	0.120	0.123	0.089	0.025	0.004	1
k_i	-0.319	0.032	0.052	1.000	-0.100	0.135	0.014	-0.164	0.051	0.133	0.060	-0.075	-0.085	-0.162	0.064	-0.022	-0.030	0.015	-0.198	0.169	1
k_a	0.288	0.126	-0.082	-0.100	1.000	-0.053	0.051	-0.227	0.000	-0.012	0.028	-0.012	0.073	-0.011	0.029	-0.005	0.098	-0.040	-0.206	0.012	1
k_{ai}	-0.053	0.050	0.040	0.135	-0.053	1.000	0.011	0.305	0.057	-0.057	0.006	0.002	0.012	-0.102	0.045	0.091	-0.016	0.010	0.175	-0.096	1
k_{ia}	0.007	-0.106	-0.010	0.014	0.051	0.011	1.000	0.101	-0.003	-0.022	-0.010	0.117	-0.154	-0.086	0.141	0.017	0.063	0.006	-0.013	-0.024	1
k_P	-0.151	-0.163	-0.037	-0.164	-0.227	0.305	0.101	1.000	0.429	0.036	-0.040	0.048	-0.322	-0.051	0.024	-0.023	-0.041	0.087	-0.195	0.154	1
k_r	-0.239	-0.015	0.071	0.051	0.000	0.057	-0.003	0.429	1.000	-0.172	-0.082	-0.075	0.139	-0.212	0.033	-0.050	-0.054	0.123	-0.258	-0.111	1
k_D	0.122	-0.103	0.100	0.133	-0.012	-0.057	-0.022	0.036	-0.172	1.000	-0.039	-0.091	-0.086	0.057	0.061	0.131	0.055	-0.133	-0.370	-0.137	1
d_M	-0.014	-0.060	-0.121	0.060	0.028	0.006	-0.010	-0.040	-0.082	-0.039	1.000	0.059	-0.033	0.060	-0.059	-0.072	-0.008	-0.002	-0.053	-0.055	1
d_{Mi}	-0.076	-0.024	-0.073	-0.075	-0.012	0.002	0.117	0.048	-0.075	-0.091	0.059	1.000	-0.027	-0.041	0.052	-0.020	0.027	0.052	0.083	-0.018	1
d_{Mai}	0.135	0.092	0.071	-0.085	0.073	0.012	-0.154	-0.322	0.139	-0.086	-0.033	-0.027	1.000	0.192	0.005	0.054	-0.020	-0.050	-0.048	-0.072	1
d_{Ma}	0.636	0.133	-0.099	-0.162	-0.011	-0.102	-0.086	-0.051	-0.212	0.057	0.060	-0.041	0.192	1.000	0.068	-0.013	-0.087	-0.052	0.087	0.068	1
d_P	0.095	0.119	-0.073	0.064	0.029	0.045	0.141	0.024	0.033	0.061	-0.059	0.052	0.005	0.068	1.000	-0.019	0.168	0.093	-0.009	0.038	1
c_P	0.069	-0.021	0.120	-0.022	-0.005	0.091	0.017	-0.023	-0.050	0.131	-0.072	-0.020	0.054	-0.013	-0.019	1.000	-0.037	-0.001	0.141	-0.047	1
c_{im}	0.086	-0.081	0.123	-0.030	0.098	-0.016	0.063	-0.041	-0.054	0.055	-0.008	0.027	-0.020	-0.087	0.168	-0.037	1.000	0.098	-0.090	-0.135	1
$M(0)$	-0.105	-0.033	0.089	0.015	-0.040	0.010	0.006	0.087	0.123	-0.133	-0.002	0.052	-0.050	-0.052	0.093	-0.001	0.098	1.000	0.036	-0.016	1
$P(0)$	-0.002	-0.028	0.025	-0.198	-0.206	0.175	-0.013	-0.195	-0.258	-0.370	-0.053	0.083	-0.048	0.087	-0.009	0.141	-0.090	0.036	1.000	0.113	1
$D(0)$	0.172	0.068	0.004	0.169	0.012	-0.096	-0.024	0.154	-0.111	-0.137	-0.055	-0.018	-0.072	0.068	0.038	-0.047	-0.135	-0.016	0.113	1.000	1
k_{Mrec}	1.000	0.020	0.019	0.007	0.016	0.041	-0.094	0.038	0.010	-0.029	-0.007	-0.021	-0.010	0.189	-0.028	0.014	-0.016	-0.017	-0.099	-0.075	4
α	0.020	1.000	0.274	0.109	0.311	0.168	0.162	-0.005	0.058	0.242	0.000	0.167	0.418	0.132	-0.088	0.252	-0.119	-0.108	-0.323	-0.129	4
S_M	0.019	0.274	1.000	0.112	0.008	-0.057	0.023	-0.023	-0.034	0.048	0.027	-0.005	0.117	0.012	-0.131	-0.110	0.046	-0.075	0.194	0.003	4
k_i	0.007	0.109	0.112	1.000	-0.079	0.047	0.076	-0.253	0.076	0.266	0.076	0.044	0.026	-0.103	0.034	-0.143	-0.014	0.119	-0.201	0.134	4
k_a	0.016	0.311	0.008	-0.079	1.000	-0.099	-0.048	0.077	-0.370	0.107	-0.091	0.143	0.052	-0.123	-0.025	0.056	0.031	-0.075	0.127	0.115	4
k_{ai}	0.041	0.168	-0.057	0.047	-0.099	1.000	0.004	-0.129	-0.104	-0.038	0.092	0.093	-0.013	0.125	-0.020	0.302	0.269	-0.007	-0.442	-0.279	4
k_{ia}	-0.094	0.162	0.023	0.076	-0.048	0.004	1.000	-0.170	0.113	0.006	-0.112	0.107	0.030	0.022	0.069	-0.127	-0.026	0.098	-0.056	0.017	4
k_P	0.038	-0.005	-0.023	-0.253	0.077	-0.129	-0.170	1.000	0.409	0.087	-0.013	-0.073	-0.047	0.013	0.022	-0.337	-0.043	-0.078	-0.053	0.161	4
k_r	0.010	0.058	-0.034	0.076	-0.370	-0.104	0.113	0.409	1.000	-0.024	0.081	-0.032	0.023	-0.020	0.019	0.112	0.075	-0.010	-0.326	-0.116	4
k_D	-0.029	0.242	0.048	0.266	0.107	-0.038	0.006	0.087	-0.024	1.000	0.131	0.087	0.004	-0.113	0.005	-0.081	0.099	0.109	-0.165	-0.175	4
d_M	-0.007	0.000	0.027	0.076	-0.091	0.092	-0.112	-0.013	0.081	0.131	1.000	-0.053	0.066	0.018	-0.028	0.031	0.093	-0.085	-0.157	-0.196	4
d_{Mi}	-0.021	0.167	-0.005	0.044	0.143	0.093	0.107	-0.073	-0.032	0.087	-0.053	1.000	0.076	-0.011	0.040	0.031	0.113	-0.021	-0.177	-0.078	4
d_{Mai}	-0.010	0.418	0.117	0.026	0.052	-0.013	0.030	-0.047	0.023	0.004	0.066	0.076	1.000	0.087	-0.118	0.147	-0.035	-0.141	-0.190	-0.088	4
d_{Ma}	0.189	0.132	0.012	-0.103	-0.123	0.125	0.022	0.013	-0.020	-0.113	0.018	-0.011	0.087	1.000	-0.027	-0.037	0.042	-0.072	-0.043	-0.005	4
d_P	-0.028	-0.088	-0.131	0.034	-0.025	-0.020	0.069	0.022	0.019	0.005	-0.028	0.040	-0.118	-0.027	1.000	-0.034	0.017	-0.028	0.067	0.069	4
c_P	0.014	0.252	-0.110	-0.143	0.056	0.302	-0.127	-0.337	0.112	-0.081	0.031	0.031	0.147	-0.037	-0.034	1.000	-0.100	-0.015	-0.157	-0.159	4
c_{im}	-0.016	-0.119	0.046	-0.014	0.031	0.269	-0.026	-0.043	0.075	0.099	0.093	0.113	-0.035	0.042	0.017	-0.100	1.000	0.007	0.063	-0.055	4
$M(0)$	-0.017	-0.108	-0.075	0.119	-0.075	-0.007	0.098	-0.078	-0.010	0.109	-0.085	-0.021	-0.141	-0.072	-0.028	-0.015	0.007	1.000	-0.028	0.004	4
$P(0)$	-0.099	-0.323	0.194	-0.201	0.127	-0.442	-0.056	-0.053	-0.326	-0.165	-0.157	-0.177	-0.190	-0.043	0.067	-0.157	0.063	-0.028	1.000	0.609	4
$D(0)$	-0.075	-0.129	0.003	0.134	0.115	-0.279	0.017	0.161	-0.116	-0.175	-0.196	-0.078	-0.088	-0.005	0.069	-0.159	-0.055	0.004	0.609	1.000	4

Correlations between the parameter vectors generated from bootstrapping in high dose models 1 and 4 analyzed by Spearman's rank correlation. Color coding range: -0.372 (white) to 1 (blue) for model 1, -0.448 (white) to 1 (blue) for model 4.

Supplemental table 2. Spearman correlations for low dose infection models. Related to figure 1.

	k_{Mrec}	α	S_M	k_i	k_a	k_{ai}	k_{ia}	k_P	k_r	k_D	d_M	d_{Mi}	d_{Mai}	d_{Ma}	d_P	c_P	c_{im}	$M(0)$	$P(0)$	$D(0)$	Model
k_{Mrec}	1.000	-0.069	-0.107	0.006	-0.031	0.125	-0.040	-0.011	-0.032	-0.065	0.024	-0.005	0.051	-0.020	-0.013	-0.062	-0.008	0.099	0.013	0.040	1
α	-0.069	1.000	0.340	0.056	0.173	-0.040	-0.003	-0.079	-0.064	-0.042	0.050	-0.144	0.019	0.496	-0.134	-0.119	-0.147	0.079	-0.365	-0.007	1
S_M	-0.107	0.340	1.000	-0.023	0.026	0.057	-0.010	0.054	0.054	-0.023	0.047	-0.143	0.005	0.087	-0.115	0.008	-0.008	0.126	0.057	0.056	1
k_i	0.006	0.056	-0.023	1.000	0.051	-0.077	-0.021	-0.324	-0.224	0.076	0.053	0.049	0.177	0.084	-0.010	-0.313	0.071	-0.036	-0.395	0.110	1
k_a	-0.031	0.173	0.026	0.051	1.000	0.011	0.002	-0.201	0.015	-0.163	0.003	-0.079	0.010	0.215	-0.157	-0.147	0.282	-0.036	-0.151	0.113	1
k_{ai}	0.125	-0.040	0.057	-0.077	0.011	1.000	-0.088	0.206	-0.305	0.050	-0.021	0.025	-0.043	-0.156	0.117	0.060	0.037	-0.041	-0.031	0.014	1
k_{ia}	-0.040	-0.003	-0.010	-0.021	0.002	-0.088	1.000	0.112	-0.174	0.147	0.055	0.008	-0.035	0.001	-0.070	-0.002	0.069	0.004	0.031	0.085	1
k_P	-0.011	-0.079	0.054	-0.324	-0.201	0.206	0.112	1.000	0.207	0.056	-0.087	-0.011	-0.297	-0.114	0.134	-0.041	-0.243	-0.156	-0.278	0.142	1
k_r	-0.032	-0.064	0.054	-0.224	0.015	-0.305	-0.174	0.207	1.000	-0.150	-0.151	-0.091	0.277	-0.074	-0.011	0.096	0.176	-0.039	-0.056	-0.059	1
k_D	-0.065	-0.042	-0.023	0.076	-0.163	0.050	0.147	0.056	-0.150	1.000	-0.074	0.019	-0.073	0.086	-0.026	-0.303	0.152	0.055	-0.234	-0.128	1
d_M	0.024	0.050	0.047	0.053	0.003	-0.021	0.055	-0.087	-0.151	-0.074	1.000	0.017	0.020	0.022	-0.034	-0.009	-0.089	-0.023	0.067	-0.031	1
d_{Mi}	-0.005	-0.144	-0.143	0.049	-0.079	0.025	0.008	-0.011	-0.091	0.019	0.017	1.000	0.079	-0.018	0.010	0.050	0.010	-0.024	0.058	-0.032	1
d_{Mai}	0.051	0.019	0.005	0.177	0.010	-0.043	-0.035	-0.297	0.277	-0.073	0.020	0.079	1.000	0.105	-0.014	-0.123	-0.054	-0.033	0.082	-0.111	1
d_{Ma}	-0.020	0.496	0.087	0.084	0.215	-0.156	0.001	-0.114	-0.074	0.086	0.022	-0.018	0.105	1.000	-0.158	-0.213	-0.047	-0.020	-0.138	0.044	1
d_P	-0.013	-0.134	-0.115	-0.010	-0.157	0.117	-0.070	0.134	-0.011	-0.026	-0.034	0.010	-0.014	-0.158	1.000	0.103	-0.078	-0.075	0.009	-0.007	1
c_P	-0.062	-0.119	0.008	-0.313	-0.147	0.060	-0.002	-0.041	0.096	-0.303	-0.009	0.050	-0.123	-0.213	0.103	1.000	0.008	0.013	0.226	-0.101	1
c_{im}	-0.008	-0.147	-0.008	0.071	0.282	0.037	0.069	-0.243	0.176	0.152	-0.089	0.010	-0.054	-0.047	-0.078	0.008	1.000	0.114	0.219	-0.023	1
$M(0)$	0.099	0.079	0.126	-0.036	-0.036	-0.041	0.004	-0.156	-0.039	0.055	-0.023	-0.024	-0.033	-0.020	-0.075	0.013	0.114	1.000	0.096	0.001	1
$P(0)$	0.013	-0.365	0.057	-0.395	-0.151	-0.031	0.031	-0.278	-0.056	-0.234	0.067	0.058	0.082	-0.138	0.009	0.226	0.219	0.096	1.000	-0.123	1
$D(0)$	0.040	-0.007	0.056	0.110	0.113	0.014	0.085	0.142	-0.059	-0.128	-0.031	-0.032	-0.111	0.044	-0.007	-0.101	-0.023	0.001	-0.123	1.000	1
k_{Mrec}	1.000	-0.286	0.117	-0.062	0.047	-0.090	0.026	-0.070	0.009	-0.006	-0.079	-0.040	0.004	0.081	0.028	0.011	0.013	-0.011	0.092	0.045	4
α	-0.286	1.000	-0.003	0.021	0.316	0.050	0.053	-0.127	-0.102	0.024	0.172	-0.020	0.054	0.545	0.031	-0.051	-0.111	-0.190	-0.026	0.008	4
S_M	0.117	-0.003	1.000	-0.058	-0.077	-0.049	0.020	-0.002	-0.009	0.151	-0.061	-0.013	-0.053	0.010	0.111	-0.106	-0.076	-0.009	0.070	0.066	4
k_i	-0.062	0.021	-0.058	1.000	0.143	-0.160	0.012	-0.436	0.013	-0.060	0.020	0.082	0.205	-0.023	-0.039	-0.294	0.064	-0.038	-0.268	0.025	4
k_a	0.047	0.316	-0.077	0.143	1.000	-0.104	-0.055	-0.201	-0.248	-0.239	-0.022	0.005	0.143	0.294	0.012	-0.201	0.335	-0.061	-0.178	0.072	4
k_{ai}	-0.090	0.050	-0.049	-0.160	-0.104	1.000	0.092	0.144	-0.276	0.033	0.037	-0.011	-0.237	-0.133	0.115	0.249	0.035	0.053	0.026	-0.033	4
k_{ia}	0.026	0.053	0.020	0.012	-0.055	0.092	1.000	-0.064	0.119	-0.007	-0.005	-0.077	0.007	0.051	0.057	0.132	-0.042	0.024	0.026	0.063	4
k_P	-0.070	-0.127	-0.002	-0.436	-0.201	0.144	-0.064	1.000	0.026	0.049	0.000	0.000	-0.185	-0.055	0.115	-0.082	-0.151	0.048	-0.149	0.138	4
k_r	0.009	-0.102	-0.009	0.013	-0.248	-0.276	0.119	0.026	1.000	0.099	-0.040	-0.025	0.035	0.012	-0.063	0.354	0.203	0.007	-0.175	0.108	4
k_D	-0.006	0.024	0.151	-0.060	-0.239	0.033	-0.007	0.049	0.099	1.000	0.059	-0.035	-0.221	0.157	-0.001	-0.137	0.038	-0.009	-0.246	-0.067	4
d_M	-0.079	0.172	-0.061	0.020	-0.022	0.037	-0.005	0.000	-0.040	0.059	1.000	0.011	-0.022	-0.014	0.067	0.021	-0.006	-0.080	-0.086	-0.085	4
d_{Mi}	-0.040	-0.020	-0.013	0.082	0.005	-0.011	-0.077	0.000	-0.025	-0.035	0.011	1.000	0.055	-0.072	-0.080	-0.053	0.089	-0.007	0.000	-0.011	4
d_{Mai}	0.004	0.054	-0.053	0.205	0.143	-0.237	0.007	-0.185	0.035	-0.221	-0.022	0.055	1.000	0.111	0.019	-0.004	0.101	-0.059	-0.083	-0.054	4
d_{Ma}	0.081	0.545	0.010	-0.023	0.294	-0.133	0.051	-0.055	0.012	0.157	-0.014	-0.072	0.111	1.000	0.065	-0.060	-0.015	-0.040	0.049	0.058	4
d_P	0.028	0.031	0.111	-0.039	0.012	0.115	0.057	0.115	-0.063	-0.001	0.067	-0.080	0.019	0.065	1.000	-0.025	0.000	-0.154	0.056	-0.020	4
c_P	0.011	-0.051	-0.106	-0.294	-0.201	0.249	0.132	-0.082	0.354	-0.137	0.021	-0.053	-0.004	-0.060	-0.025	1.000	-0.090	-0.041	0.122	-0.052	4
c_{im}	0.013	-0.111	-0.076	0.064	0.335	0.035	-0.042	-0.151	0.203	0.038	-0.006	0.089	0.101	-0.015	0.000	-0.090	1.000	0.090	-0.060	-0.011	4
$M(0)$	-0.011	-0.190	-0.009	-0.038	-0.061	0.053	0.024	0.048	0.007	-0.009	-0.080	-0.007	-0.059	-0.040	-0.154	-0.041	0.090	1.000	-0.112	0.023	4
$P(0)$	0.092	-0.026	0.070	-0.268	-0.178	0.026	0.026	-0.149	-0.175	-0.246	-0.086	0.000	-0.083	0.049	0.056	0.122	-0.060	-0.112	1.000	-0.054	4
$D(0)$	0.045	0.008	0.066	0.025	0.072	-0.033	0.063	0.138	0.108	-0.067	-0.085	-0.011	-0.054	0.058	-0.020	-0.052	-0.011	0.023	-0.054	1.000	4

Correlations between the parameter vectors generated from bootstrapping in low dose models 1 and 4 analyzed by Spearman's rank correlation. Color coding range: -0.395 (white) to 1 (blue) for model 1, -0.435 (white) to 1 (blue) for model 4.

Supplemental table 3. Model selection for high dose infection models. Related to figure 3.

	P (# params.)	RSS	AICc	Δ(AICc) = M4(best)-MX	$e^{2*\Delta(AICc)}$	Status
M1	20	1.38284	159.18	1.66	27.5279	Cannot be rejected
M2	20	2.76479	164.92	7.39	2.62E+06	Rejected due to $e^{2*\Delta(AICc)}$
M3	21	0.970483	182.38	24.85	3.87E+21	Rejected due to $e^{2*\Delta(AICc)}$
M4	20	0.504625	157.53	0.00	1.0000	Best
M5	20	2.06314	161.53	4.00	2.99E+03	Rejected due to $e^{2*\Delta(AICc)}$
M6	21	1.73764	184.46	26.93	2.47E+23	Rejected due to $e^{2*\Delta(AICc)}$

Supplemental table 4. Model selection for low dose infection models. Related to figure 3.

	P (# params.)	RSS	AICc	Δ(AICc) = M4(best)-M_X	$e^{2*\Delta(\text{AICc})}$	Status
M1	24	0.774539	209.0638719	0.108564339	1.242503975	Cannot be rejected
M2	24	1.46628	210.6139383	1.658630715	27.58470437	Rejected due to bad fit of death reporter
M3	25	0.638547	240.0383702	31.08306261	9.96334E+26	Rejected due to $e^{2*\Delta(\text{AICc})}$
M4	24	0.700961	208.9553075	0	1	Best
M5	24	1.24545	210.0151069	1.059799379	8.327795356	Rejected due to bad fit of death reporter
M6	25	0.893855	240.4296046	31.4742971	2.17885E+27	Rejected due to $e^{2*\Delta(\text{AICc})}$

Supplemental table 5. Estimated parameters for the high dose infection models. Related to figure 3.

	M1	M2	M3	M4	M5	M6	Lower bound	Upper bound	
k_{Mrec}	6720.98	22.3587	131.481	150.353	1586.96	1.51174	0.75127	13778.4	Estimated
α	3325.33	10272.2	8490.96	10766.3	4892.54	8204.17	1484.1	16500.8	Estimated
S_M	2861.09	13750.2	24467.8	36132.5	12592.9	38974.8	1341.78	69952.9	Estimated
k_i	2.32E-07	4.23E-08	8.80E-08	6.89E-08	4.67E-08	6.15E-08	1.55E-08	3.60E-07	Estimated
k_a	2562.36	4623.33	2441.12	5369.19	2260.13	3408.12	989.025	6346.5	Estimated
k_{ai}	15658.9	9606.75	9473.94	7109.04	5509.81	7178.53	2807.55	32612	Estimated
k_{ia}	4.82E-07	2.42E-06	4.28E-07	1.48E-06	4.49E-07	8.40E-07	1.49E-07	4.34E-06	Estimated
k_P	1.89E-04	7.19E-07	1.57E-04	1.00E-04	4.23E-02	2.93E-03	4.35E-07	6.59E-02	Estimated
k_r	5.04E-04	-	3.89E-04	7.88E-05	-	1.30E-05	4.69E-06	7.87E-04	Estimated
k_D	1.54E-06	1.32E-06	2.44E-07	1.66E-06	9.95E-07	9.62E-07	1.64E-07	2.70E-06	Estimated
d_M	1.26E-07	7.80E-05	2.39E-07	7.28E-04	1.36E-06	1.02E-05	8.69E-08	9.37E-04	Estimated
d_{Mi}	3.11E-07	3.82E-07	6.66E-08	3.07E-07	2.08E-07	1.87E-06	3.83E-08	3.88E-06	Estimated
d_{Mai}	1.13637	0.243529	1.28927	3.09039	9.56808	8.76902	0.137962	23.9283	Estimated
d_{Ma}	1.02593	0.537217	0.534353	0.203856	0.395066	0.238159	0.084326	2.24136	Estimated
d_P	9.27E-03	3.04E-02	1.64E-02	8.86E-04	1.24E-01	0.0016496	7.06E-04	0.128902	Estimated
c_P	3.25E+06	1.09E+07	2.11E+06	1.88E+06	1.12E+06	1.62E+06	406256	11138500	Estimated
c_{im}	6.43374	5133.66	3445.86	8470.43	81.2739	8865.47	3.17876	20645.7	Estimated
β	-	5.55E-05	0.148554	-	-	-	1.00E-07	1.5	Estimated
c_{iP}	-	-	-	-	0.0147527	0.0207067	7.55E-03	59272.1	Estimated
$M(0)$	3224.42	3.35713	5.82213	17.0632	170.318	8690.27	1	11714.7	Estimated
$M_i(0)$	0.0	0.0	0.0	0.0	0.0	0.0	0	0	Estimated
$M_{ai}(0)$	0.0	0.0	0.0	0.0	0.0	0.0	0	0	Estimated
$M_a(0)$	0.0	0.0	0.0	0.0	0.0	0.0	0	0	Estimated
$P(0)$	9822.03	73737.5	30814.1	54215.1	22869.1	12282.2	1000	100000	Estimated
$D(0)$	0.0932255	0.108552	0.0471096	7.07E-03	2.60E-05	1.62E-04	1.00E-05	1.1	Estimated

Supplemental table 6. Estimated parameters for the low dose infection models. Related to figure 3.

	M1	M2	M3	M4	M5	M6	Lower bound	Upper bound	
k_{Mrec}	1.35924	1.75111	6.15908	938.91	356.249	172.026	0.559785	1072.71	Estimated
α	3157.12	3016.22	3423.29	2484.91	3975.56	4337.72	1097.9	6787.62	Estimated
S_{M}	12108.7	8756.37	11180.4	3991.47	13670.1	10446.7	1878.32	40498.9	Estimated
k_{i}	5.60E-08	9.41E-08	8.97E-08	7.45E-08	6.62E-08	5.40E-08	1.98E-08	1.12E-07	Estimated
k_{a}	1252.25	1351.19	1735.94	317.774	1603.01	1568.01	142.701	4434.23	Estimated
k_{ai}	8772.05	14014.8	18304.6	1647.12	12820.3	9002.05	1342.38	22539.3	Estimated
k_{ia}	8.40E-07	4.34E-07	1.24E-06	1.15E-07	2.90E-06	4.49E-07	4.10E-08	3.02E-06	Estimated
k_{P}	5.25E-05	5.12E-05	7.67E-05	1.18E-04	5.44E-03	9.55E-03	4.47E-05	2.28E-02	Estimated
k_{r}	1.26E-04	-	1.43E-04	2.77E-05	-	6.51E-06	4.01E-06	3.76E-04	Estimated
k_{D}	1.34E-06	2.50E-06	1.02E-06	1.43E-06	3.07E-06	2.36E-06	5.70E-07	4.64E-06	Estimated
d_{M}	7.78E-04	2.49E-03	7.03E-04	3.81E-03	3.74E-03	4.01E-03	4.97E-04	9.52E-03	Estimated
d_{Mi}	4.75E-06	1.18E-05	1.79E-05	6.82E-05	6.96E-06	5.85E-06	2.27E-06	6.87E-05	Estimated
d_{Mai}	1.50006	1.75289	2.27229	1.31671	2.64091	1.40927	0.476245	2.96067	Estimated
d_{Ma}	1.46654	1.35232	1.47093	1.94352	1.33362	1.9146	0.660975	2.78754	Estimated
d_{P}	2.76E-03	2.76E-03	2.80E-03	7.70E-05	3.00E-02	8.08E-03	3.24E-05	3.74E-02	Estimated
c_{P}	183269	93422.8	170755	146714	813192	506376	41526.3	837261	Estimated
c_{im}	15741.3	13581.7	23632.1	1447.27	19100.7	12264.5	1443.48	36022.9	Estimated
β	-	3.60E-02	9.42E-06	-	-	-	3.37E-06	1.5	Estimated
c_{iP}	-	-	-	-	0.013912	1.02309	1.38E-02	59272.1	Estimated
$M(0)$	0.781359	5567.73	3299.72	18493.5	2984.04	1025.89	0.52049	28128.5	Estimated
$M_{\mathrm{i}}(0)$	0.0	0.0	0.0	0.0	0.0	0.0	0	0	Estimated
$M_{\mathrm{ai}}(0)$	0.0	0.0	0.0	0.0	0.0	0.0	0	0	Estimated
$M_{\mathrm{a}}(0)$	0.0	0.0	0.0	0.0	0.0	0.0	0	0	Estimated
$P(0)$	30260.5	18901.7	19445.7	11183.2	3818.89	5069.03	1655.51	38545.2	Estimated
$D(0)$	1.40E-02	3.85E-03	5.02E-02	4.54E-02	1.95E-03	3.73E-03	6.76E-04	5.07E-02	Estimated
τ (dying)	1.06159	16.0485	0.994122	0.98037	1.46791	2.4495	0.001	1000	Estimated
τ (proliferating)	4.51897	5.36006	3.34886	2.61633	1.68258	2.36849	0.001	50	Estimated

Supplemental figure S1. ODE models for *L. major* infection. Related to figure 1.

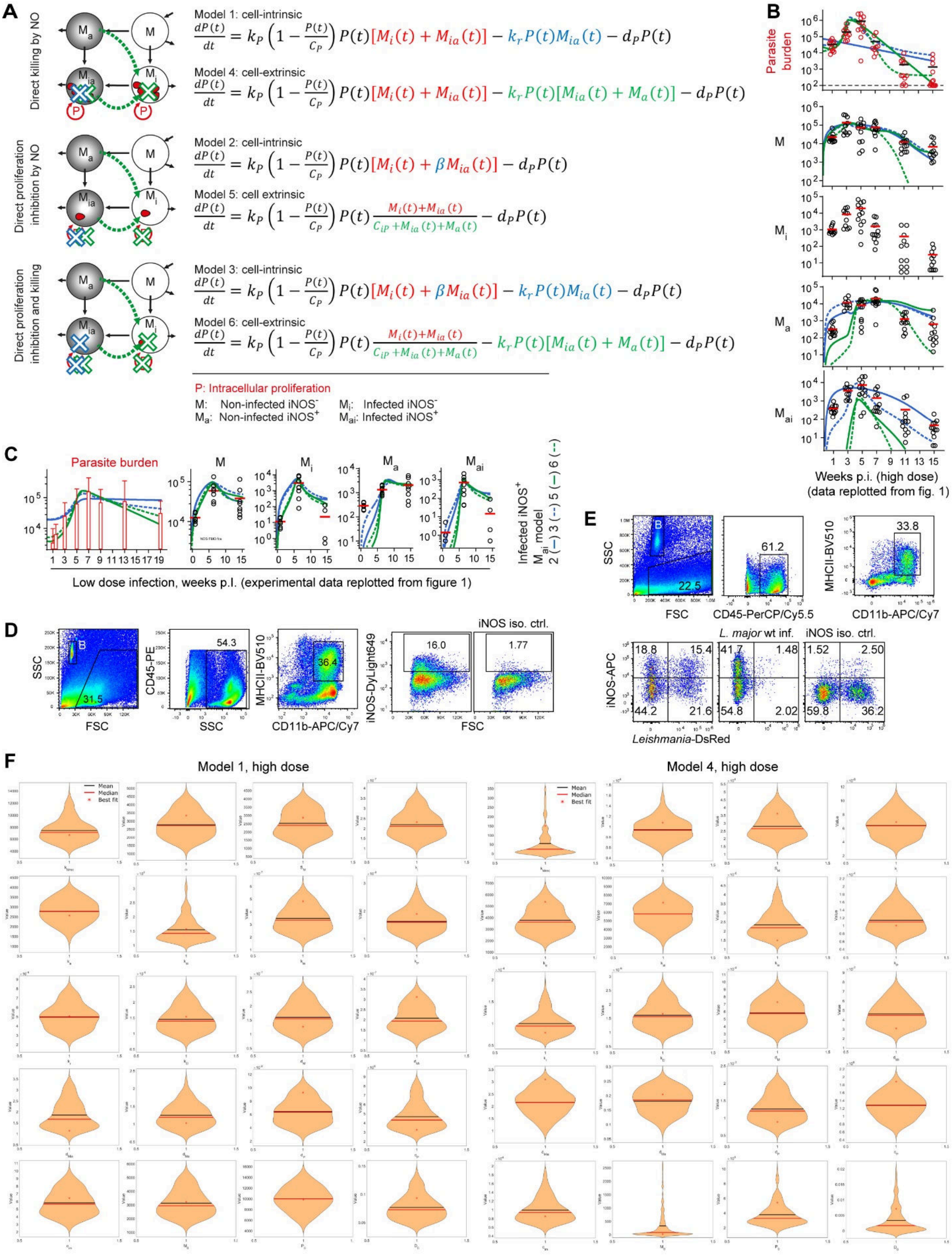

$$\frac{dP(t)}{dt} = k_P\left(1-\frac{P(t)}{C_P}\right)P(t)[M_i(t)+M_{ia}(t)] - k_r P(t) M_{ia}(t) - d_P P(t)$$

$$\frac{dP(t)}{dt} = k_P\left(1-\frac{P(t)}{C_P}\right)P(t)[M_i(t)+M_{ia}(t)] - k_r P(t) [M_{ia}(t)+M_a(t)] - d_P P(t)$$

$$\frac{dP(t)}{dt} = k_P\left(1-\frac{P(t)}{C_P}\right)P(t)[M_i(t)+\beta M_{ia}(t)] - d_P P(t)$$

$$\frac{dP(t)}{dt} = k_P\left(1-\frac{P(t)}{C_P}\right)P(t)\frac{M_i(t)+M_{ia}(t)}{C_{iP}+M_{ia}(t)+M_a(t)} - d_P P(t)$$

$$\frac{dP(t)}{dt} = k_P\left(1-\frac{P(t)}{C_P}\right)P(t)[M_i(t)+\beta M_{ia}(t)] - k_r P(t) M_{ia}(t) - d_P P(t)$$

$$\frac{dP(t)}{dt} = k_P\left(1-\frac{P(t)}{C_P}\right)P(t)\frac{M_i(t)+M_{ia}(t)}{C_{iP}+M_{ia}(t)+M_a(t)} - k_r P(t)[M_{ia}(t)+M_a(t)] - d_P P(t)$$

A. Representation of parasite proliferation dependency of recruited cell number (red), cell intrinsic killing and direct growth inhibition (blue) and cell extrinsic killing and growth inhibition (green) in the differential equations for parasite burden over time in the six different models. **B-C.** Overlays of modeled parasite burden and evolution of the different monocyte-derived cell populations over time in model 3 through 6 for high dose (B) and low dose (C) infections. Experimental data (circles and open bars) are replotted from figure 1. **D-E.** Gating examples for determining induction of the immune response (D) in and determination of the M, Ma, Mi, and Mai populations (E). Examples from 5 weeks post infection (wpi) acquired on a LSRFortessa (D) and Attune NxT flow (E) cytometer are shown. B, Gate for counting beads. **F.** 250 different artificial datasets derived from experimental data by sampling all data points from a normal distribution with mean and standard deviation from the original data were used to repeatedly fit model 1 and 4. The fitted parameters are shown as violin plots, including the mean (black line) and median (red line) of each distribution as well as the parameter value of the best fit (asterisk) of the respective model.

Supplemental figure S2. ***In vitro*** **characterization of cell death reporter parasite** ***L. major*****necR. Related to figure 2.**

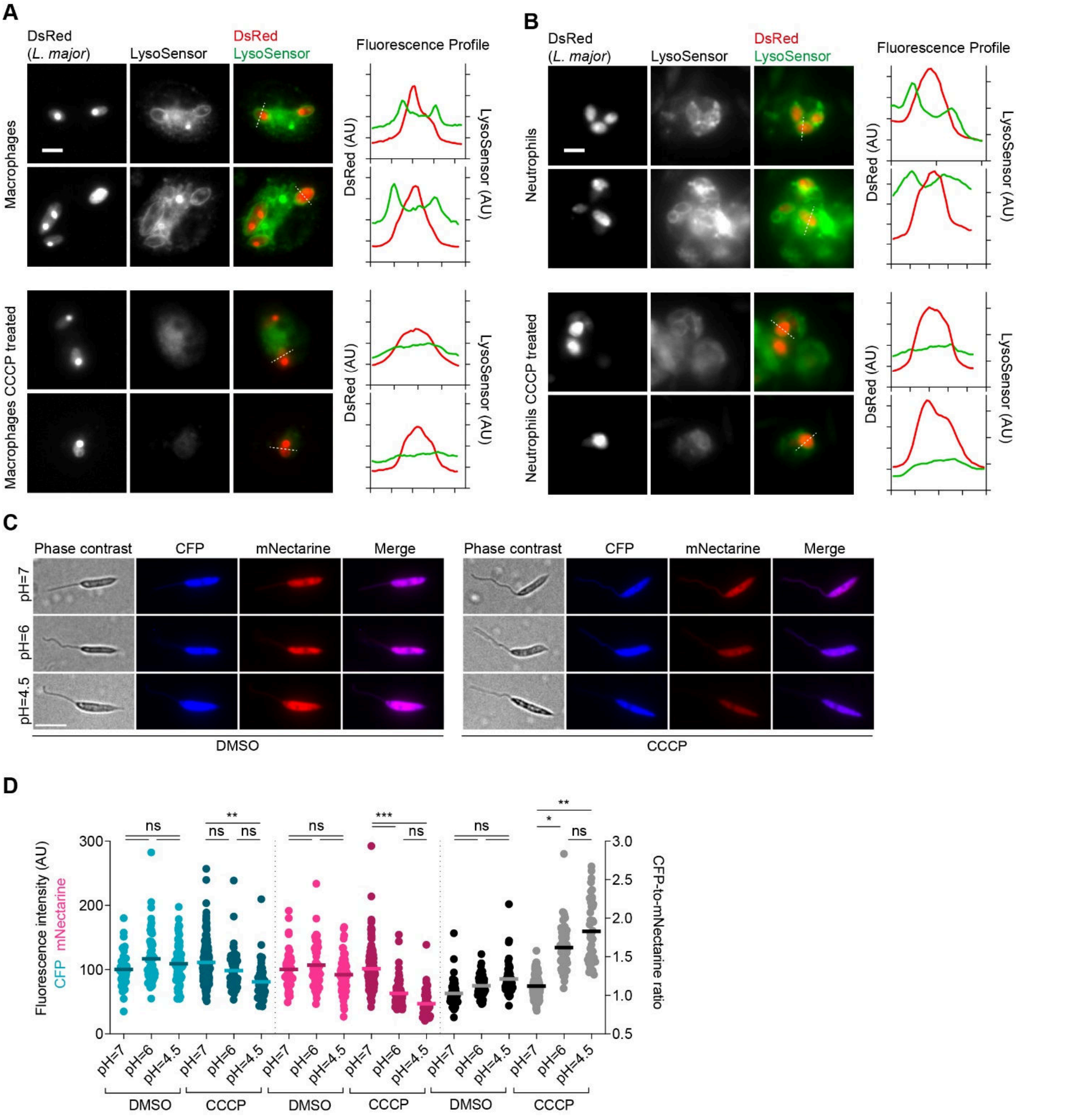

A-B. C57BL/6 peritoneal macrophages (A) and neutrophils purified from mouse bone marrow (B) infected with DsRed-expressing *L. major* (red) stained with LysoSensor Green to highlight acidic compartments within the cells. Upper panels: Two examples of infected phagocytes with a LysoSensor positive compartment surrounding the parasites. Lower panels: Controls treated with the protonophore CCCP which abolishes cellular pH gradients. Fluorescence profiles show parasite (red) and LysoSensor (green) fluorescence intensity along the dotted lines. Scale bar, 5 µm. **C.** Example images of *L. major*^{necR} promastigotes incubated with 0.01% DMSO (control) or 10 µM of the protonophore CCCP in PBS buffered at the indicated pH. Scale bar, 10 µm. **D.** Quantification of CFP and mNectarine fluorescence intensity of individual parasites (left axis) and corresponding CFP-to-Nectarine ratio (right axis) after treatment with CCCP or DMSO at different pH. Horizontal bars denote the mean. ***, $p<0.001$; **, $p>0.01$; *, $p>0.05$; ns, not significant according to one-way ANOVA with Dunn's post-test.

Supplemental figure S3. Analysis of parasite death the ongoing infection. Related to figure 3.

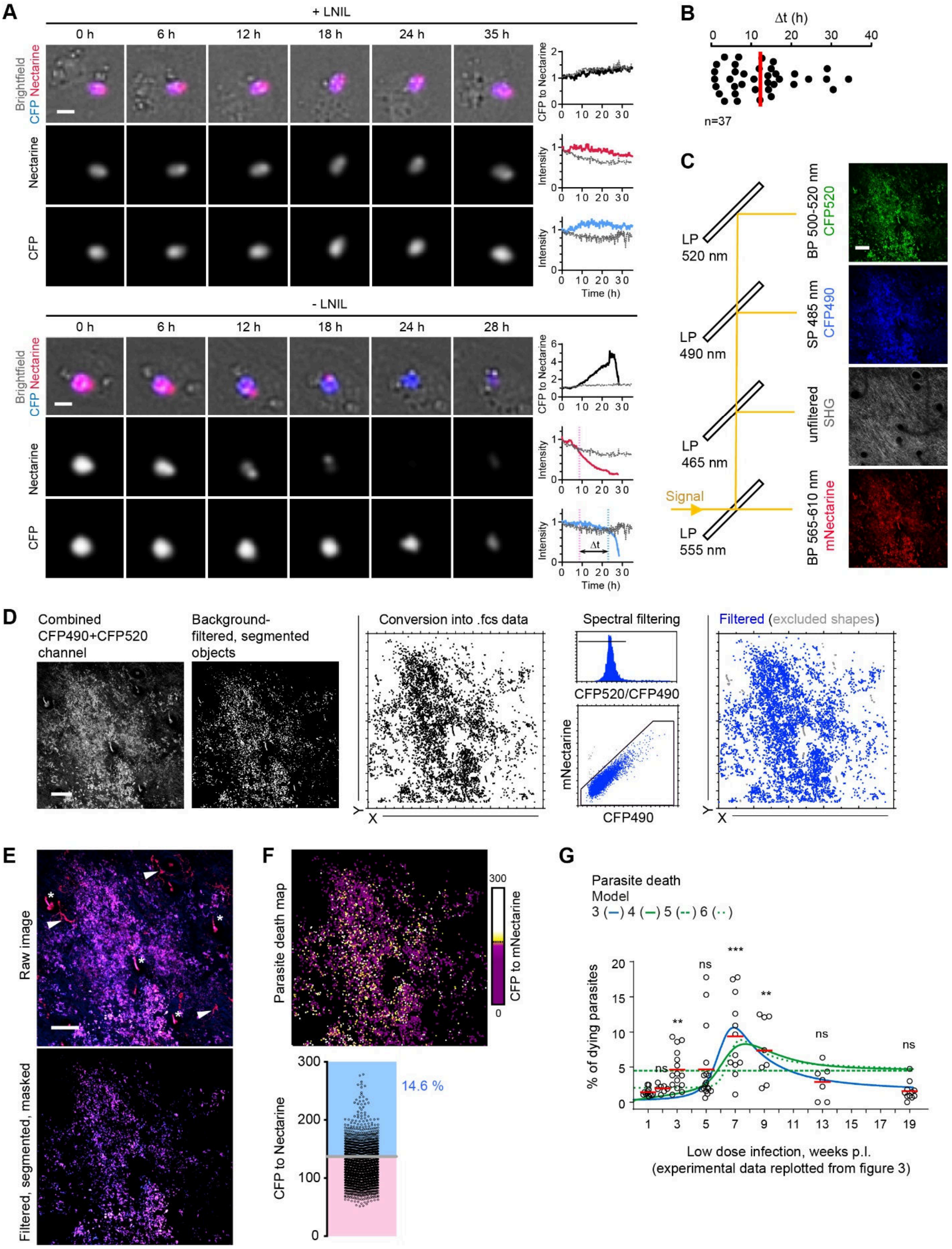

A. Time-lapse microscopy images of IFNγ/LPS activated peritoneal macrophages infected with *L. major*^{necR} with (upper panels) and without (lower panels) inhibition of iNOS-dependent parasite killing by L-NIL treatment. Curves on the right show the quantification over time of Nectarine (red) and CFP (blue) fluorescence intensity as well as corresponding CFP/Nectarine ratio (black). Parasites from LNIL-treated cultures (n=11) were used as controls to determine the 95% confidence interval within which CFP and Nectarine fluorescence values of viable parasites are expected to lie. Grey curves show the lower limit of the confidence interval calculated for Nectarine and CFP fluorescence and the upper limit of the confidence interval determined for CFP/Nectarine ratio. Scale bar, 5 µm. **B.** Time calculated between the significant loss of Nectarine fluorescence, indicating loss of membrane integrity, and loss of CFP fluorescence, indicating parasite disruption. Each dot represents one tracked parasite disappearance event as shown in (A). The vertical bar denotes the mean. **C.** Configuration of the dichroic mirrors and filters used to image ear tissue infected with the *L. major*^{necR} reporter strain. Scale bar, 100 µm. **D.** Representative examples of the combined channel calculated to achieve gradient-based segmentation of the intravital images, XY map representation of the segmented objects after data conversion into flow cytometry data, and spectral filtering strategy used in FlowJo to eliminate autofluorescent objects (hair or cells) associated with high CFP520/CFP490 and mNectarine/CFP490 ratio. Selected objects appear blue, rejected objects are shown in grey. Scale bar, 100 µm. **E.** Representative examples of merged CFP490 and mNectarine channels before (upper panel) or after segmentation and spectral filtering (lower panel). Scale bar, 100 µm. **F.** Upper panel: Representative example of a heat map generated after quantification of parasite viability and corresponding graph. Viable parasites are displayed in purple, dying parasites are shown in yellow or white. Lower panel: representation of the CFP/mNectarine ratio of individual parasites detected within the analyzed site of infection. The grey bar denotes the viability threshold, each symbol represents one parasite. **G.** Overlay of ODE-modelled fractions of dying parasites using models 3 through 6 (see figure 1, supplemental figure S1). Experimental data (circles) are replotted from figure 3).

Supplemental figure S4. Modelling of parasite proliferation in the ongoing infection. Related to figure 4.

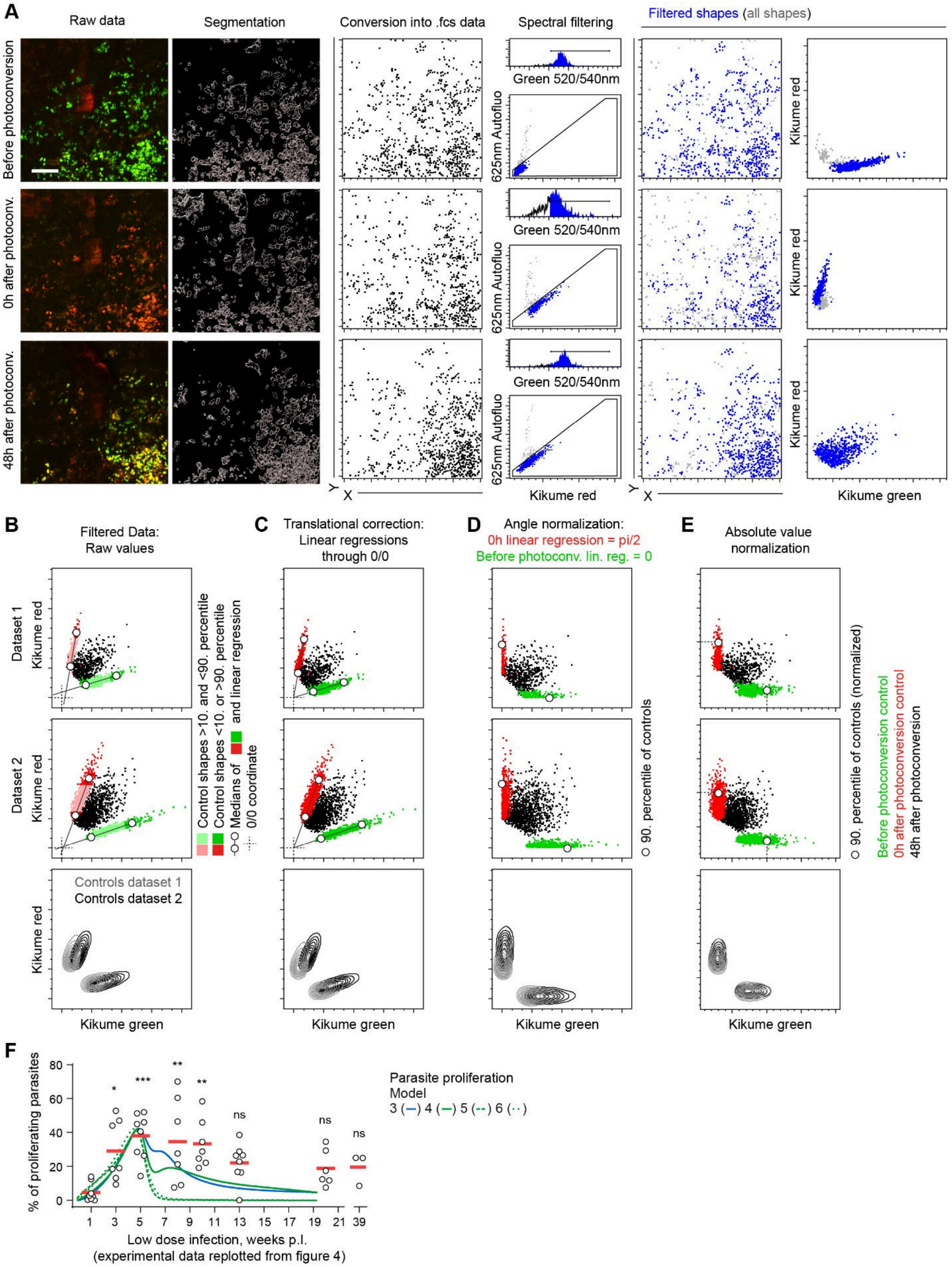

A. Fluorescence data extraction and filtering strategy. An example of data from the same site of imaging before (top), 0 h after photoconversion (middle) and 48h after photoconversion (bottom) is shown. Segmented shapes from the Imaris software are shown, which were converted into flow cytometry data. Spectral filtering was done sequentially, first selecting objects with a high ratio of 520/540 nm emission, and then selecting objects with a high Kikume Red (560-625 nm) to far red autofluorescence (>625 nm) ratio. Positively selected populations are shown in blue. Scale bar, 50 µm. **B.** Determination of slopes of the preconversion (green) and 0 h after photoconversion (red) control populations by determining the median (circles) red and green values of the 10th and 90th percentile in the control populations (percentiles determined according to the Kikume green in the preconversion control, according to Kikume red in the 0h after photoconversion control). The data of two independent examples (top and middle panel) are shown, as well as the overlay of the contour plots of the preconversion and 0h after photoconversion control (bottom panel). **C.** Lateral correction: linear regression curves run through 0/0 (dotted lines). **D.** Angle normalization: linear regression curves of preconversion and 0h after photoconversion controls run at a slope angle of 0 and pi/2, respectively, through the 0/0 coordinate. Circles denote the 90th percentile red fluorescence of the 0h after photoconversion and 90th percentile green fluorescence of the preconversion control. **E.** Normalization of the data to the 90th percentile of the control fluorescences. **F.** Overlay of modelled fractions of proliferating parasites using models 3 through 6 (see figure 1, supplemental figure S1). Experimental data (circles) replotted from figure 4).

Supplemental figure S5. Inhibition of cell recruitment to the site of infection. Related to figure 5.

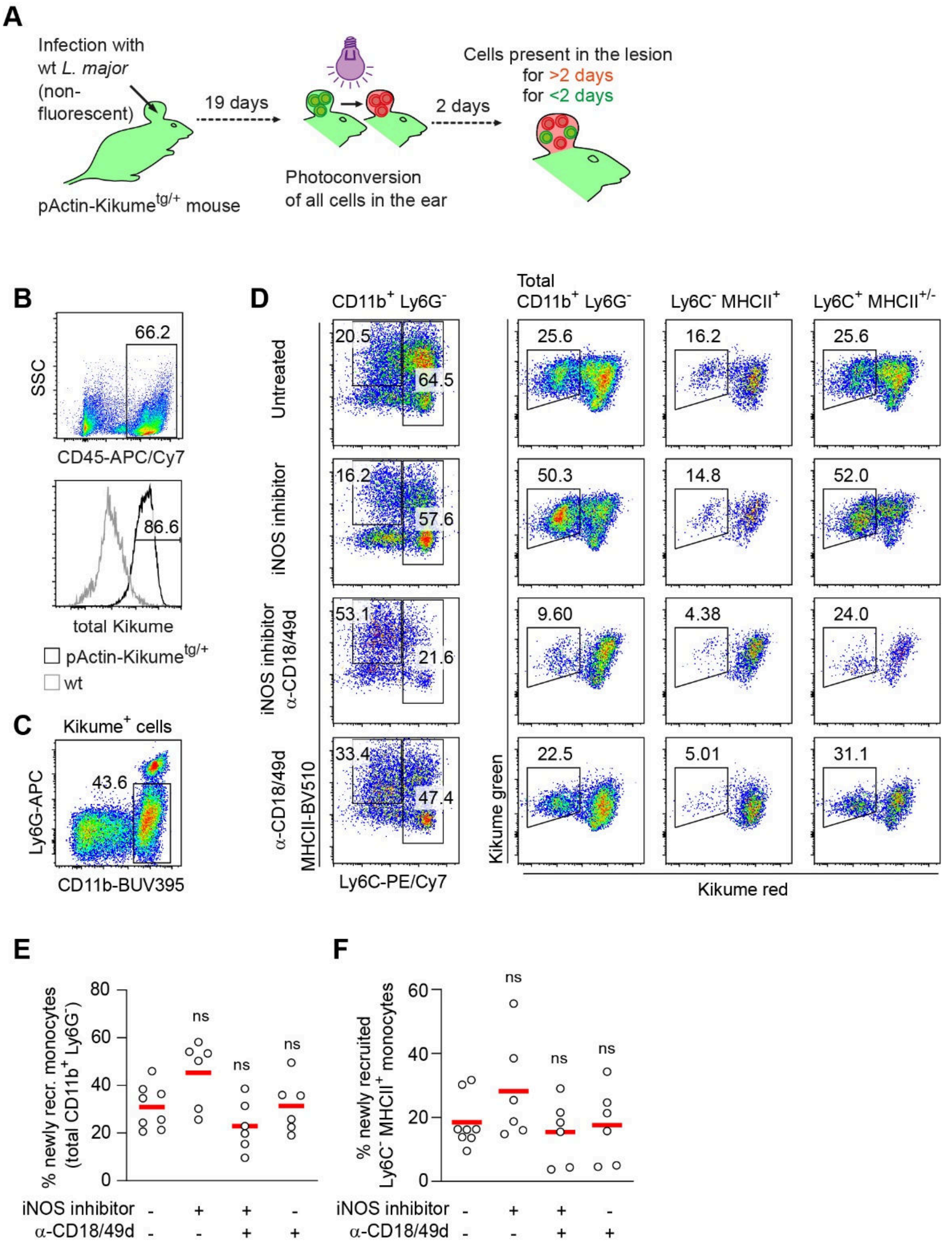

A. Experimental setup for determining the proportion of newly recruited cells in the infected ear. **B-C.** Gating strategy. (B) Kikume-expressing cells were gated from CD45^{+} leukocytes using a calculated total Kikume fluorescence channel. (C) From the resulting Kikume^{+} cells, CD11b^{+}Ly6G^{-} monocyte-derived non-neutrophilic phagocytes were analyzed. **D.** Analysis of recently recruited Ly6C^{+}MHCII$^{+/-}$ and matured Ly6C^{-}MHCII^{+} monocyte-derived non-neutrophilic phagocytes with and without iNOS inhibition by L-NIL and with and without

blocking of phagocyte recruitment. **E-F.** Fraction of total $CD11b^{+}Ly6G^{-}$ (E) and $Ly6C^{-}MHCII^{+}$ cells (F) recruited within the last 2 days before the end of the experiment. Each dot represents an individual ear, data pooled from two independent experiments. Horizontal bars denote the mean; ns, not significant according to one-way ANOVA with Dunnett's post test (comparison with no inhibitor/no antibody control).

Supplemental figure S6. Pathogen burden development under L-NIL with and without blocking of immune cell recruitment. Related to figure 6.

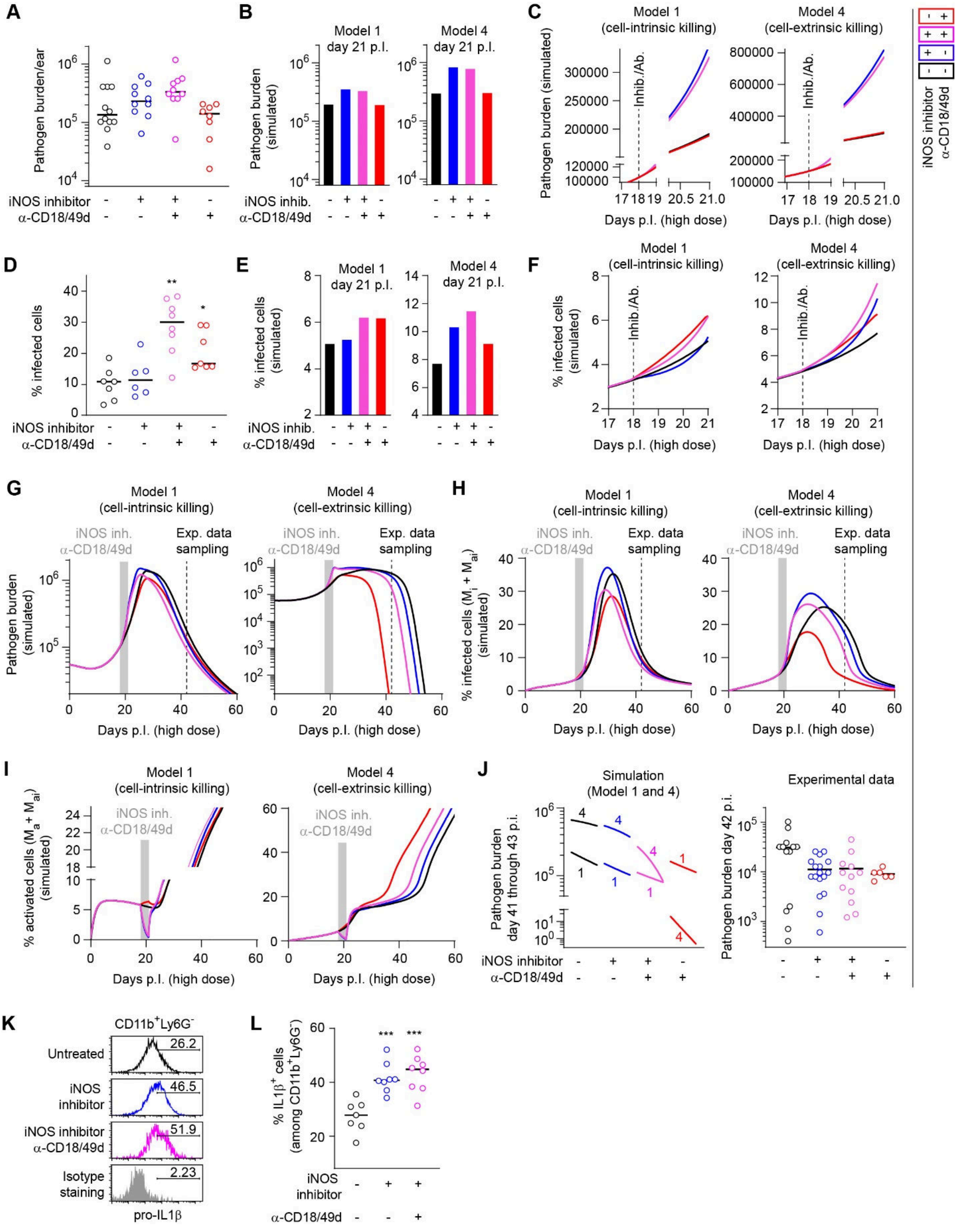

A. Pathogen burden determined experimentally using limiting dilution analysis at 3 wpi (high dose) with and without iNOS inhibition by L-NIL, and/or antibody-mediated blocking of immune cell recruitment from 3 days before analysis. Each symbol represents one infected ear, data are pooled from 4 independent experiments. Horizontal lines denote the median. **B.** Simulated pathogen burden in the ODE model of cell-intrinsic killing (left panel) or cell-extrinsic killing (right panel). Simulated *L. major* numbers shown at day 21 p.i. for untreated (black bars), activation-inhibited (blue bars), recruitment-inhibited (red bars) or a combined inhibition (pink bars) conditions, with treatment initiated at day 18 p.i. **C.** Simulation of pathogen burden in the ODE model of cell-intrinsic (left) or cell-extrinsic (right) killing of *L. major* in untreated (black curves), activation-inhibited (blue curves), recruitment-inhibited (red curves) or combined inhibition (pink curves) for three days, starting from day 18 p.i. **D.** Fraction of infected cells determined by flow cytometry under the conditions as described in (A). Each symbol represents one infected ear, data are pooled from 2 independent experiments. Horizontal lines denote the median. **, $p<0.01$; *, $p<0.05$ according to Kruskal-Wallis test with Dunn's post test (comparison with no inhibitor/no antibody control). **E-F.** Simulated fraction of infected cells in the ODE model as described in (B-C). **G-I.** Simulation of pathogen burden (G), total fraction of infected cells (H), and total fraction of activated cells (I) over 60 days in the ODE model of cell-intrinsic (left panels) or cell-extrinsic (right panels) killing in untreated (black curves), activation-inhibited (blue curves), recruitment-inhibited (red curves) or combined inhibition (pink curves) for three days. The inhibition (grey interval) was modeled to take effect for 3 days, starting from day 18 p.i. A vertical dashed line denotes day 42 p.I., for which time point the modelling predictions were tested experimentally. **J.** Left panel, simulated pathogen burden for model 1 and model 4 in the conditions described under (G-H), curves show the time course from day 41 to day 43. Right panel, pathogen burden determined by limiting dilution analysis at day 42 p.i., after treatment from day 18 p.i. on with three doses of L-NIL (blue), one dose of anti-CD18/CD49d (red), both (pink), or not treated (black). Horizontal bars denote the median. **K-L.** Flow cytometry analysis of pro-IL1β in $CD11b^{+}Ly6G^{-}$ phagocytes isolated from the site of infection of control (black curve and symbols), L-NIL treated (iNOS inhibition, blue curve and symbols) and L-NIL/anti-CD18/49d treated (pink curve and symbols) infected mice. The grey histogram shows an isotype control for the pro-IL1β staining. Each symbol represents one infected ear. Horizontal bars represent the mean. ***, $p<0.001$ according to one-way ANOVA with Dunnett's post test (comparison with no inhibitor/no antibody control).

Supplemental figure S7. Ectopic phagocyte entry in late *L. major* infection. Related to figure 7.

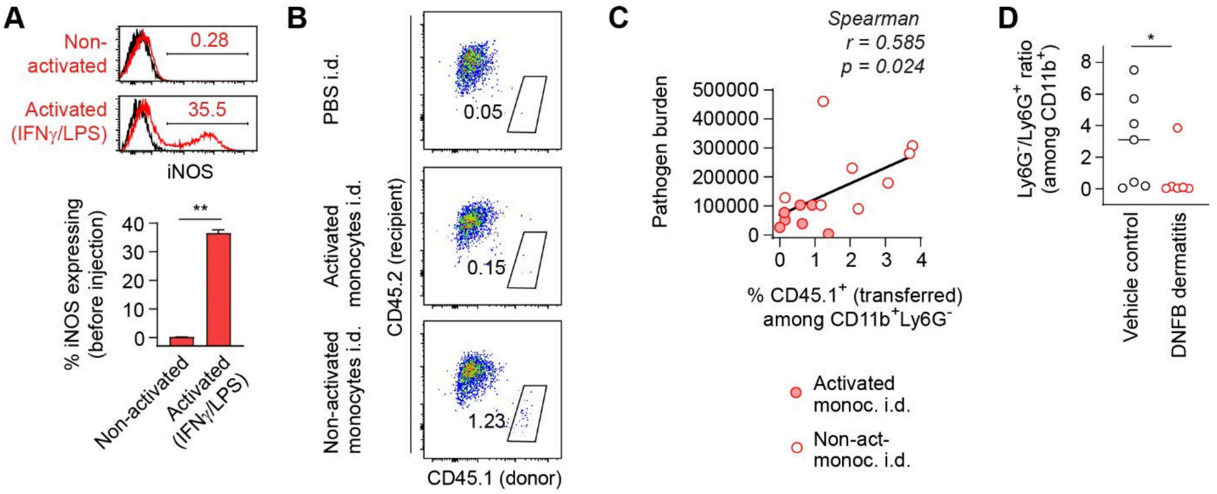

A. Analysis of iNOS expression in bone marrow-isolated monocytes with and without IFNγ/LPS activation prior to injection into 10 wpi infection sites.Red curves; anti-iNOS stained samples; black curves: isotype control staining. Bar diagram shows the mean + standard deviation of 5 replicate analyzed. **, p<0.01 according to Mann-Whitney test. **B.** Flow cytometry analysis of CD45.1 (donor) and CD45.2 (recipient) ratios among CD11b$^+$Ly6G$^-$ phagocytes isolated from the site of a 10 week infection injected i.d. with PBS (upper panel), activated (middle panel) or non-activated monocytes (lower panel) in the infected ear 3 days before analysis. **C.** Correlation between the pathogen burden and fraction of monocytes detected 3 days after i.d. deposition into the site of injection. **D.** Ratio of Ly6G$^-$ to Ly6G$^+$ cells among CD11b$^+$ phagocytes isolated from a 10 week infection site treated with vehicle solution (black symbols) or DNFB in order to induce contact dermatitis three days before analysis. Each symbol represents one individual infected ear. Data pooled from two independent experiments. The median is shown by a horizontal line. *, p<0.05 according to Mann-Whitney test.
